# Supplementary material for: Examining the role of common variants in rare neurodevelopmental conditions
Source: Nature. 2024 Nov 20;636(8042):404–11. doi: 10.1038/s41586-024-08217-y (PMC11634775; doi:10.1038/s41586-024-08217-y)
Supplement: Supplementary file 1 — Supplementary Methods, Figs. 1–21, Notes 1–9, Descriptions of Tables 1–21 and Descriptions of Data 1–3. [file 41586_2024_8217_MOESM1_ESM.pdf]

nature portfolio

https://doi.org/10.1038/s41586-024-08217-y

Supplementary information

Examining the role of common variants in rare neurodevelopmental conditions

In the format provided by the authors and unedited

Supplementary Information

Supplementary Methods 1
- Quality control of genetic data 1
- Identifying relatives across cohorts 7
- Defining trio sample sets for analysis in DDD and GEL 8
- Decomposition of genetic correlation using GenomicSEM 9
- Calculating polygenic scores 10
- Analyses of polygenic scores 12
- Construction and incorporation of weights for the Millennium Cohort Study 13
- Enrichment of gene sets and pathways 14

Supplementary Figures 16
Supplementary Notes 37
- Supplementary Note 1: Lay summary and Frequently Asked Questions 37
- Supplementary Note 2: Phenotypic comparisons of the cohorts 54
- Supplementary Note 3: Genome-wide significant hits from the GWAS meta-analysis of neurodevelopmental conditions 55
- Supplementary Note 4: Potential ascertainment biases in control cohorts and their effects 56
- Supplementary Note 5: Examining sex differences in polygenic risk 58
- Supplementary Note 6: Results for polygenic scores based on the within-family GWAS of educational attainment 59
- Supplementary Note 7: Exploring the role of prenatal risk factors in mediating common variant risk 60
- Supplementary Note 8: Role of PGS in modifying the penetrance of rare variants 61
- Supplementary Note 9: Genes and pathways affected by common and rare variants associated with NDCs 62

Descriptions of Supplementary Tables 64
Descriptions of Supplementary Data 68
Supplementary References 68

Supplementary Methods

Quality control of genetic data

DDD

The DDD cohort was genotyped on three genotyping arrays: the Illumina HumanCoreExome chip (CoreExome), the Illumina OmniChipExpress chip (OmniChip), and the Illumina Infinium Global Screening Array (GSA). Some probands were genotyped on more than one chip, as shown in **Supplementary Figure 9**.

CoreExome and OmniChip

Quality control (QC) of CoreExome (including DDD patients and 9,270 UKHLS controls genotyped on the same chip) and OmniChip data were performed by Niemi *et al*. in each dataset separately[1]. Briefly, samples with sex discrepancies, high missingness (≥3%) in variants with MAF ≥10%, or high or low heterozygosity (+/- 3 SDs from the mean) were removed. Individuals who had genetically inferred white European ancestry, defined using reference samples from the 1,000 Genomes project, were kept. One individual from each pair of related individuals (identical by descent ≥12% using PLINK) were removed from amongst the CoreExome samples, and those who were related to trios genotyped on Omnichip were also removed. Trios with > 2,000 Mendelian errors were removed. We removed variants with minor allele frequency (MAF) < 0.5%, missingness ≥3%, or a Hardy-Weinberg equilibrium (HWE) test p-value < $1x10^{-5}$. Variants with matched alleles between DDD CoreExome and UKHLS were kept so that imputation could be conducted from a common set of variants for both cohorts.

Global Screening Array

Global Screening Array (GSA) samples from DDD were genotyped in two batches. We removed samples that were discordant with exome sequencing data previously generated on all DDD individuals[2]. Sample swaps, duplicates, those with sex discrepancies, and missingness ≥ 5% were removed. We examined heterozygosity rate and removed outliers with a rate < 0.158 or > 0.170. Palindromic, duplicated and multiallelic variants were removed, as well as indels. Variants with either a call rate < 95% or with significant deviation from HWE ($p < 1\times10^{-6}$) were also removed. The two batches were then merged to overlapping SNPs. Variants with a significantly different genotype rate ($p < 1\times10^{-50}$) and allele frequency between the two batches were removed. Trios with > 200 Mendelian errors were removed, as were SNPs with Mendelian errors in >1% of trios. Variants were again filtered to remove those with a significant deviation from HWE ($p < 1\times10^{-6}$) and subset to those with a MAF > 1%. The number of samples remaining before and after these QC steps are given in **Supplementary Tables 13 and 14**.

To identify GSA individuals of genetically inferred European ancestry, we first projected the post-QC samples (N=9,572) onto 1,000 Genomes phase 3 individuals[3] using the smartpca function from EIGENSOFT version 7.2.1[4]. We used linkage disequilibrium (LD)-pruned SNPs (pairwise r^2 <0.2 in batches of 50 SNPs with sliding windows of 5) with MAF > 5% and removed 24 regions with high or long-range LD, including the HLA[5], leaving 90,563 variants. We identified a subset of samples that projected onto European ancestry samples from 1,000 Genomes (PC2 > 0.0175), leaving 9,534 European ancestry DDD individuals (**Supplementary Figure 10**). We then performed another principal component analysis (PCA) in the unrelated individuals within the loosely-defined European ancestry subset, projecting related individuals onto them using smartpca[4]. We applied Uniform Manifold Approximation and Projection (UMAP)[6] using the first ten PCs and identified a homogeneous subgroup of 8,489 individuals (**Supplementary Figure 11**). Since we intended to merge trio data genotyped on GSA and Omnichip array chips in downstream analysis, we conducted a PCA to further confirm that the GSA individuals were well matched for ancestry with OmniChip individuals previously identified to have GBR ancestry[1] (**Supplementary Figure 12**).

GEL

Variant calling and initial QC were performed by Genomics England. We used 78,195 post-QC germline genomes from the Aggregated Variant Calls (aggV2) prepared by the GEL team. All samples were sequenced with 150bp paired-end reads using Illumina HiSeqX and processed on the Illumina North Star Version 4 Whole Genome Sequencing Workflow, comprising the iSAAC Aligner and Starling Small Variant Caller. Aggregation of single-sample gVCFs were performed using the Illumina software gVCF genotyper. We retained 78,195 samples that passed various QC filters including sample contamination <3%, ratio of SNV heterozygous to homozygous calls <3, total number of SNVs between 3.2 to 4.7 million, array concordance > 90%, and median fragment size >250bp, excess of chimeric reads <5%, percentage of mapped reads >90%, and percentage at dropout < 10%. We kept variants that passed the QC filters shown in **Supplementary Table 15**.

For GWAS and PGS analyses, we applied additional QC following Kousathanas *et al*.[7]. Low-quality genotypes were masked using the bcftools setGT module performed by the GEL team. Specifically, genotypes with depth < 10 or GQ < 20, as well as heterozygous genotypes failing an allele balance binomial test with P-value < $1x10^{-3}$ were set to missing. The masked VCF files were converted to PLINK pgen format using PLINK v2.0[8]. We removed samples with mean autosomal coverage <25x. Within GEL samples who had genetically inferred European ancestry (inferred as described below), we further removed samples that had four median absolute deviations (MADs) above or below the median for the following metrics: ratio of insertions to deletions, ratio of transitions to transversions, total deletions, total insertions, total heterozygous SNPs, total homozygous SNPs, total transitions, and total transversions. For the number of total singletons (SNPs), samples were removed that were more than eight MADs above the median. For the ratio of heterozygous to homozygous SNPs, samples were only removed that were more than four MADs above the median, so as not to remove samples that simply had high autozygosity.

We used GEL individuals with genetically inferred European ancestry, which were identified by the GEL bioinformatics team. A list of high quality LD-pruned autosomal biallelic SNPs were used in ancestry prediction: MAF >5% in both aggV2 and 1,000 Genomes project phase 3, missingness <1%, median GQ ≥30, median depth ≥30, non-palindromic, and HWE test p-value $>1x10^{-5}$. LD pruning with an r2 0.1 in 500kb windows was performed using PLINK 1.9 after removing SNPs located in long-LD regions. GCTA[9] was used to calculate 20 PCs in 1,000 Genome phase 3 samples, and GEL aggV2 samples were projected onto the PC loadings. A random forest model based on eight PCs was trained to assign an individual's probability of being from 1,000 Genomes super-populations. A cut-off of 0.8 was used to identify individuals of European ancestry. PCA within the GEL individuals predicted to have European ancestry showed population structure within this group (**Supplementary Figure 13**). To obtain a homogeneous subset that represents white British individuals, we kept samples with a probability of being from the 1,000 Genomes GBR-ancestry sub-population >0.1. Prediction of 1,000 Genomes sub-populations was performed using a random forest model trained based on PCs calculated within each predicted super-population using high quality SNPs with MAF >1%. We further removed samples with PC2 from the within-European PCA less than -0.015 to make sure the GEL samples were homogeneous (**Supplementary Figure 13**). This left 56,249 individuals in GEL.

ALSPAC

Data we received from ALSPAC were processed in two batches[10]. We had post-QC array data for G0 mothers and G1 children (N=17,816) in the first batch. Mothers (N=8,884) were genotyped on the Illumina Human 660W chip and children (N=8,932) were genotyped on the HumanHap550 quad chip. QC was performed by ALSPAC in the two datasets separately, as follows. Sample QC included removing samples with missingness rate <3%, heterozygosity outliers, or sex mismatches. Variant QC filters included missingness rate <5%, MAF >1%, and HWE test P-value $>1x10^{-6}$. After merging the two datasets, SNPs with missingness rate >1% across all samples were removed.

Another 2,198 parents (G0 mothers and G0 partners) were genotyped on the CoreExome array chip in the second batch. On top of this initial QC that ALSPAC has done, we further removed seven samples with high missingness (>3%). We kept autosomal SNPs with MAF >0.5%, missingness rate <3%, and that passed the HWE test (p-value > $1x10^{-5}$). Array data received from the ALSPAC all had genetically predicted European ancestry, so we did not perform any filtering based on genetic ancestry.

We merged the two batches and estimated genetic kinship to check sample swaps. We used KING[11] to estimate kinship using 113,090 overlapping SNPs. We removed 152 samples who had unexpected first-degree relationships but were from different families. In addition, we removed 16 samples who did not match available exome sequencing data supposedly for the same individuals; we did not remove array samples with mismatched exome data when they had a first-degree relative in the array dataset who could confirm their kinship. This left 17,656 and 2,183 samples from the two batches respectively. Among them, 8,831 were children, 9,302 were mothers, and 1,706 were fathers.

MCS

Genotype chip data

We received data from MCS for 21,181 samples genotyped using the GSA array chip that passed initial sample QC[12]. Samples with missingness rate > 20%, high or low heterozygosity (+/- 5 SDs), and sex mismatches were removed. We kept autosomal SNPs with missing rate <5%, MAF >0.5%, and had HWE test p-value > $1x10^{-5}$ in a subset of unrelated individuals whose genetically inferred ancestry was similar to European samples from the 1,000 Genomes project. (See below for how ancestry was predicted). For duplicated variants, we kept the one with higher call rate. We further removed 283 samples with missingness rate >5%.

To identify MCS individuals with genetically inferred European ancestry, we performed a PCA in 1,000 Genomes Project phase 3 samples and projected MCS samples onto the PC space. In the PCA, we used SNPs with MAF >5% and missingness rate <1% that passed a lenient HWE test (p-value > $1x10^{-20}$) in all MCS samples from diverse ancestries. We matched with SNPs that passed similar QC in the 1,000 Genomes. LD pruning was performed in MCS with r^2 <0.2 in windows of 50 SNPs. This left 87,738 SNPs in the PCA. We applied UMAP[6] using the first four PCs which differentiated continental-level populations in the 1,000 Genomes project (**Supplementary Figure 14**). 17,599 samples clustered together with non-Finnish European samples from the 1,000 Genomes project. Among them, 17,288 individuals were reported to

have White ethnicity, and we restricted to these. To get a homogeneous subset, we performed another PCA within these 17,288 samples by projecting the relatives onto the PC space calculated from the unrelated subset (**Supplementary Figure 15A**). We performed UMAP on four PCs, and kept 16,803 samples from the main cluster (**Supplementary Figure 15B**).

We next checked sample swaps using genetically inferred kinship between family members estimated using KING[11]. We removed individuals whose family relationship data were missing and individuals who had unexpected first-degree relatives: parent-offspring pairs within a family that were not confirmed by genetic data, individuals from different families had a parent-offspring relationship, singleton probands who had a full sibling assigned with a different family ID. This left 16,634 individuals; among them 6,153 were children, 6,646 were mothers and 3,835 were fathers.

Exome sequence data

14,791 individuals from MCS, including 7,807 children and 6,975 of their parents, were exome-sequenced using TWIST capture baits (Twist Custom Panel: Core exome plus Broad panel; Twist Design ID: NGSTECustom_0001418) and Illumina NovaSeq S4 100PE, to an average depth of ~68X. We removed samples with VerifyBamID score > 0.05 due to having possible contamination. BWA-MEM was used to map the reads to GRCh38 with BWA-MEM, then SNV and indel calling was conducted with GATK HaplotypeCaller, GenomicsDBImport and GenotypeGVCFs (GATK version 4.2.4.0), following GATK best practices. Hail v0.2.105 was used to conduct sample, variant and genotype QC, as described below.

Sample QC

For the purposes of sample QC, we first filtered the data to include only biallelic SNVs and to remove variants with an internal allele frequency of ≤ 0.001 and variants with a call rate of ≤ 0.99, which reduced the number of variants from 4,920,291 to 386,148. We merged the MCS data with data from 1,000 Genomes phase 3, retaining variants present in both. We then removed variants that had low call rate (< 0.99), low allele frequency (< 0.05) or low Hardy-Weinberg equilibrium p-value (< $1x10^{-5}$), variants in long range linkage disequilibrium regions and palindromic SNVs. We ran a PCA using Hail's hwe_normalized_pca function, and then used gnomad's `assign_population_pcs` function on first ten principal components to predict which superpopulation (European, South Asian, East Asian, African, American, or other) each MCS sample was most similar to. 12,851 MCS samples were assigned as being most similar to the European samples from 1,000 Genomes.

Next we ran the `sample_qc` function in Hail and stratified the output by superpopulation. We first removed calls with DP (depth) < 20, GQ (genotype quality) < 20 or VAF (variant allele fraction) < 0.25, and then calculated the following metrics per sample: number of SNVs, Transition/Transversion ratio, het/hom ratio, heterozygosity rate, number of transitions, number of transversions, number of insertions, number of deletions, and insertion/deletion ratio. We filtered out 302 samples who fell outside of the median +/-4 median absolute deviations compared to samples from the same superpopulation for at least one metric.

Variant and genotype QC

For variant QC, we used a random forest model trained on various metrics to distinguish likely true positive from likely false positive variants. Variants in the following high-quality datasets were identified in our data and treated as true positive variants:

- High confidence sites discovered in 1,000 Genomes
- SNVs found in 1,000 Genomes that are present on the Omni 2.5 genotyping array
- Indels present in the Mills and Devine data[13]
- SNVs and indels from HapMap3

As false positive variants, we took variants failing this set of hard filters: QD (quality by depth) < 2 or FS (FisherStrand i.e. Phred-scaled p-value of Fisher's exact test to detect strand bias) > 60 or MQ (mapping quality) < 30.

We trained a random forest model on chromosome 20 using the true positive and false positive annotations above, then applied it to the whole dataset. Most of the features used in the random forest were those used by gnomAD, and they are listed here: QD (quality by depth), meanHetAB (mean heterozygous allele balance), is_CA (is a C>A SNV), SOR (strand odds ratio), variant_type (SNV/indel/multiallelic SNV/multiallelic indel/multiallelic mixed), ReadPosRankSum (Rank sum test for relative positioning of REF versus ALT alleles within reads), was_split (is a multiallelic site), has_star (alleles at this side include a '*' allele), n_alt_alleles (number of ALT alleles at a site), MQ (mapping quality), MQRankSum (Rank sum test for mapping qualities of REF versus ALT reads), allele type (SNV/insertion/deletion), and was_mixed (Multiallelic site containing SNV(s) and indel(s)) .

We included the metrics is_CA and meanHetAB in order to remove a specific artefact in the dataset characterized by a preponderance of C>A errors, which arose through a step in library preparation.

We ranked the variants by their random forest score and binned them. To decide on provisional random forest score thresholds for SNVs and indels, we manually evaluated plots of the cumulative number of true positive variants per bin and the cumulative number of false positive variants per bin (for both SNVs and indels), and of the transmitted/untransmitted ratio for synonymous singletons (SNVs only; i.e. those seen in only one parent in the sample, using 3,132 trios).

To decide on suitable hard filters for variants and genotypes, we tested different combinations of random forest bin (i.e. a variant-level metric) with various genotype quality metrics: DP (depth), GQ (genotype quality) and HetAB (heterozygous allele balance i.e. the fraction of reads carrying the ALT allele at a heterozygous genotype). For variants passing a given random forest bin filter, we set genotypes to missing if they had GQ, DP or HetAB less than the specified threshold. For each combination of filters, we calculated various metrics:

- precision and recall of variants found in the Genome in a Bottle sample NA12878
- the proportion of true positive and false positive variants from the random forest annotation remaining
- the ratio of transmitted to untransmitted variants for synonymous singletons

For SNVs, the final filters chosen were: random forest bin < 82, DP < 5, GQ < 15, and HetAB < 0.2. This gave a precision and recall of 0.931 and 0.953 respectively, captured 97.87% of true

positives and 0.27% of false positives, and gave a transmitted:untransmitted ratio of 0.998 for synonymous singletons. For indels, the final filters were: random forest bin < 58, DP < 10, GQ < 20, and HetAB < 0.3. This gave a precision and recall of 0.774 and 0.691 respectively, and retained 91.185% of true positives and 0.274% of false positives.

Identifying relatives across cohorts

Identifying DDD-GEL duplicates and relatives

We suspected that there would be overlapping patients and relatives between DDD and GEL, and wanted to remove these to ensure that the samples analysed from the two cohorts were independent. Since the GEL data cannot be removed from the GEL Research Environment, we moved DDD genetic data from English and Welsh samples into it after obtaining ethical permission. We did not have consent to move DDD Scottish samples to the GEL Research Environment, so we could not remove GEL samples who were related to them. Thus, we removed Scottish individuals from the DDD cohort in GWAS and PGS analyses, and focused on identifying and removing GEL participants who were duplicates of or related to the remaining individuals from DDD. We used two approaches to identify DDD duplicates, siblings and more distant relatives in GEL (described below), and removed GEL individuals who were identified as related to DDD individuals by either approach.

We first used the DDD exome-sequence data to identify overlapping samples between DDD and GEL, and pairs of individuals from the two cohorts who were siblings. DDD exome-sequenced samples (N=32,369) were used to create informative genotype barcodes (2,466 SNPs). After excluding probands from Scotland, Northern Ireland and Dublin (whom we assumed would have not have been cross-recruited to GEL), 11,941 DDD probands were transferred to the GEL Research Environment and matched using bcftools gtcheck -e 0 --genotypes DDD.bcf GEL-sample.vcf.gz. To account for missing data, the average number of mismatching genotypes was then used to determine identical samples and siblings. We removed 1,752 GEL patients who were identified as duplicates of DDD probands and ten GEL participants who were identified as siblings of DDD patients. (Using the array data which had higher resolution (see below), two of the putative duplicates and one of the putative siblings were subsequently determined to actually be siblings and second-degree relatives of the DDD patients, respectively.)

We moved array data for 18,569 participants from DDD excluding those recruited from Scottish centres and 9,270 UKHLS controls into the GEL Research Environment. We matched SNPs from the three array chips with GEL, which left 85,092 SNPs. Up to third-degree relatives (kinship coefficient > 0.0442) were identified using KING v2.2.4[11]. Among the GEL participants with genetically-predicted European ancestry, we further removed 2,525 individuals who were related to DDD participants as well as 235 individuals who were related to UKHLS controls.

Integration of birth cohorts and DDD

We removed individuals from ALSPAC and MCS who were related to each other (across cohorts) or to DDD individuals. To do that, we first merged array data across DDD (CoreExome, GSA, and Omnichip), ALSPAC (both batches), and MCS (GSA). There were 45,295 SNPs that passed QC in all datasets. We compared the kinship estimated using this list of overlapping

SNPs with that estimated from all available SNPs within MCS, and found that we could infer kinship relationships up to second-degree relatives accurately using the small number of overlapping SNPs. We then removed 33 samples from ALSPAC and 46 samples from MCS that had a second-degree relative or closer in DDD. We had 1,459 and 2,523 parent-offspring trios in ALSPAC and MCS, respectively. We finally removed samples from MCS or ALSPAC to make sure that children in those trios from both cohorts were unrelated, and that parents in the trios were also unrelated with other parents, resulting in 1,434 and 2,498 trios from ALSPAC and MCS, respectively.

Defining trio sample sets for analysis in DDD and GEL

The procedure used for filtering trios used in DDD and GEL is shown in **Supplementary Figure 16** and described below.

DDD

We combined trios with GBR ancestry across GSA and OmniChip arrays, then kept unrelated trios (up to three degrees of relatedness, as determined using KING[11]). We removed trios recruited from Scottish centres for the reason described above in the section on "Identifying relatives". We then subset to trios where the proband had a neurodevelopmental condition. We then split trios into those with both parents unaffected and those with one or both parents affected. These were then categorised as genetically diagnosed or undiagnosed. Among the undiagnosed trios, we looked at trios in which the proband had a rare inherited damaging variant in either a constrained gene or a dominant DD-associated gene with a loss of function mechanism. We also looked at trios with a *de novo* diagnosis.

GEL

We used participant data and aggregate gVCF sample statistics from the GEL main programme version 13. We identified trios, and subset to those in which each member was whole genome sequenced and the genetically-inferred sex was consistent with the phenotypic sex. We then removed trios in which any member was already in DDD or related to DDD participants up to three degrees of relatedness. These were further subset to trios in which all individuals had GBR ancestry and probands had a neurodevelopmental condition. We then kept the maximal number of trios for which the probands were not related with each other (up to third degree) and none of the parents were related to another parent. We then identified trios with unaffected parents and trios with one or both parents affected. These were then categorised into genetically undiagnosed and diagnosed. Similar to DDD, we looked at undiagnosed trios in which the proband had a rare damaging variant, as well as trios with a *de novo* diagnosis.

Decomposition of genetic correlation using GenomicSEM

We used the GenomicSEM model without SNP effects to estimate the percentage of the genetic correlation between NDCs and brain-related traits of interest that is explained by latent variables representing the educational attainment (EA) and non-EA components of NDCs. In the GenomicSEM model shown in **Supplementary Figure 17** (modified from Figure 1 in Demange *et al.*[14]), observed EA is influenced by the latent EA variable with an effect size of β_1 and observed NDCs are influenced by both the latent EA variable with an effect size of β_2 and

the latent non-EA variable with an effect size of β_3. The model assumes that the covariance of the two latent variables is 0, and the variances of the latent variables are fixed to 1. All variances of EA and NDCs are explained by the latent variables and the residual variances are fixed to 0, thus we have:

$$EA \ = \beta_1 \times L(EA)$$
$$NDCs \ = \beta_2 \times L(EA) + \beta_3 \times L(NonEA)$$

where EA and NDCs are the genetic components of the observed phenotypes in the original GWASs and L(EA) and L(NonEA) are latent variables.

SNP heritability of EA and NDCs can be estimated as:

$$h^2(EA) \ = \ {\beta_1}^2$$
$$h^2(NDCs) \ = \ {\beta_2}^2 + {\beta_3}^2$$

The genetic correlation (r_g) between NDCs and EA is:

$$r_g(NDCs, EA) = \frac{cov(NDCs, EA)}{sd(NDCs) \ \times \ sd(EA)}$$
$$= \frac{cov(\beta_2 \times L(EA) + \beta_3 \times L(NonEA), \beta_1 \times L(EA))}{sd(\beta_2 \times L(EA) + \beta_3 \times L(NonEA)) \ \times \ sd(\beta_1 \times L(EA))}$$
$$= \frac{cov(\beta_2 \times L(EA), \beta_1 \times L(EA))}{\sqrt{var(\beta_2 \times L(EA)) + var(\beta_3 \times L(NonEA))} \ \times \ \beta_1 \times \ sd(L(EA))}$$
$$= \frac{\beta_1 \ \times \ \beta_2 \ \times \ cov(L(EA), L(EA))}{\sqrt{{\beta_2}^2 \ \times \ var(L(EA)) \ + \ {\beta_3}^2 \ \times \ var(L(NonEA))} \ \times \ \beta_1 \times \ sd(L(EA))}$$
$$= \frac{\beta_1 \ \times \ \beta_2}{\sqrt{{\beta_2}^2 \ + \ {\beta_3}^2} \ \times \ \beta_1}$$
$$= \frac{\beta_2}{\sqrt{{\beta_2}^2 \ + \ {\beta_3}^2}}$$

GenomicSEM performs LD score regression to estimate the SNP heritability $h^2(EA)$ and $h^2(NDCs)$, and the genetic correlation $r_g(NDCs, EA)$, thus we can estimate the effect sizes as:

$$\beta_1 = \sqrt{h^2(EA)}$$
$$\beta_2 = r_g(NDCs, EA) \ \times \sqrt{h^2(NDCs)}$$
$$\beta_3 = \sqrt{h^2(NDCs) - {\beta_2}^2} = \sqrt{h^2(NDCs) - r_g(NDCs, EA)^2 \times h^2(NDCs)}$$

When we add a third trait X (e.g. ADHD) to the model, we assume that the genetic covariance between NDCs and X is still explained by the two pathways through the two latent variables (**Supplementary Figure 17**):

$$cov(T, NDCs) = cov \ (T, \beta_2 \times L(EA) + \beta_3 \times L(NonEA) \ )$$
$$= cov \ (T, \beta_2 \times L(EA)) + cov(T, \beta_3 \times L(NonEA))$$
$$= \beta_2 \times cov \ (T, L(EA)) + \beta_3 \times cov(T, L(NonEA))$$

We can estimate the percentage (P) of the genetic correlation between NDCs and X that is explained by L(EA) as shown in **Supplementary Figure 1A** and **Extended Data Figure 9B** as:

$$P = \frac{\beta_2 \times\ r_g\ (T, L(EA))}{\beta_2 \times\ r_g\ (T, L(EA)) + \beta_3 \times\ r_g\ (T, L(NonEA))}$$

and the percentage of the genetic correlation that is explained by L(NonEA) as:

$$1 - P = \frac{\beta_3 \times\ r_g\ (T, L(NonEA))}{\beta_2 \times\ r_g\ (T, L(EA)) + \beta_3 \times\ r_g\ (T, L(NonEA))}$$

where the genetic correlation between trait X and the EA component of NDCs and that between trait X and the non-EA component of NDCs can be estimated from the GenomicSEM analysis (the latter is shown in **Figure 1B**). Note that we did not calculate the percentages for traits that did not show a significant genetic correlation with NDCs, or when $\beta_2 \times r_g\ (T, L(EA))$ and $\beta_3 \times r_g\ (T, L(NonEA))$ have opposite signs[14].

Similarly, we estimated the percentage of the genetic correlation between NDCs and the target trait X that is explained by the cognitive (as shown in **Supplementary Figure 1B** and **Extended Data Figure 9C** as) and non-cognitive components of NDCs when considering the GWAS of cognitive performance in the GWAS-by-subtraction analysis.

Calculating polygenic scores

Weights for polygenic scores were estimated using LDPred[15]. We used an LD reference panel for 1,054,330 HapMap3[16] variants based on 5,000 unrelated individuals of white British genetically-inferred ancestry from the UK Biobank[17].

For all scores except that for schizophrenia, we used an LD radius (--ldr) of M/3000, where M is the number of matched SNPs. In the case of schizophrenia, the singular value decomposition (SVD) did not converge, so we used an LD radius of 300. For all scores, as the traits are highly polygenic, we assumed a prior fraction of causal variants to be 1. Once the weights were generated, we calculated polygenic scores for individuals in DDD, GEL, and control cohorts, using the --score function in PLINK v1.9, which calculates the weighted sum of genotypes across a set of SNPs for each individual.

To make PGS comparable across different cohorts, we started from the same 4,570,898 SNPs that were well-imputed in all array cohorts (Minimac4 $R^2 > 0.8$), passed QC in GEL aggV2 samples, and had MAF >1% in all cohorts. Among these, 831,226 SNPs were in the aforementioned LD reference panel. GWAS summary statistics for years of schooling[18], non-cognitive performance of educational attainment[14], cognitive performance[18], schizophrenia[19], and neurodevelopmental conditions[1] were matched with the list of overlapping SNPs (**Supplementary Table 17**). The PGS for neurodevelopmental conditions ($PGS_{NDC,DDD}$) was evaluated in the DDD Omnichip samples and the GEL samples which were not in the GWAS. We also calculated a PGS for educational attainment using a within-sibship GWAS[20].

To ensure PGSs were adjusted for ancestry in a way that was comparable across cohorts (DDD, GEL, UKHLS, MCS and ALSPAC), we performed a joint PCA across all included samples with genetically-inferred European ancestries and adjusted the raw scores for 20 PCs. We firstly performed a PCA in array samples (i.e. all cohorts except GEL). We focused on the 45,295

SNPs that passed QC in all datasets and applied LD pruning (pairwise r^2 <0.5 in batches of 50 SNPs with sliding windows of 5) after removing SNPs in long LD regions, which left 39,784 SNPs in PCA. We calculated PCs in a subset of unrelated individuals (removed up to second-degree relatives) and projected the remaining array samples to the PC space. We then moved the PC loadings to the GEL Research Environment and projected GEL samples with GBR ancestry onto the same PC space. (The rationale for this was that data governance constraints prevented us from moving individual-level ALSPAC and MCS data to the GEL Research Environment.) We regressed out 20 PCs from the raw PGS in array samples and used the same linear regression coefficients to adjust the raw PGS in GEL samples. For all analyses, residuals were scaled so that the combined set of unrelated control samples from GEL and UKHLS (or GEL only for $PGS_{NDC,DDD}$) had mean = 0 and standard deviation = 1.

To maximise the prediction power across all traits, we constructed composite PGSs combining individual scores except for $PGS_{NonCogEA}$, which we excluded because it was derived from a GWAS-by-subtraction analysis using GWAS summary statistics of educational attainment and cognitive performance, and thus does not provide additional information. We constructed two PGSs, with and without incorporating $PGS_{NDC,DDD}$:

$$PGS_{EA+CP+SCZ} = w_1 \times PGS_{EA} + w_2 \times PGS_{CP} + w_3 \times PGS_{SCZ}$$

$$PGS_{EA+CP+SCZ+NDC} = w_{1'} \times PGS_{EA} + w_{2'} \times PGS_{CP} + w_{3'} \times PGS_{SCZ} + w_{4'} \times PGS_{NDC,DDD}$$

where w_i indicates the weight assigned to the PGS.

We included the composite PGS without $PGS_{NDC,DDD}$ because the latter showed different results in some analyses from other PGS, and combining all four scores could make it challenging to interpret the results. Notably, $PGS_{NDC,DDD}$ was the only PGS that showed evidence of significant over-transmission (**Figure 3A**) and direct genetic effects in the trio model (**Figure 4**). Therefore, we used $PGS_{EA+CP+SCZ}$ in major analyses and $PGS_{EA+CP+SCZ+NDC}$ to try to maximise the power to distinguish patients and unaffected controls.

To avoid overfitting when training the weights to combine individual PGSs, we trained the weights in one cohort (DDD or GEL) using a logistic regression, and calculated the composite $PGS_{EA+CP+SCZ}$ combining scores using these weights in the other cohort, and *vice versa*. In GEL, the composite PGS was calculated as -0.1637 x PGS_{EA} + -0.08650 x PGS_{CP} + 0.1033 x PGS_{SCZ}, and it was scaled so that GEL controls had mean of 0 and variance of 1. In DDD, it was calculated as -0.2250 x PGS_{EA} + -0.05147 x PGS_{CP} + 0.07283 x PGS_{SCZ}, and it was scaled so that UKHLS control individuals had mean of 0 and variance of 1. Note that the constituent PGSs were corrected for genetic PCs before being combined into this composite PGS. We estimated the variance explained by $PGS_{EA+CP+SCZ}$ in DDD compared to UKHLS and in GEL separately (**Supplementary Table 2**). The results of the major analyses using the composite $PGS_{EA+CP+SCZ}$ meta-analysing DDD and GEL using an inverse-variance based method are concordant with those for PGS_{EA} (data not shown).

When calculating the variance explained by $PGS_{EA+CP+SCZ+NDC}$ in GEL, we used five-fold cross-validation, since $PGS_{NDC,DDD}$ was derived from the GWAS in DDD patients and we could not use them to train the weights. More specifically, we randomly split the GEL case-control samples into five equal-sized sets, keeping the same sample prevalence for each. We trained the weights in four sets and estimated the variance explained in the remaining set, then repeated this procedure four times to estimate the average variance explained across all five sets.

Analyses of polygenic scores

PGS in DDD patients with different configurations of affected relatives

Wright *et al.* showed that being the only affected individual in one's family was associated with a higher chance of getting a genetic diagnosis[21]. Moreover, the more affected relatives the patient had, the lower the chance of receiving a diagnosis. We repeated this analysis in DDD patients affected by neurodevelopmental conditions with PGS available and compared the mean PGS of educational attainment (PGS_{EA}) in subgroups of patients with different configurations of affected relatives based on the number of affected parents, siblings, and more distant relatives, as shown in **Extended Data Figure 7A**. We estimated the odds ratio of getting a diagnosis in these subgroups using a multiple logistic regression model following Wright *et al.*:

$$\begin{gathered} Diagnostic\ status \sim configuration\ of\ affected\ relatives\ +\ age\ +\ sex \\ +time\ since\ consent + trio\ status + FROH + number\ of\ DECIPHER\ variants \\ +number\ of\ organ\ systems\ affected + death + neonatal\ ICU\ stay \\ +gestation\ weeks\ + severity\ of\ intellectual\ disability\ or\ developmental\ delay\ (ID/DD) \\ +presence\ of\ seizures + reminiscent\ syndrome + recruiting\ centre + maternal\ diabetes \\ +maternal\ use\ of\ antiepileptic\ drugs + maternal\ history\ of\ pregnancy\ loss \end{gathered}$$

We no longer controlled for ancestry because we calculated PGS in only patients with genetically-inferred GBR ancestry. The reference group for the odds ratios in **Extended Data Figure 7A** is the subset of patients without any affected relatives.

Assessing collinearity in the trio model

We were concerned about the possibility of collinearity between the PGSs in the trio model (a logistic regression; results in **Figure 4**):

$$1_{NDC\ status} \sim \hat{\delta}\ \times\ PGS_{child}\ +\ \hat{\theta}_{m,NT}\ \times\ PGS_{mother}\ +\ \hat{\theta}_{f,NT}\ \times\ PGS_{father}$$

where $1_{NDC\ status}$ is an indicator variable for whether the individual is an NDC case (1) or control (0).

The child's PGS is highly correlated with the parental PGS, and we observed correlation coefficients up to 0.59 (between children and mothers for PGS_{EA}). To assess the severity of collinearity in the trio model, we calculated the variance inflation factor (VIF) for the three scores for the various PGSs examined. As expected, the VIF of the child's PGS (ranging from 2.02 to 2.35; highest VIF observed for PGS_{EA}) is higher than that of the parental PGS (ranging from 1.49 to 1.62), meaning that the variance explained by parental PGS in the child's PGS is higher than that explained in a parent's PGS by other two PGS. However, the highest VIF amongst all five PGSs examined did not exceed the commonly-used cutoff of 5, suggesting that collinearity is not too concerning.

Testing whether prematurity mediates the effects of non-transmitted alleles

To test the hypothesis that the effects of non-transmitted alleles associated with educational attainment and cognition might be mediated by prematurity, we reran the trio model (**Figure 4**) in several ways using a subset of trios for which data on gestational age were available, namely undiagnosed cases from DDD (N=1,521 trios) and control trios from MCS (N=2,451; **Supplementary Figure 5**, **Supplementary Note 7**). We ran the trio model adjusting for

prematurity as a binary covariate (<37 full weeks or ≥37 weeks), in trios excluding probands who were born prematurely (16% of cases and 6% of MCS controls), and in only premature probands. Among the probands who were born prematurely, the severity of prematurity in DDD was higher than MCS, with 5% born extremely prematurely (<28 weeks) in DDD versus 3% in MCS and 15% with a gestational age between 28 to 32 weeks in DDD vs 12% in MCS. To account for this, we also ran the trio model in premature probands controlling for the severity of prematurity as a categorical covariate indicating whether the proband belonged to any of the following three categories: with a gestational age <28 full weeks, 28 weeks to <32 weeks, or 32 weeks to <37 weeks.

Construction and incorporation of weights for the Millennium Cohort Study

The Millennium Cohort Study (MCS) deliberately oversampled minority ethnic and disadvantaged individuals[22], which can lead to biased estimates of mean trait values, including PGSs, within the sample (sampling bias). In addition, nonrandom missingness in each wave of data collection (including the collection of DNA for genotyping) due to biased attrition can also lead to biased estimates of mean trait values (non-response bias). MCS developed sampling weights to adjust for the initial non-random sampling. To correct for non-response bias, we produced non-response weights using inverse probability weighting as previously described[22,23]. First, we conducted a logistic regression on whether a MCS child had genotype data using the following covariates collected at the first study sweep, which had minimal missingness:

has_genotype_data ~ housing_tenure + parental_years_in_education + language_spoken_at_home + study_strata + single_parent_status + breastfeeding_status

The variable study_strata consists of nine categories of individuals from each country in the UK (England, Wales, Scotland, Northern Ireland), each of which was stratified by classification as advantaged or disadvantaged, plus an additional classification of "ethnic" sampling only in England. The variables from which these covariates were taken were as follows: ADROOW00, APLFTE00, ADHLAN00, PTTYPE2, ADHTYS00, ACBFEV00. Individuals with any missingness for these variables were excluded from the weighting procedure. As these variables were collected at the first sweep of the study, missingness was low, with >96% of individuals having complete data for the selected variables.

We fitted this model to predict who was within the sample of unrelated GBR-ancestry individuals with genotype data (N=5,884 of 6,036 children who had no missingness on the variables above), and separately to predict who was within the subset of these that additionally had genotype data on both parents (N=2,445 of 2,498 trio children who had no missingness). In the latter model, we included an additional covariate for whether the child had any genotype data. These models had a Nagelkereke R^2 of 0.51 and 0.42, respectively.

We then extracted the predicted value for each genotyped individual as the probability of being genotyped (or the probability of being in a trio from which all three members were genotyped) and used the inverse of that probability as the non-response weight per individual. Intuitively, those with lower predicted probabilities for having genotype data but nonetheless were genotyped were assigned higher weights. We removed individuals with weights more than three standard deviations above the mean, as these likely represent phenotyping errors driving erroneously low non-response probabilities. This removed fourteen individuals for the weights

in all genotyped individuals and three when restricting to those in trios. To calculate an overall weight taking into account both sampling and non-response bias, we multiplied the sampling weights provided by MCS with the non-response weights we had calculated.

To calculate the mean PGS for the groups shown in **Extended Data Figure 6C**, we fitted a linear regression for the PGS in R (formula: 1 ~ PGS) with no covariates and included the weights using the "weights" argument, such that the intercept for the model returns the weights-adjusted mean for the sample. To calculate the weighted correlation between PGS and rare variant burden score (RVBS) shown in **Supplementary Figure 7**, we fitted a linear regression in R regressing scaled PGS on scaled RVBS (formula: scale(RVBS) ~ scale(PGS)) incorporating the weights similarly, such that the regression coefficient returns the weights-adjusted correlation.

Enrichment of gene sets and pathways

As described in **Supplementary Note 9**, we conducted gene set enrichment analyses to explore mechanistic overlap between the rare and common variants contributing to risk of neurodevelopmental conditions. We used 19,230 autosomal protein-coding genes as the background, downloaded from Ensembl (queried on 3rd May, 2024). We took 1,722 genes that had been prioritised with the DEPCT tool at FDR <5% in the GWAS of educational attainment[18] (which focused on autosomal genes) that were mapped to one of the Ensembl protein-coding genes as "EA genes". We took 788 autosomal DDG2P genes in which at least one monogenic diagnosis (**Methods**) had been found in NDC patients from DDD ("diagnostic DDG2P genes"). We performed Fisher's exact test to assess if EA genes were significantly more likely to be a diagnostic DDG2P gene compared to other protein-coding genes that were not prioritised as an EA gene. There were only two genome-wide significant loci from the NDC GWAS meta-analysis, so we did not prioritise genes using this GWAS.

We next explored whether various gene sets were enriched in EA genes and whether similar or different enrichments were observed for diagnostic DDG2P genes. We firstly took two sets of genes that were preferentially expressed in prenatal or postnatal brain cells identified using weighted gene correlation network analysis by Li *et al*.[24] (Supplementary Table 10 in their paper), resulting in 7,373 and 6,567 genes mapped to Ensembl protein-coding genes, respectively. We also defined 24 gene sets specific to particular cell types in the prenatal brain using the single cell RNA sequence data from Li *et al*.[24], as follows. We focused on Ensembl protein-coding genes and removed genes that were not expressed in any of the 24 prenatal brain cells, following previous studies[25]. We performed $\log_2$ transformation after adding a pseudocount to gene expression values (count per million). For each gene, we calculated an average cell type-specific gene value across cells assigned to a cell type of interest. This value was divided by the average expression of the gene across all 24 prenatal brain cell types. Through this, we selected 1,688 genes per cell type with the top 10% gene expression levels relative to all prenatal brain cells. We applied Fisher's exact test to assess if EA genes and diagnostic DDG2P genes are enriched in the aforementioned gene sets relative to all other genes. We performed two-sided z-score tests to assess whether the degree of enrichment is significantly different between EA genes and diagnostic DDG2P genes. To correct for multiple tests, we applied a Bonferroni correction for 52 tests (26 gene sets * 2 target gene sets from EA GWAS and DDG2P).

In a complementary approach, we used stratified LD score regression[26] to estimate partitioned SNP heritability that was attributable to SNPs in or near (within 100 kb) genes that belong to particular gene sets. The degree of enrichment was calculated as the proportion of heritability explained by SNPs in or near genes of interest divided by the proportion of SNPs mapping to these regions. We applied stratified LD score regression to the GWAS summary statistics for both NDCs and EA to calculate SNP heritability that was attributable to (1) SNPs near diagnostic DDG2P genes, and (2) the aforementioned 24 gene sets specific to certain cell types in prenatal brain. Note that our NDC GWAS proved underpowered to run stratified LD score regression (the heritability z score <7 and $N \times h^2 < 4{,}500$; cutoffs were recommended by the authors[26]), so we focus on results from the EA GWAS.

Supplementary Figures

Figure S1

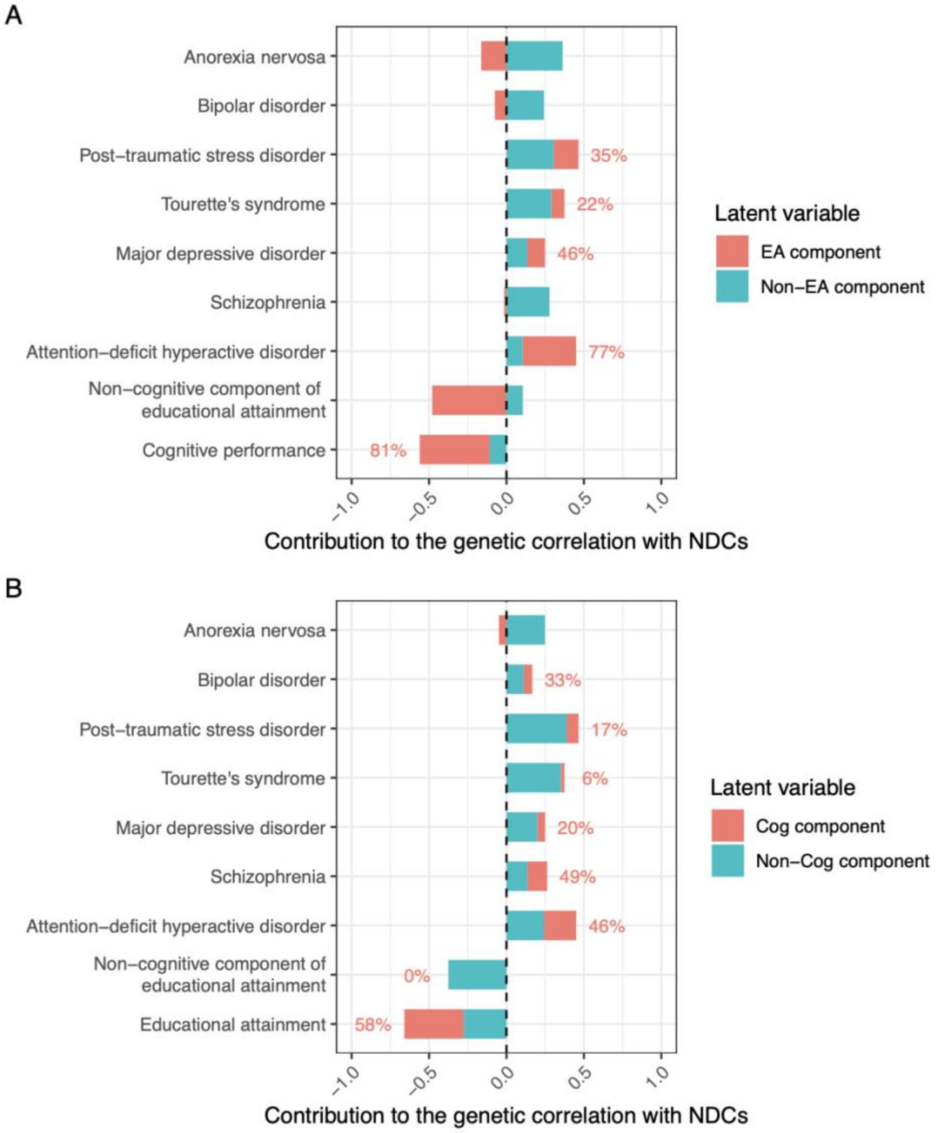

Supplementary Figure 1. Percentage of the genetic correlation between neurodevelopmental conditions (NDCs) and brain-related traits that is explained by the educational attainment (EA) and the non-EA components of neurodevelopmental conditions (**A**) and the cognitive (Cog) and non-cognitive (Non-Cog) components of neurodevelopmental conditions (**B**). The proportions were estimated using the Genomic-SEM decomposition analysis (**Supplementary Methods**). Red indicates the contribution of the EA component in panel A (or Cog in panel B), and green indicates the contribution of the Non-EA component of NDCs (or Non-Cog in panel B). When the two components are in the same direction, we calculated the percentage of the contribution from the red component to r_g, which is annotated beside the bar, and the sum of height of the green and red bars represents the r_g between NDCs and the trait/condition on the y-axis. We did not calculate the percentage when the contribution of the two latent components to the r_g showed opposite directions (e.g. anorexia nervosa), and in this instance, the difference in height between the green and red bars represents the r_g between NDCs and the brain-related trait.

Figure S2

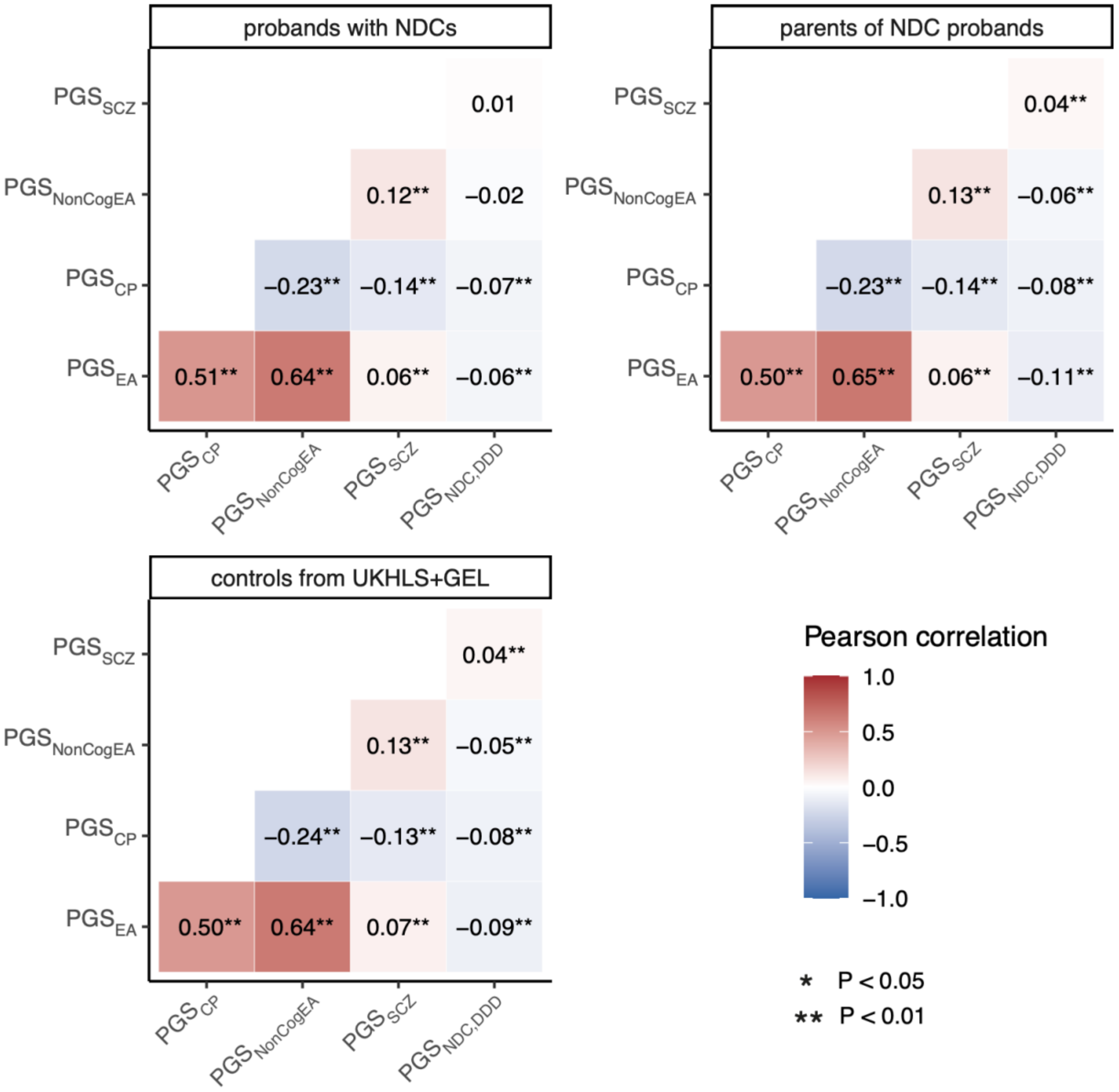

Supplementary Figure 2. Pearson correlations between the five polygenic scores (PGS) used throughout this paper. Correlations were estimated in the following three subgroups: probands with neurodevelopmental conditions (NDCs) regardless of trio status (N=3,618 from GEL and N=6,883 from DDD; N=597 in DDD excluding GWAS samples), parents of probands from 2,174 DDD trios and 2,390 GEL trios, and controls individuals from GEL (N=13,667) and UKHLS (N=9,270). EA: educational attainment; CP: cognitive performance; NonCogEA: the non-cognitive component of EA; SCZ: schizophrenia; NDC,DDD: neurodevelopmental conditions, with the GWAS conducted in DDD versus the UK Household Longitudinal Study, and the polygenic score tested only in samples excluded from the GWAS (GEL and DDD OmniChip). The statistical results are provided in **Supplementary Table 18**.

Figure S3

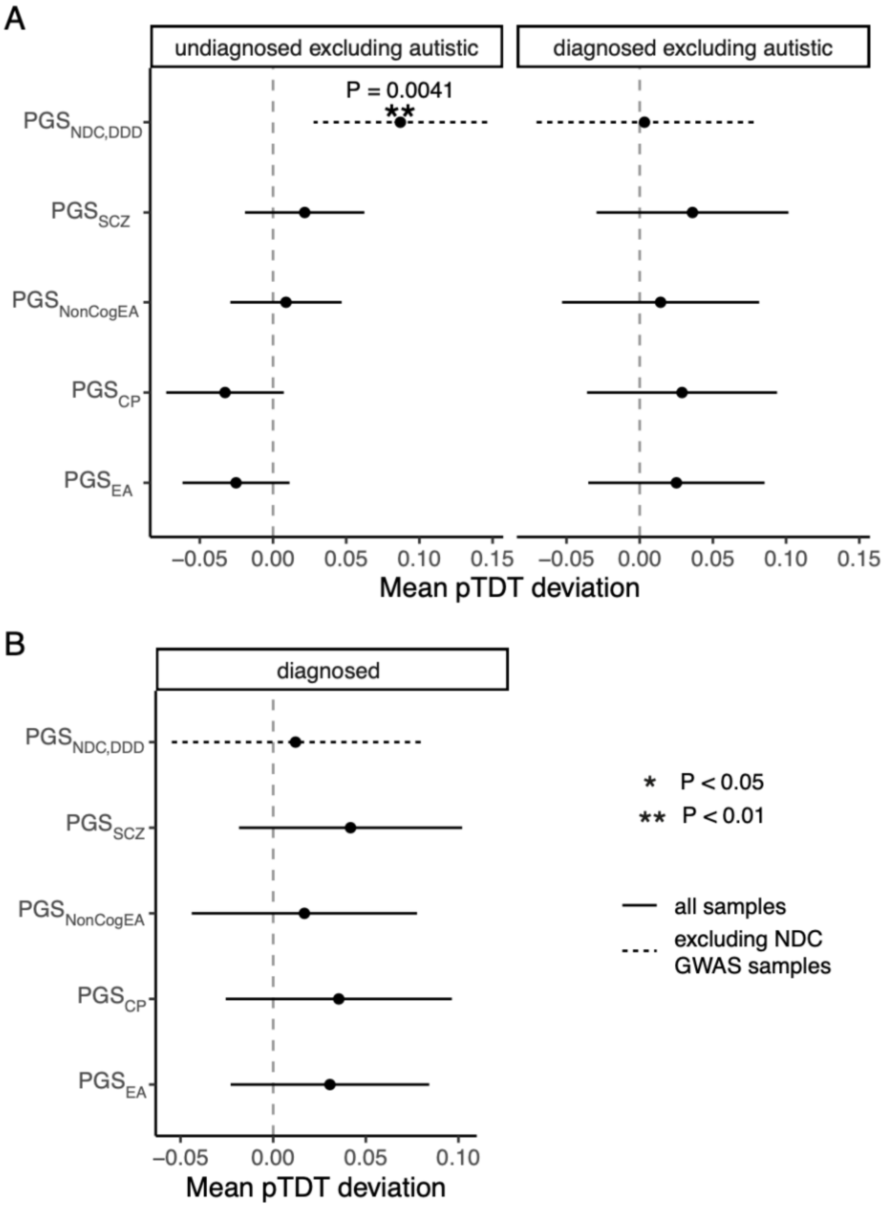

Supplementary Figure 3. Polygenic transmission disequilibrium test (pTDT) results in subgroups. (**A**) pTDT results having excluded autistic probands, in either undiagnosed or diagnosed probands (N=1,298 undiagnosed in DDD, N=192 excluding NDC GWAS samples, N=907 in GEL; N=395 diagnosed in DDD, N=268 excluding NDC GWAS samples, N=389 in GEL). (**B**) pTDT results in probands with a monogenic diagnosis (N=443 in DDD, N=296 excluding GWAS samples; N=507 in GEL). The pTDT results are provided in **Supplementary Table 9.**

Figure S4

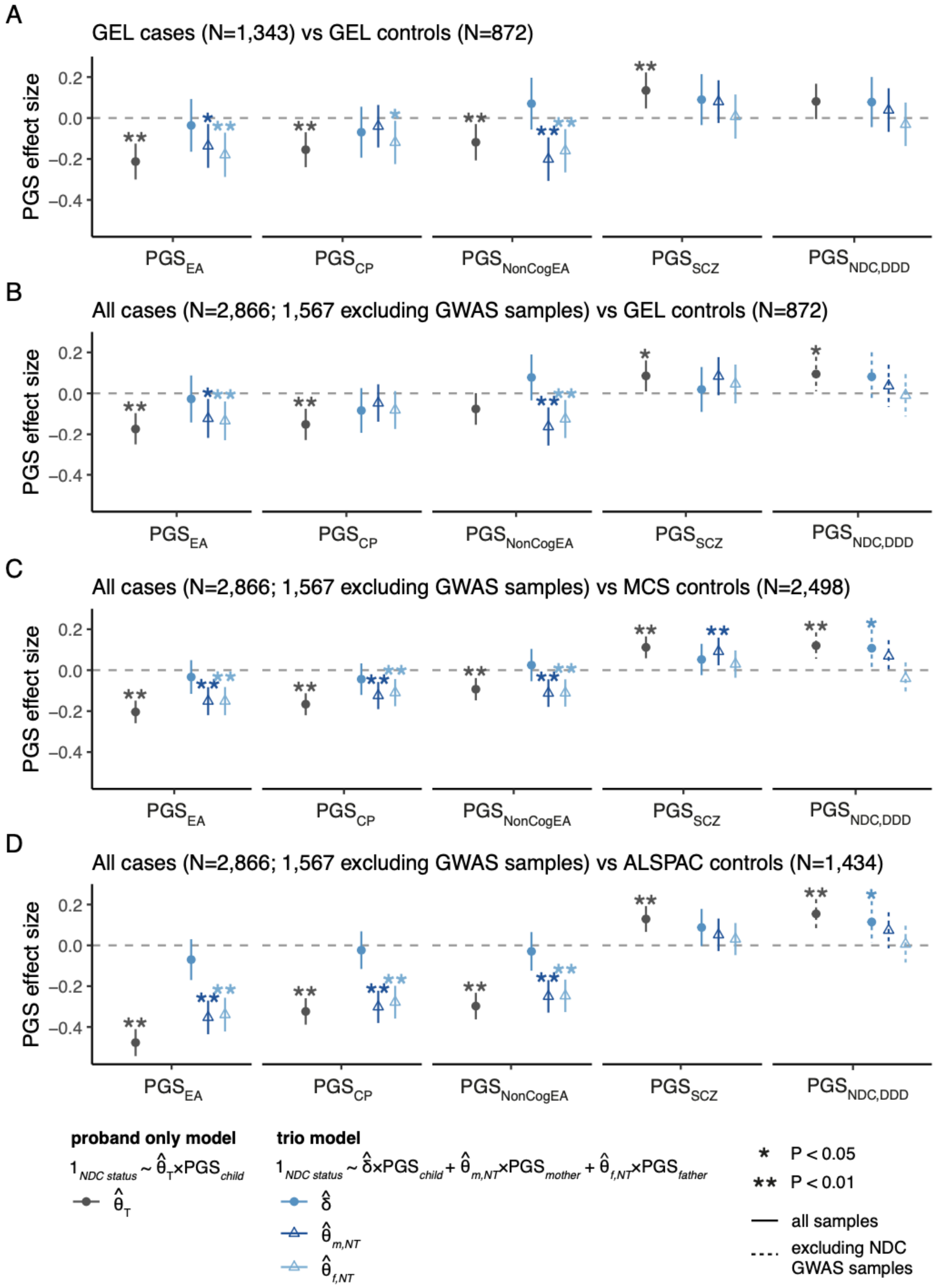

Supplementary Figure 4. Sensitivity analysis to assess non-transmitted coefficients and direct genetic effects of PGS in **A**) only GEL trios, **B**) in all cases with neurodevelopmental conditions versus GEL control trios, **C**) in all cases versus MCS control trios, and **D**) in all cases versus

ALSPAC control trios. All cases with neurodevelopmental conditions are undiagnosed and both parents are unaffected. Y axes show effect sizes of PGSs on case/control status, testing either the child's PGS alone ("proband only"), or while additionally controlling for the parents' PGSs ("trio model") in logistic regression models (**Methods**). Two asterisks indicate significance at p-value < 0.01 (Bonferroni correction for five PGSs) and one asterisk indicates nominally significant at p-value < 0.05. Error bars indicate 95% confidence intervals. The statistical results are provided in **Supplementary Table 10**.

Figure S5

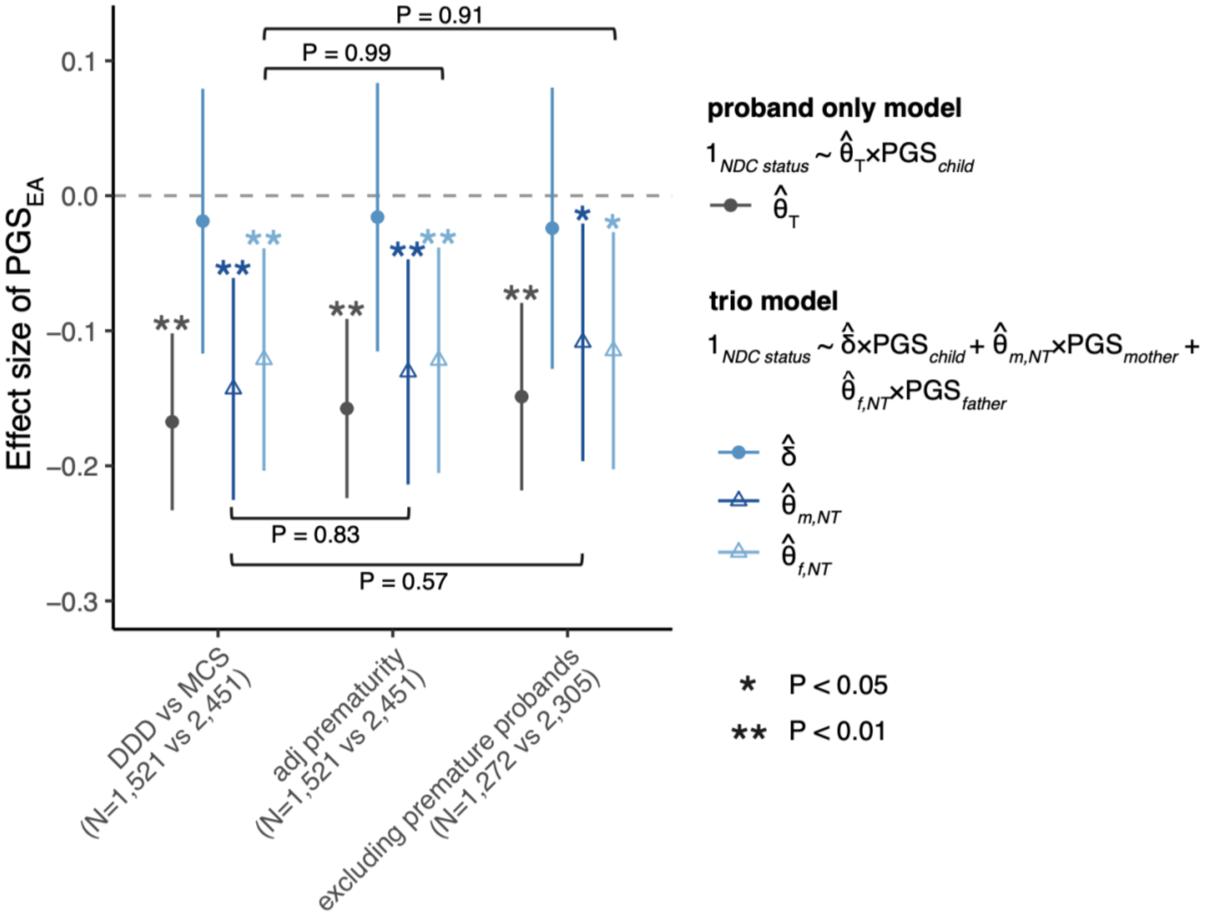

Supplementary Figure 5. Exploring whether prematurity may mediate the association between non-transmitted alleles of PGS_{EA} and risk of neurodevelopmental conditions. Estimates of the effect of PGS_{EA} from the "proband-only" model (grey) or the "trio model" (different shades of blue), as in **Figure 4**, were run on different subsets of probands. Three different logistic regression models were run, plotted in this order: using all DDD and MCS probands either before ("DDD versus MCS") or after controlling for whether or not the proband was born prematurely ("adj prematurity"), or using DDD and MCS probands excluding those who were born prematurely ("excluding premature probands"). Error bars indicate 95% confidence intervals. P-values beside brackets were obtained from two-sided z-score tests assessing whether the difference in maternal or paternal non-transmitted coefficient was significant. The statistical results are provided in **Supplementary Table 10**.

Figure S6

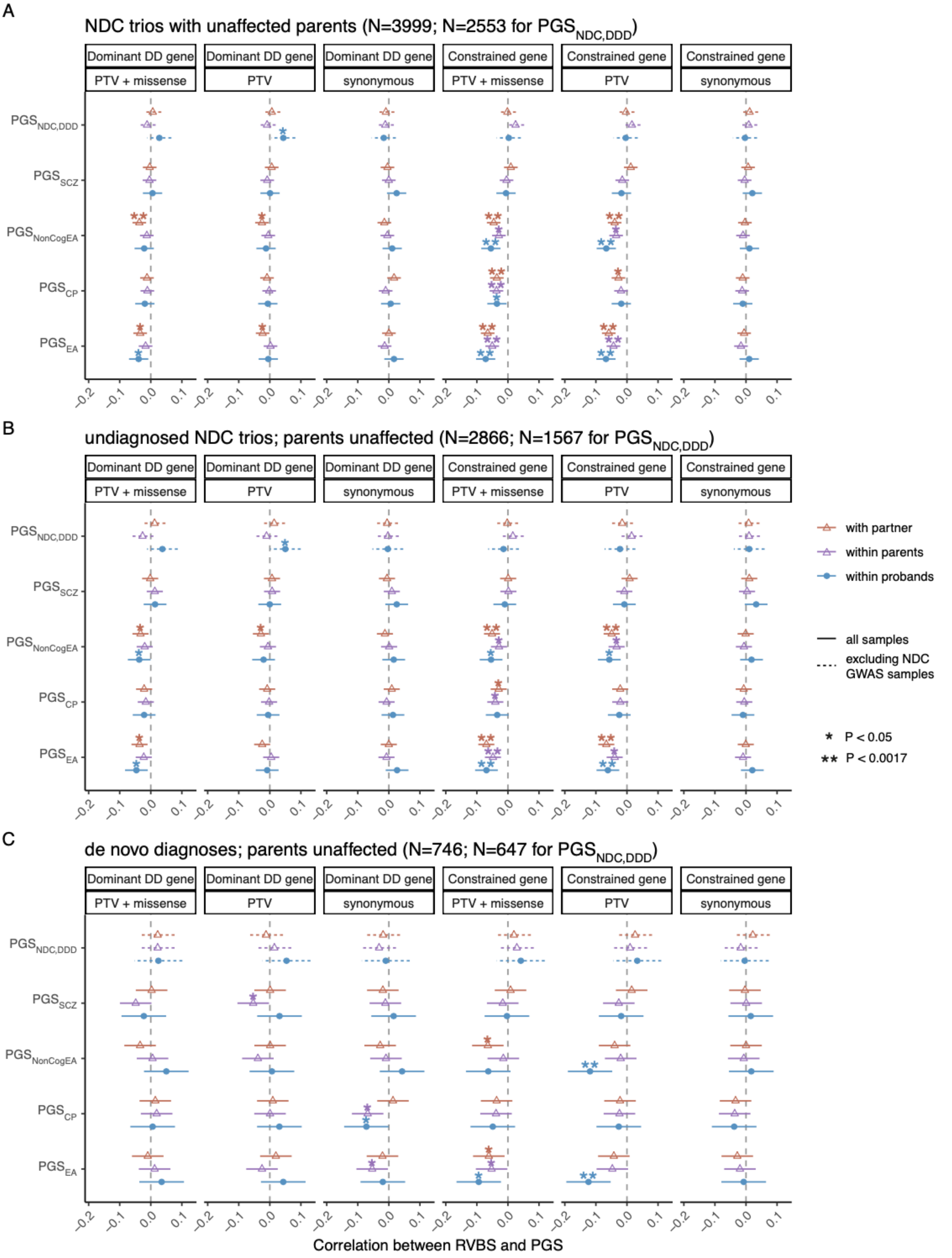
A
NDC trios with unaffected parents (N=3999; N=2553 for PGS_NDC,DDD)
B
undiagnosed NDC trios; parents unaffected (N=2866; N=1567 for PGS_NDC,DDD)
C
de novo diagnoses; parents unaffected (N=746; N=647 for PGS_NDC,DDD)
Dominant DD gene
Constrained gene
PTV + missense
PTV
synonymous
PGS_NDC,DDD
PGS_SCZ
PGS_NonCogEA
PGS_CP
PGS_EA
-0.2
-0.1
0.0
0.1
with partner
within parents
within probands
all samples
excluding NDC GWAS samples
* P < 0.05
** P < 0.0017
Correlation between RVBS and PGS

Supplementary Figure 6. Pearson correlation coefficients between PGSs and the number of inherited rare coding variants defined in various ways within/between different sets of individuals, in different subsets of trios with neurodevelopmental conditions. The correlations within probands with neurodevelopmental conditions whose parents are unaffected are shown in blue (i.e. the child's rare variant burden score, RVBS, with their own PGS), and those within their parents are shown in purple. The cross-parental correlation (i.e. one parent's RVBS with the other parent's PGS) is shown in orange. We conducted the analysis either in all trios with neurodevelopmental conditions in which both parents were unaffected (**A**), in undiagnosed trios with unaffected parents (**B**) or in trios with *de novo* diagnoses and unaffected parents (**C**). RVBSs were calculated using PTVs, PTV and missense variants combined, or synonymous variants in dominant DD genes with a loss-of-function mechanism or in constrained genes. Significant correlations that pass Bonferroni correction for 30 tests (P-value < 0.0017; five PGSs, three variant types, and two gene sets) are indicated by two asterisks, and nominally significant correlations (P-value < 0.05) are indicated by one asterisk. Note that both the rare variant burden scores and PGS have been corrected for 20 genetic principal components. Error bars represent 95% confidence intervals. The statistical results are provided in **Supplementary Table 12**.

Figure S7

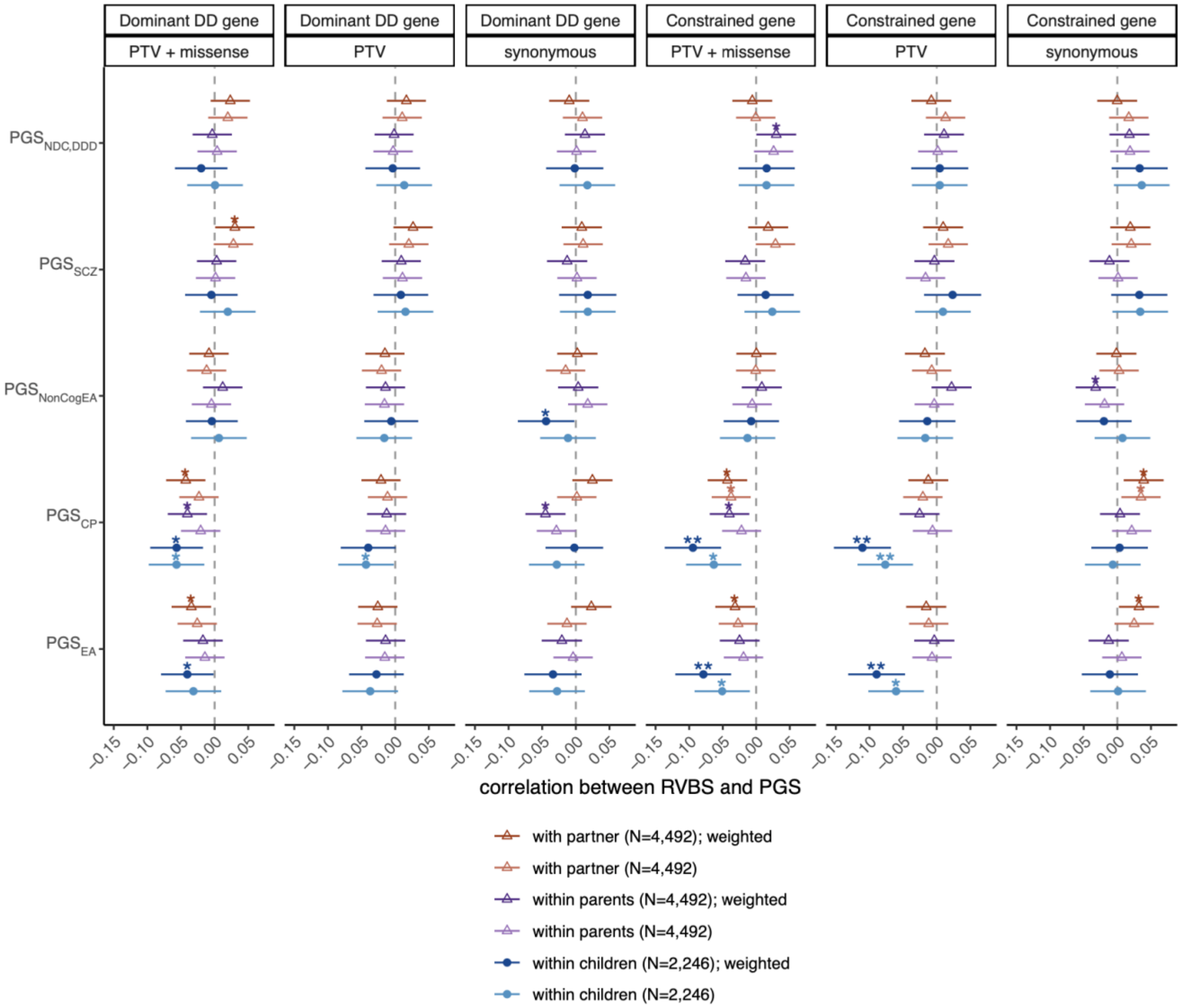

Supplementary Figure 7. Pearson correlation coefficients between PGSs and the number of rare coding variants filtered in various ways within/between different sets of individuals, in control trios (N=2,246) from the Millennium Cohort Study (MCS). The correlations within children are shown in blue (i.e. the child's inherited rare variant burden score, RVBS, with their own PGS), and those within their parents are shown in purple. The cross-parental correlation (i.e. one parent's RVBS with the other parent's PGS) is shown in orange. RVBSs were calculated using PTVs, PTV and missense variants combined, or synonymous variants in dominant DD genes with a loss-of-function mechanism or in constrained genes. The weighted correlations after adjusting for sampling bias and non-response bias (attrition) (**Supplementary Methods**) are shown in darker colours. Significant correlations that pass Bonferroni correction for 30 tests (P-value < 0.0017; five PGSs, three variant types, and two gene sets) are indicated by two asterisks, and nominally significant correlations (p-value<0.05) are indicated by one asterisk. Note that both the rare variant burden scores and PGS have been corrected for 20 genetic principal components. Error bars represent 95% confidence intervals. The statistical results are provided in **Supplementary Table 12**.

Figure S8

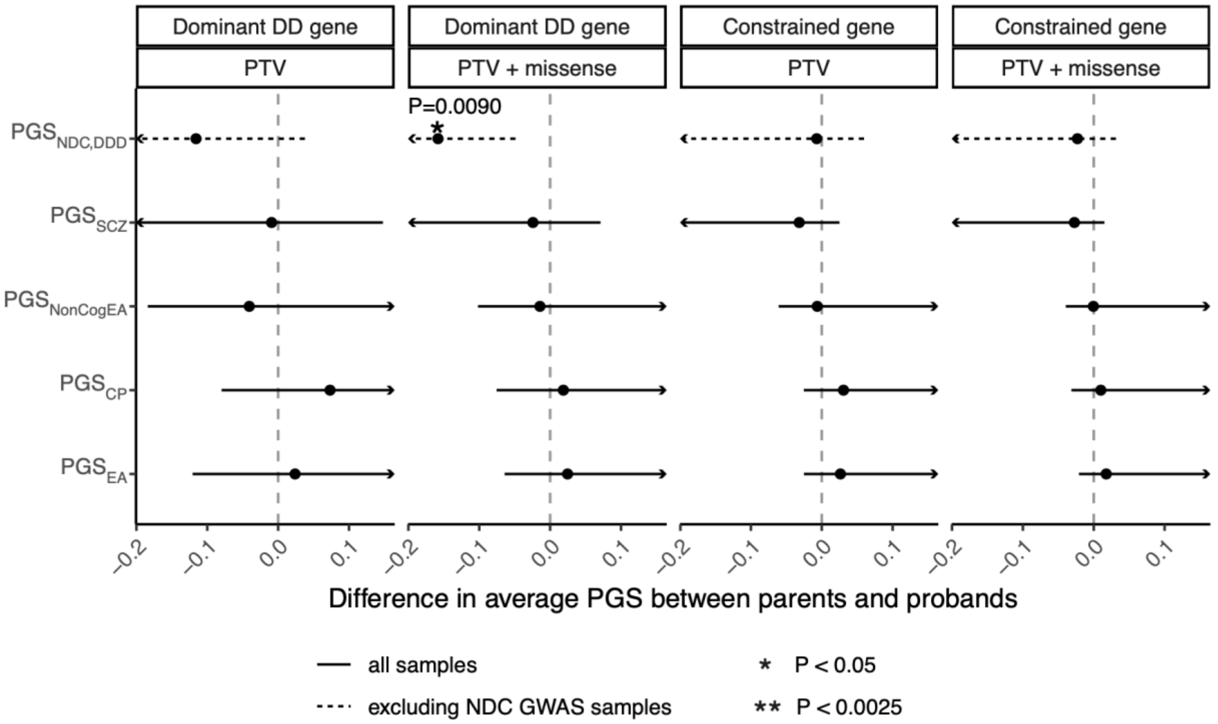

Supplementary Figure 8. Investigation of PGSs modifying penetrance of rare damaging coding variants within families. The plot shows results from one-sided, paired *t*-tests comparing PGSs between unaffected parents transmitting damaging rare variants and their undiagnosed children from 2,866 trios from DDD and GEL (or 1,567 excluding NDC GWAS samples). A positive difference indicates that the unaffected parents have higher PGS than the children. One asterisk indicates a nominally significant difference; none of the differences passed Bonferroni correction for 20 tests (five PGSs, two variant types, and two gene sets). Error bars indicate 95% confidence intervals (CIs). The upper bound of CI is shown for PGS_{SCZ} and $PGS_{NDC,DDD}$, for which an upper-tailed test was used and the lower bound is -infinity. The lower bound of CI is shown for PGS_{EA}, PGS_{CP}, and $PGS_{NonCogEA}$, for which a lower-tailed test was used, and the upper bound is +infinity. The statistical results are provided in **Supplementary Table 19.**

Figure S9

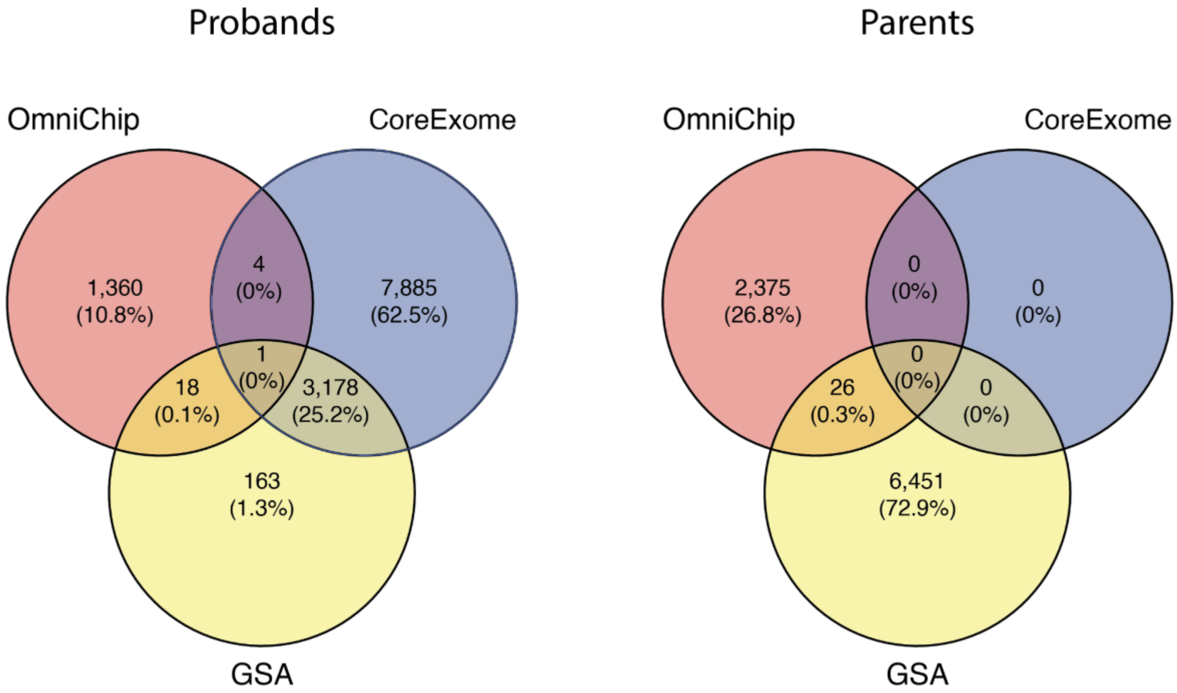

Supplementary Figure 9. Venn Diagrams of DDD samples by genotyping array. Overlapping proband samples (left) and overlapping parent samples (right) across three genotype array chips used in the DDD cohort.

Figure S10

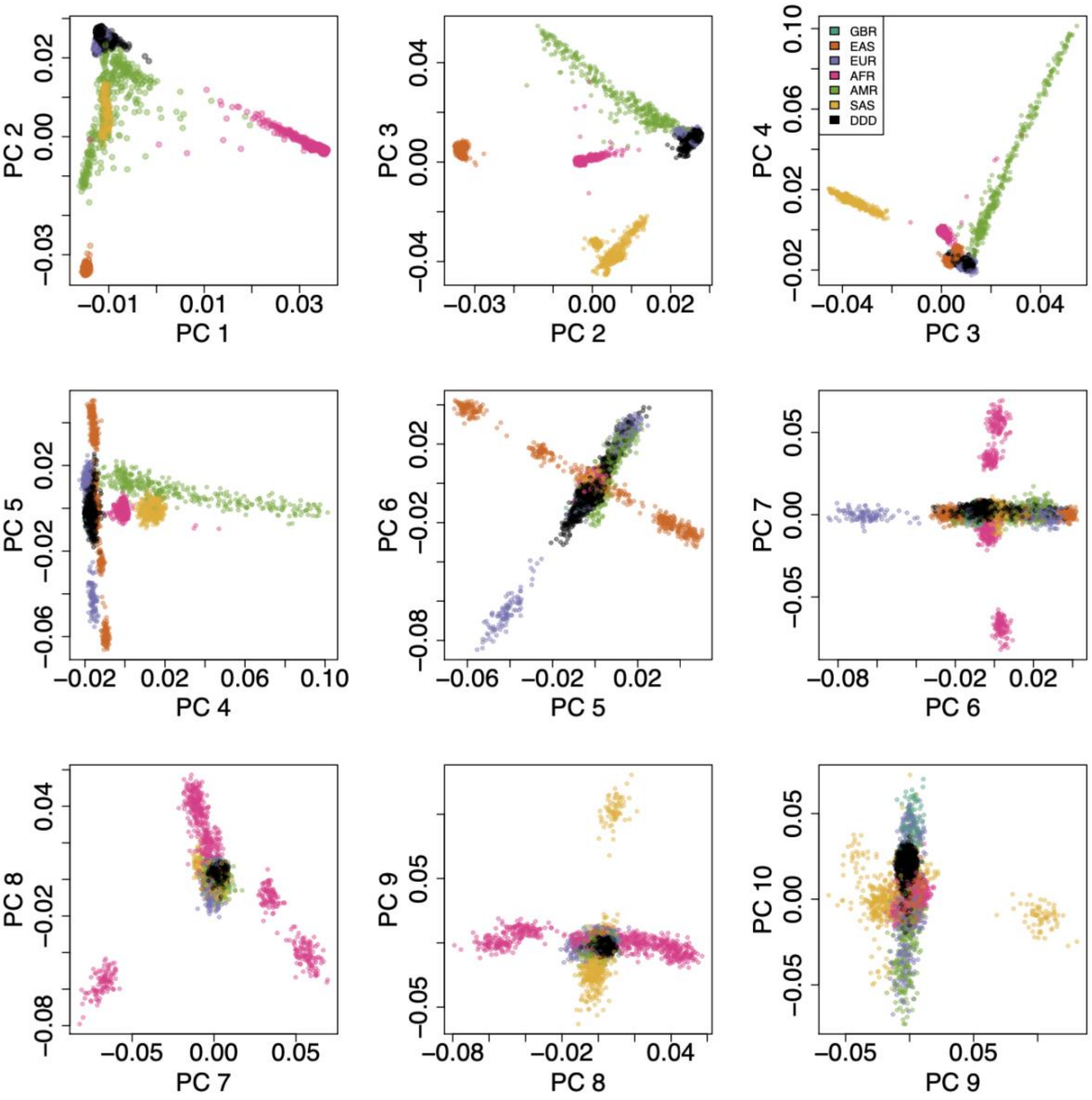

Supplementary Figure 10. Principal components (PCs) of DDD Global Screening Array samples (N = 9,572) and 1,000 Genomes phase 3 samples (N=2,548). DDD individuals are in black, coloured by superpopulation, with the exception of GBR-ancestry individuals. GBR: Great British, EAS: East Asian, EUR: European, AFR: African, AMR: Ad Mixed American, SAS: South Asian.

Figure S11

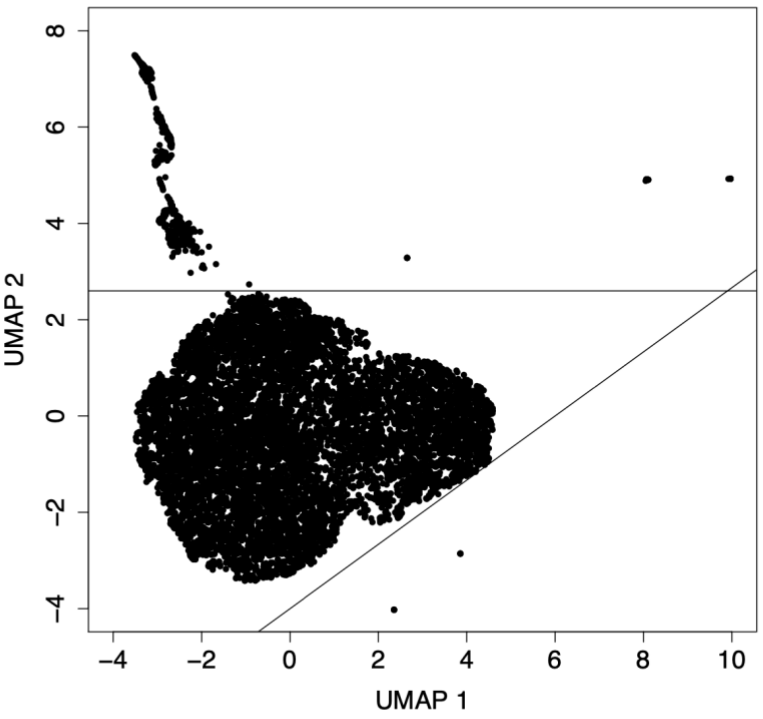

Supplementary Figure 11. UMAP using the first ten principal components from a principal component analysis (PCA) within loosely European-ancestry DDD samples on the Global Screening Array (N=9,534). Black lines indicate the cut-offs chosen to delineate the homogeneous European-ancestry group shown in the bottom left area of the plot, which was taken forward for analysis (N=8,489).

Figure S12

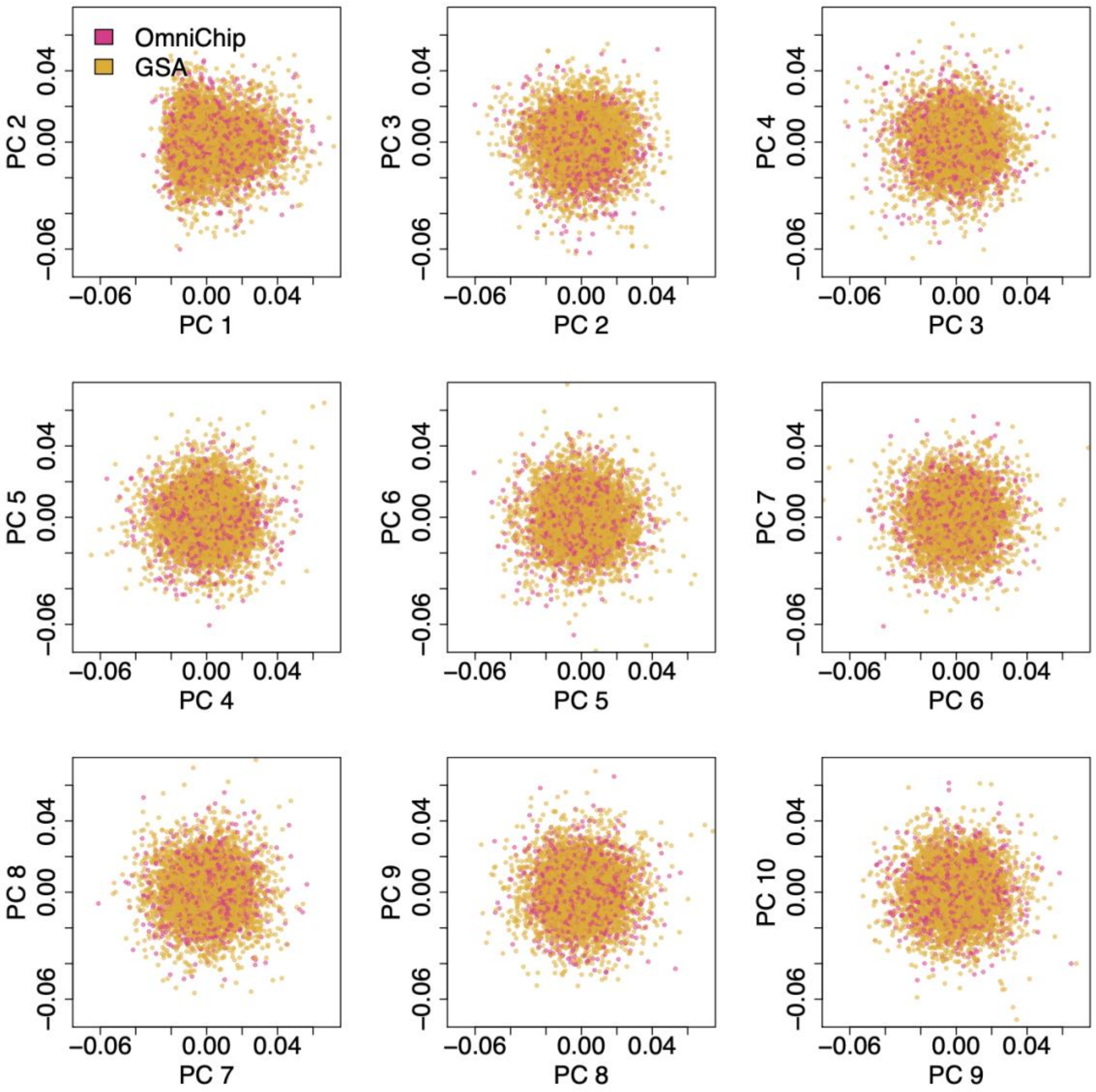

Supplementary Figure 12. Principal components (PCs) of European-ancestry participants in DDD genotyped on the Global Screening Array and GBR-ancestry individuals genotyped on OmniExpress chip from Niemi *et al*.[1].

Figure S13

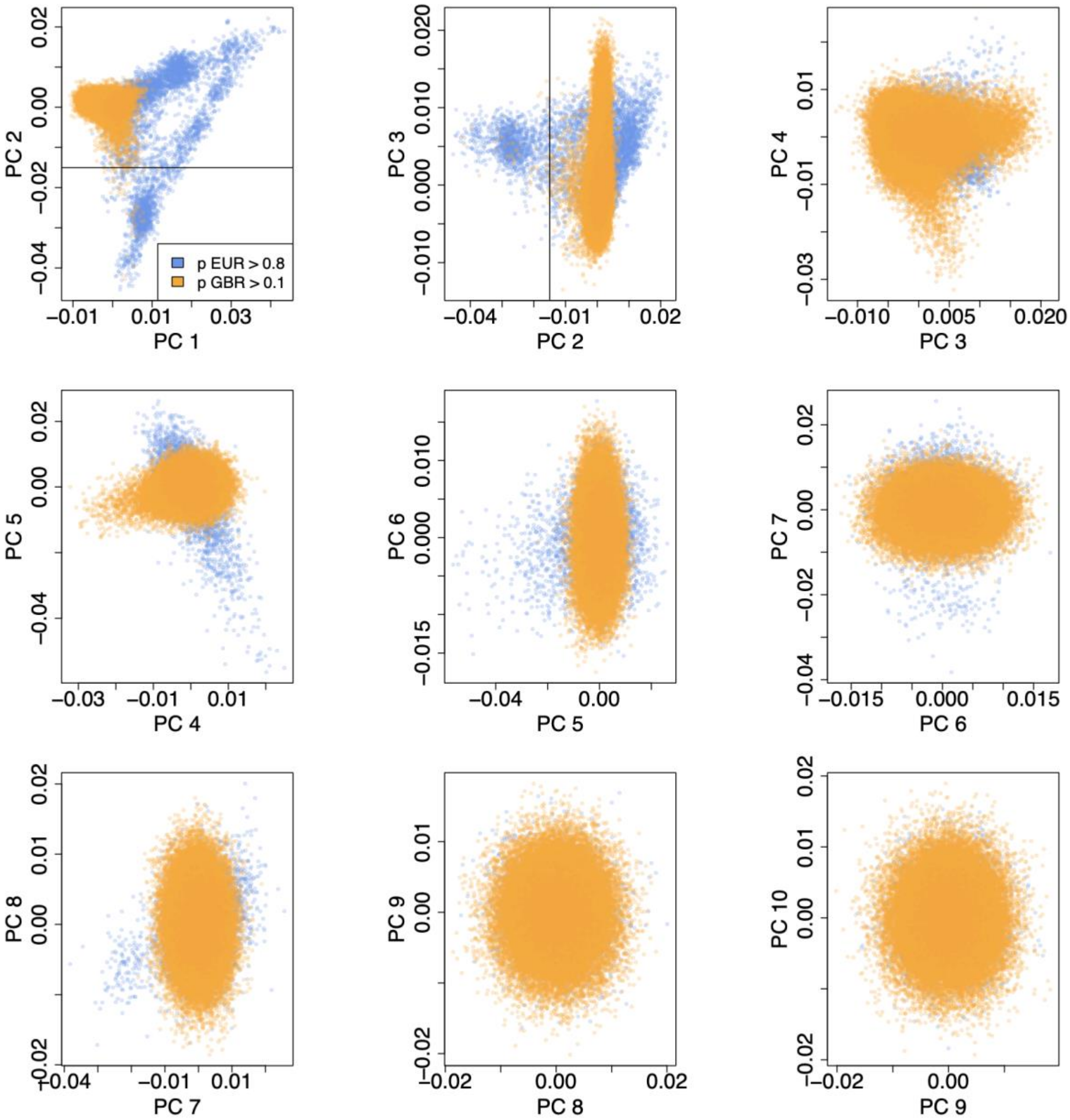

Supplementary Figure 13. Principal components (PCs) of GEL individuals with predicted European ancestry (N= 62,366). All individuals plotted here had a probability of being in the 1,000 Genomes EUR super population > 0.8 (according to a random forest model), and a subset of those with probability of being in the 1,000 Genomes GBR subpopulation > 0.1 (according to a random forest model) are in orange. The black line on PC 2 indicates the PC2 cut-off for individuals selected as GBR ancestry.

Figure S14

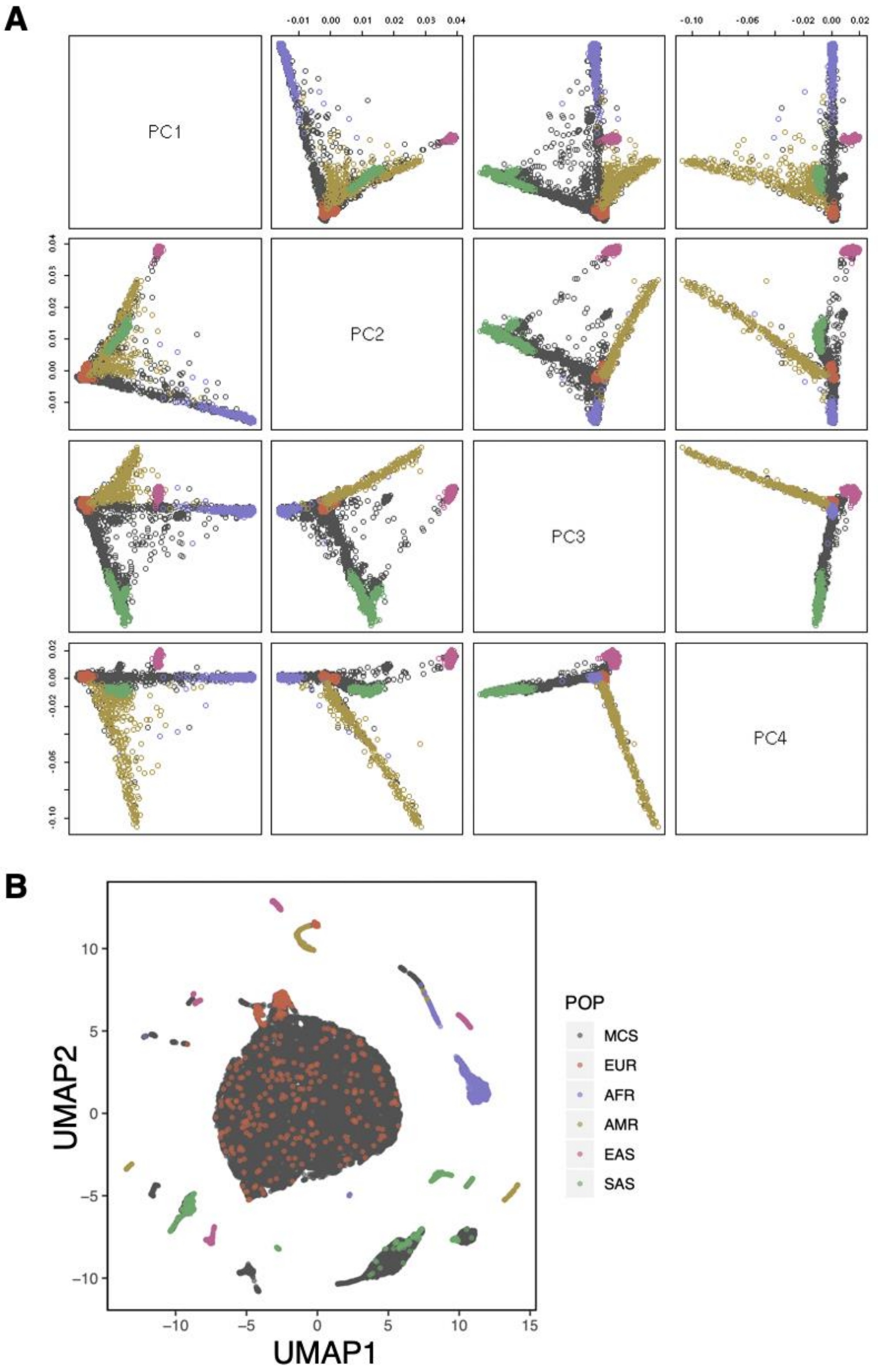

Supplementary Figure 14. Identifying European-ancestry individuals in MCS. **A**) Principal components (PCs) calculated in 1,000 Genomes phase 3 individuals, with MCS individuals shown in black projected to the same PC space. Colours indicate continental-level populations from the 1,000 Genomes project: European (EUR), African (AFR), Ad Mixed American (AMR), East Asian (EAS) and South Asian (SAS). **B**) UMAP using the first four PCs.

Figure S15

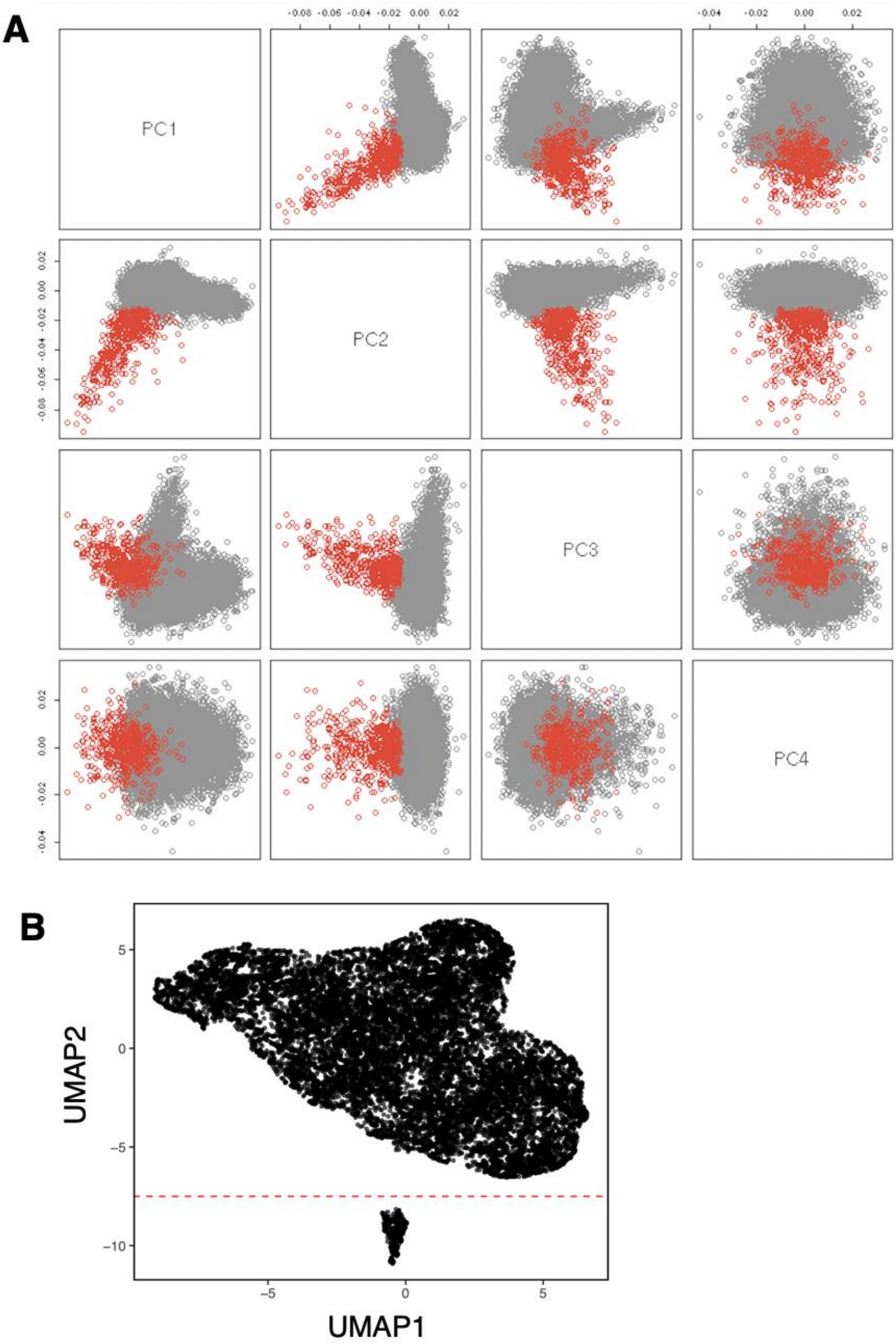

Supplementary Figure 15. Identifying a homogeneous subgroup of European-ancestry individuals in MCS. **A**) Principal components (PCs) of 17,599 MCS samples who were reported to have White ethnicity clustered together with non-Finnish European samples from the 1,000 Genomes project. Red indicates outlier samples that were removed based on being below the line in panel (B). **B**) UMAP using the first four PCs.

Figure S16

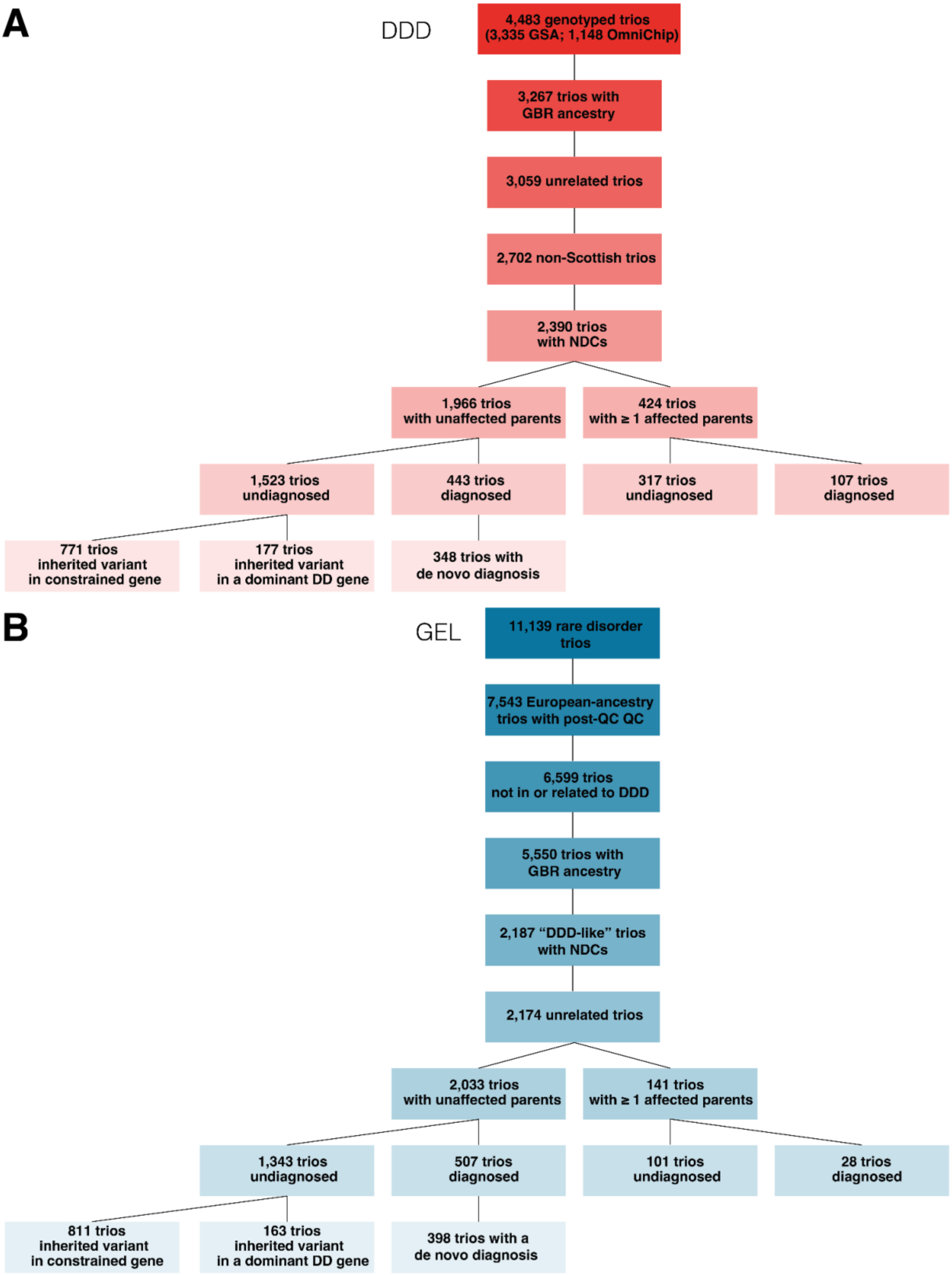

Supplementary Figure 16. Flow diagram of trio filtering in **A**) DDD and **B**) GEL.

Figure S17

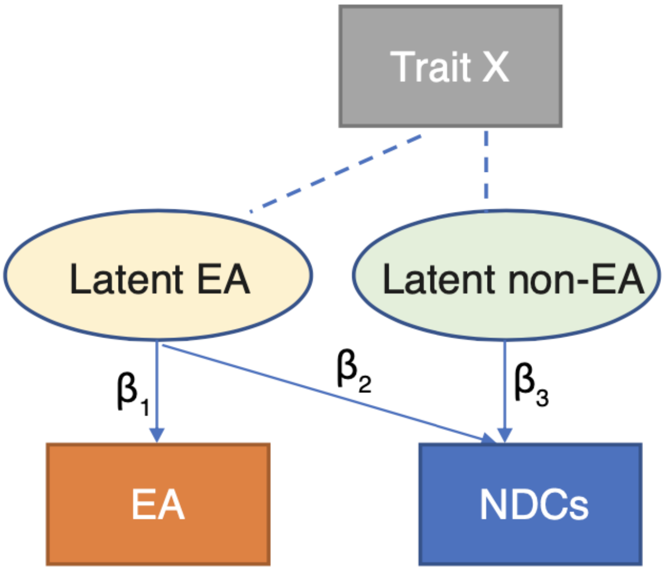

Supplementary Figure 17. GWAS-by-subtraction GenomicSEM model to estimate genetic correlation without SNP effects, modified from Figure 1 in [14]. Educational attainment (EA) and neurodevelopmental conditions (NDCs) are observed variables for which GWAS summary statistics are available. The model assumes two latent variables that are unobserved. The variances of the latent variables are fixed to 1, and covariance between them is fixed to 0. The latent EA variable influences both EA (effect size $\beta_1 = 0.335$) and NDCs (effect size $\beta_2 = -0.126$), and the latent non-EA variable influences only NDCs (effect size $\beta_3 = 0.145$). The latent EA variable explains 43% of the SNP heritability of NDCs and the non-EA variable explains 57% of the heritability.

Figure S18

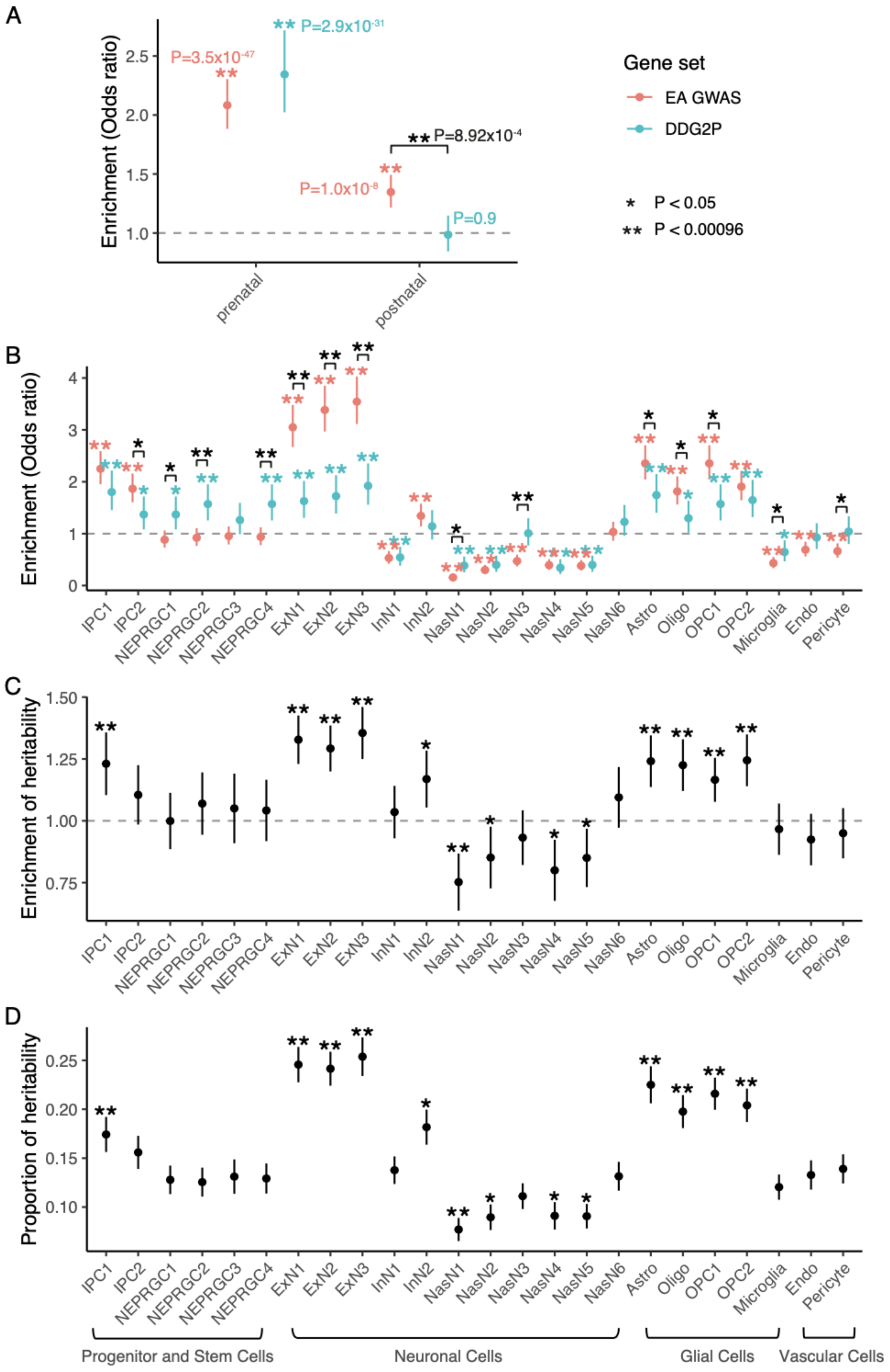
A
Enrichment (Odds ratio)
P=3.5x10-47
P=2.9x10-31
P=8.92x10-4
P=1.0x10-8
P=0.9
prenatal
postnatal
Gene set
EA GWAS
DDG2P
* P < 0.05
** P < 0.00096
B
Enrichment (Odds ratio)
C
Enrichment of heritability
D
Proportion of heritability
IPC1 IPC2 NEPRGC1 NEPRGC2 NEPRGC3 NEPRGC4 ExN1 ExN2 ExN3 InN1 InN2 NasN1 NasN2 NasN3 NasN4 NasN5 NasN6 Astro Oligo OPC1 OPC2 Microglia Endo Pericyte
Progenitor and Stem Cells
Neuronal Cells
Glial Cells
Vascular Cells

Supplementary Figure 18. Enrichment of NDC risk genes implicated via common versus rare variants in brain cell types. We focused on 19,130 autosomal protein coding genes. To represent the genes implicated via common variants, we used EA GWAS genes (N=1,722) prioritised by Lee *et al.* 2018. To represent the genes implicated via rare variants, we took 788 DDG2P genes in which a diagnosis has been found for any DDD proband with an NDC. We used Fisher's exact tests to estimate enrichment (odds ratio) in panels **A** and **B**. (**A**) shows enrichment of genes that are expressed in prenatal or postnatal brain tissues according to Li *et al*.[24] compared to all other autosomal genes. (**B**) shows enrichment of the indicated gene set within genes that show particularly high expression in the indicated cell types relative to other cell types in the prenatal brain. We used single cell RNA sequence data of prenatal brain samples from Li *et al.* to define genes showing particularly high expression in the given cell type (**Supplementary Methods**), as shown on the x-axis. Significant enrichment or depletion relative to all other genes is highlighted by colored asterisks. Black asterisks indicate cell types that showed stronger enrichment in DDG2P genes than EA GWAS genes, or *vice versa*. This was obtained by comparing enrichment estimates using two-sided z-score tests. (**C**) shows enrichment of heritability of educational attainment attributable to SNPs in or near genes that show particularly high expression in the indicated cell types relative to other cell types in the prenatal brain. We applied stratified LD score regression[26] to GWAS summary statistics of educational attainment. Enrichment was estimated as the proportion of heritability explained by SNPs in or near prenatal brain cell type-enriched genes, which is shown in (**D**) divided by the proportion of SNPs mapping to these regions. Error bars indicate 95% confidence intervals. Significant enrichment or significantly different enrichment that passed Bonferroni correction of 52 tests (26 gene sets and 2 target gene lists) is indicated by two asterisks, and nominally significant evidence is indicated by one asterisk. Enrichment statistics in panel (**B**) are provided in **Supplementary Table 20**. Results from stratified LD score regression shown in panels (**C**) and (**D**) are provided in **Supplementary Table 21**. IPC: intermediate progenitor cells; NEPRGC: neural epithelial progenitor/radial glial lineage; ExN: excitatory neurons; InN: interneurons; NasN: nascent neurons; Astro: astroglial lineage; Oligo: oligodendrocytes; OPC: oligodendrocyte progenitor cells; Endo: endothelial cells.

Supplementary Notes

Supplementary Note 1: Lay summary and Frequently Asked Questions

This section was written primarily by Emilie Wigdor, Patrick Campbell and Hilary Martin, with input from other authors of the paper (particularly Elizabeth Radford and Helen Firth) as well as participant representatives from the 100,000 Genomes project (Jillian Hastings-Ward, Hannah Podd and Hannah Humphrey) and the patient organization Unique.

Note to readers: Part 1 provides a brief summary of the results in simple terms. It is available both in an "Easy Read" version and a slightly more technical version. Part 2 (Frequently Asked Questions) provides more detailed information about the paper in lay language, and is aimed at science journalists or people with a similar level of understanding about this topic.

If you are a parent or relative of a child with a neurodevelopmental condition and want to know what this study means for your child and your family, please see question 15.

Part 1: Lay summary

EasyRead version of the summary

Our DNA acts as an instruction book for how to build our bodies. We all have similar DNA but there are also differences in the DNA between us. These are like different spellings in the instruction book that make the recipes for our body unique. Some of these differences in the DNA are rare and found only in a small number of people's DNA. Others are common and found in many people's DNA.

Rare changes in our DNA can change how our brains develop, and cause rare brain conditions in children. These rare brain conditions often cause learning difficulties. Projects like the 100,000 Genomes Project try to find these rare DNA changes and tell families about them. Common DNA differences can also affect the chance of having these conditions and less is known about these.

We are a group of scientists and doctors interested in DNA differences in people with rare brain conditions. In a recent study, we looked at data from thousands of people with rare brain conditions and their parents. We used information from two projects in the UK: the Deciphering Developmental Disorders study and the 100,000 Genomes Project. We also studied data from thousands of people without rare brain conditions. We wanted to understand how common DNA differences contribute to these conditions.

We found that certain common DNA differences are more common in people with rare brain conditions. These same common DNA differences are similar to those that increase the chance of mental health issues like depression. (See here if you are worried about what this means for you or your child). We also found that these same DNA differences are more common in people who have spent less time in education and get lower scores on IQ tests.

Overall, common DNA differences have only a small effect on the chance of having a rare brain condition. But they might help explain why some people have rare brain conditions, especially if they don't have a rare DNA change. We found that these common DNA differences in parents might affect how their child's brain develops, even if the child doesn't directly inherit them. But there might be other reasons for our findings. More research is needed to understand them.

Overall, we found that common DNA differences play a small role in rare brain conditions. So, doctors probably won't use them to help patients understand the cause of their rare brain conditions any time soon. This study helps us understand how DNA and the environment work together to cause rare brain conditions. In the future, this might help families and doctors better understand, diagnose, and treat these conditions.

Lay summary in slightly more technical language

Rare neurodevelopmental conditions affect the growth and development of the brain in childhood. They often lead to learning difficulties and/or seizures. They are often caused by a single rare genetic change. Studies such as the 100,000 Genomes Project and the Deciphering Developmental Disorders study try to identify these rare genetic changes causing patients' conditions ("genetic diagnoses") and report them back to families. Many families find it helpful if they understand the reason why their child has additional challenges and it can also be important for their healthcare. So, it is important to do research to better understand the many different factors that contribute to neurodevelopmental conditions. Genetic differences that are **common** in the general population are known to have small effects on the chance of developing a neurodevelopmental condition. Currently, genetic diagnosis or clinical care would not be based on these common genetic differences.

In this study, we (a group of scientists and doctors) analyzed data from more than 11,500 people with a neurodevelopmental condition and 9,100 of their parents. The data were collected by two projects on rare conditions in the UK: the Deciphering Developmental Disorders (DDD) study and the 100,000 Genomes Project. We also looked at data from 26,800 people without neurodevelopmental conditions. Our goal was to better understand how common genetic differences contribute to these conditions.

We found that the common genetic differences that contribute to these rare, early-onset neurodevelopmental conditions overlap with those that increase the chance of developing later-onset mental health conditions (e.g. ADHD). (See here if you are worried about what this means for you or your child). They also overlap with the common genetic differences that are more likely to be found in people with fewer years in formal education and who get lower scores in tests of mental processes including memory and problem-solving abilities ("cognitive performance"). However, importantly, common genetic differences had only a small impact overall on the chance of someone developing a neurodevelopmental condition.

We found that common genetic differences can help to explain why some people have a neurodevelopmental condition - especially if they have not got a rare genetic diagnosis. We also learned more about how common genetic differences that affect the number of years people spend in education affect the chance of having a neurodevelopmental condition. In particular, our findings suggest that these common genetic differences, when present in the parents, may affect their

child’s neurodevelopmental condition, even if the child does not inherit those genetic differences directly. However, alternative technical explanations may instead be driving the findings. More work is needed to understand them.

There are limitations to this work. We showed that the role of common genetic differences in neurodevelopmental conditions is small. Because of this, they are unlikely to be used by doctors to diagnose or help patients in the near future. Nevertheless, this study brings us closer to fully understanding how different genetic and environmental factors may work together to cause neurodevelopmental conditions. In the longer term, these findings may help families and doctors to better understand, diagnose and manage these conditions.

Part 2: Some questions and answers about the paper

Section 1: Introduction to neurodevelopmental conditions and their causes

By “neurodevelopmental conditions”, we mean conditions that are first noticed during childhood and that affect the growth and development of the brain. They often cause intellectual disability and delays in achieving developmental milestones. Question 20 gives more information about neurodevelopmental conditions.

1. What most often causes rare neurodevelopmental conditions?

Rare neurodevelopmental conditions are most often due to genetic changes. In a large percentage of people with neurodevelopmental conditions (probably at least 40%), their condition is due to a DNA change (variant) that happened in the child with the condition and is not present in the parents. These are called *de novo* variants. If they occur in one of several hundred genes, they can cause a neurodevelopmental condition. However, neurodevelopmental conditions can also be caused by rare inherited variants in a gene (by “rare”, we mean variants typically seen in <1% of people (one in a hundred)). More information on how this can occur can be found here. If we can find the single rare variant (or sometimes, pair of variants) causing an individual’s neurodevelopmental condition, this is referred to as a “genetic diagnosis”.

2. Does everyone who has a rare variant have a rare neurodevelopmental condition?

No. We all have millions of rare variants in our DNA, and most of these don’t cause neurodevelopmental conditions or other conditions. These rare variants are part of the reason we are different from one another (e.g. in height and hair colour). However, it is sometimes possible to have a rare genetic change of the type that usually causes neurodevelopmental conditions but to not actually have one of these conditions. When this happens, we describe the genetic variants as having *incomplete penetrance*. This can happen because the impact of these specific rare genetic changes is influenced by other genetic differences in our DNA, as well as by our environment and random chance.

3. What role does the environment play in neurodevelopmental conditions?

There are known environmental exposures that cause or contribute to the likelihood of developing a neurodevelopmental condition, without causing genetic differences. These

typically occur during development when the child is still in the womb, or shortly after birth. One of the most common environmental influences that can cause neurodevelopmental conditions is being born prematurely. Unfortunately, we often do not have a good understanding of the exact causes of premature birth, and usually nothing can be done to prevent it. Exposure to certain medications or substances (e.g. certain drugs, alcohol) and viruses (e.g. Rubella virus) whilst in the womb can also cause neurodevelopmental conditions. Again, often nothing can be done to avoid these exposures.

Exposure to each of these environmental factors does not always cause neurodevelopmental conditions. **In the case of a child with a neurodevelopmental condition, it is important to have an assessment with a specialist medical professional, including a thorough medical history and examination, before any link between an individual neurodevelopmental condition and an environmental factor is made.**

See here if you have a child with a neurodevelopmental condition and are concerned about the possible role of environmental factors such as prematurity, medications, smoking or alcohol.

4. What is meant by the "genetic architecture" of neurodevelopmental conditions?

This paper is broadly about an aspect of what is called the "genetic architecture" of neurodevelopmental conditions. When we talk about "genetic architecture", we mean understanding how many variants influence the chance of developing a condition, how big their individual effects are, how common they are in the population, and where in the DNA they lie (e.g. in genes or outside genes).

The picture below illustrates a model for some of the different ways it is thought neurodevelopmental conditions may arise. This picture focuses on genetic causes, since this section is about genetic architecture, but as noted in question 3, environmental causes play a role in some neurodevelopmental conditions.

Figure S19

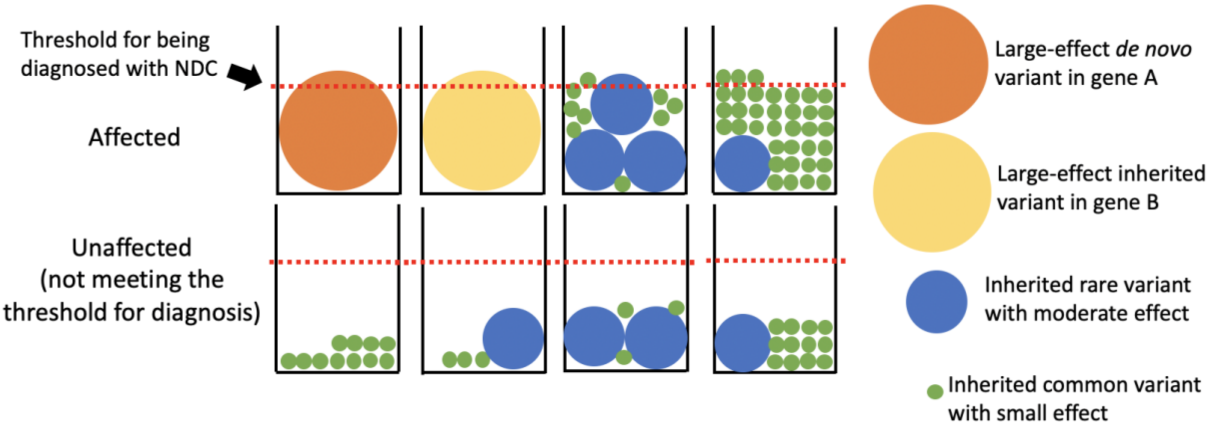

Supplementary Figure 19. Illustration of the "liability threshold model" for neurodevelopmental conditions. *In this picture, each jar represents a hypothetical example of one person. Each person carries one or more genetic variants (represented by the coloured circles) which contribute to their chance of developing a neurodevelopmental condition. The size of the circles*

represents the amount by which each variant influences this chance. The people in whom the circles cross the red line develop a neurodevelopmental condition; this line represents something called the "liability threshold" (see question 23 if you want more information on this). The big orange and yellow circles represent de novo and rare inherited variants that have large enough effects on their own to cause a neurodevelopmental condition (as mentioned in question 1 above). The middle-sized blue circles represent incompletely penetrant rare variants, which have moderate effects on the chance of developing a neurodevelopmental condition (as mentioned in question 2). The small green circles represent inherited common variants (seen in e.g. >1% of people in the population) which have individually very small effects on the chance of developing a neurodevelopmental condition, but which may collectively have an important effect if an individual has enough of them (e.g. in the fourth affected individual). The unaffected individuals in the bottom row have some inherited rare variants and/or some inherited common variants which contribute to the chance of developing a neurodevelopmental condition; however, they don't have enough of them to cross the red line, so they haven't developed a neurodevelopmental condition.

To describe "genetic architecture" another way, we want to understand what percentage of affected individuals have neurodevelopmental conditions due to an orange circle, a yellow circle, or some combination of blue and green circles. Some genetic variants are not sufficient to cause a neurodevelopmental condition on their own (the blue and green circles) and are also found in unaffected individuals. Because of this, when describing genetic architecture, we often talk about the percentage of *variation* in chance of developing a condition due to different genetic mechanisms (e.g. all common variants), rather than simply the percentage of people whose neurodevelopmental condition is "explained" by a variant in a single gene. To fully understand genetic architecture, we also need to understand how different types of genetic variants act together to influence the chance of developing a given condition or to influence variability in clinical features (e.g. severity of seizures).

5. Why is it important to study the genetic architecture of neurodevelopmental conditions? Shouldn't we just focus on finding genetic diagnoses?

Finding genetic diagnoses for patients (i.e. large-effect genetic variants that fully or largely explain their condition - see question 1) is really important. However, understanding genetic architecture more generally is also important for several reasons. Firstly, estimating the percentage of individuals who are likely to have a particular type of genetic diagnosis (including in genes we haven't yet found) can tell us where we should invest research efforts to maximise the number of diagnoses we can identify. It might also allow us to give better advice to parents about their chance of having another affected child, even if their first child doesn't get a genetic diagnosis. Secondly, it may be that not all individuals with neurodevelopmental conditions have a "genetic diagnosis", in the sense that they don't have a single large-effect variant causing their condition - it may be that their condition has more complex causes, that may be entirely or partially genetic. Understanding the causes of these individuals' conditions is still important. Research on this may ultimately help inform parents about their chance of having another affected child, about how their child's condition is likely to change as they age, and tell us about possible prevention/management strategies.

Section 2: Background to the study

6. Who conducted this study? What was their overarching goal?

We are a group of geneticists and clinician researchers working in several different research centres, primarily at the Wellcome Sanger Institute. Our goal was to better understand the genetic architecture of neurodevelopmental conditions (see questions 4 and 5). Specifically, we wanted to investigate how much of a role common variants play in different groups of people with neurodevelopmental conditions, and how they influence the chance of developing a neurodevelopmental condition.

7. What do we already know about the contribution of common variants to rare neurodevelopmental conditions?

A previous study found that common variants make up a small part of the overall chance of developing neurodevelopmental conditions (it estimated about 7%). It also showed that the same common genetic variants that affect the chance of developing a rare neurodevelopmental condition are also more common in people who have fewer years of formal education, in people with lower scores on cognitive tests, and in people with schizophrenia. However, beyond this, nothing was known about how exactly these common variants contribute to the chance of developing a neurodevelopmental condition, and whether they contribute to different extents in different groups of people with neurodevelopmental conditions.

8. What data were used in this study and how/why were they collected?

The study was conducted in over 11,500 individuals with a rare neurodevelopmental condition from two UK-based projects that focused on finding genetic diagnoses for individuals with rare disorders: the Deciphering Developmental Disorders study and the Genomics England 100,000 Genomes Project. The study also looked at over 9,100 parents of patients with neurodevelopmental conditions, and 26,800 unrelated individuals without neurodevelopmental conditions.

The Deciphering Developmental Disorders study was set up to understand the genetic causes of developmental conditions. The study brought together doctors in twenty-four regional genetics services throughout the UK and Republic of Ireland. Similarly, the 100,000 Genomes Project was an initiative to sequence the DNA of tens of thousands of people with rare diseases and cancer (together with their family members) in order to try to find the genetic causes of their conditions.

Section 3: Study design and results

9. Let’s start with the big picture - how important are common variants in neurodevelopmental conditions anyway?

This study confirmed the findings from the previous one, namely that **common genetic variation only accounts for a small proportion of the variation in chance of developing a neurodevelopmental condition**. Specifically, we estimated that this is about 10%. Despite the small overall contribution of common variants, we wanted to unpick this further and understand the nature of their contribution, and examine whether they contribute more or less in different

groups of individuals with neurodevelopmental conditions. To do this, we used polygenic scores (see question 10).

10. What are polygenic scores and how were they used in this study?

Background: Many human conditions and traits are affected by common genetic variants, including neurodevelopmental conditions, mental health conditions such as schizophrenia, and the number of years someone spends in formal education ("years of education"). Typically, individual common variants have, at most, a tiny impact on any given human trait and condition. However, the millions of common variants that are present in our DNA (sometimes called "polygenic background") can add up to have a small or moderate impact on our predisposition to a trait or condition.

We can add up the predicted impact of all common variants an individual has into something called a "polygenic score" to predict the likelihood of them having a particular condition (e.g. a neurodevelopmental condition) or trait (e.g. going to university). These polygenic scores are better predictors than any single common variant. **However, although polygenic scores are useful research tools for prediction at the population level, they are not good predictors of whether an individual person is likely to e.g. develop a neurodevelopmental condition or go to university.**

In this study, for each individual, we calculated polygenic scores for neurodevelopmental conditions based on the degree of correlation between common variants and the chance of developing these conditions. We also calculated polygenic scores for some other traits and conditions that are relevant to neurodevelopmental conditions (i.e. there is an overlap in common genetic variants that contribute to the conditions/traits). These include years of education (see question 24 for more information on why we included this) and cognitive performance (i.e. performance on cognitive tests), as well as schizophrenia. We used these polygenic scores as proxies for individuals' genetic predisposition for conditions/propensity towards traits. We compared the polygenic scores between different groups of people with and without neurodevelopmental conditions.

We emphasize that **these polygenic scores cannot be used to accurately predict any individual's propensity to a trait or condition, although they are quite useful predictors at a population level.** For example, the polygenic score for neurodevelopmental conditions only explains ~0.1% of the variation in chance of developing neurodevelopmental conditions, and the polygenic score for years of education only explains ~13% of variation in the number of years someone spends in education and ~0.5% of the variation in chance of developing neurodevelopmental conditions.

11. What did you learn about the contribution of common variants in people with neurodevelopmental conditions who have a genetic diagnosis *versus* those who don't?

We found that people who had a rare variant identified as causing their neurodevelopmental condition ('diagnosed') had, on average, a higher polygenic score for years of education and cognitive performance than those who did not ('undiagnosed'). This means that their common genetic variants predispose them to spending longer in education and scoring better on cognitive tests than individuals with a neurodevelopmental condition who don't have a genetic

diagnosis. (Having said that, many people with neurodevelopmental conditions will not attend regular schools and cognitive testing may not be appropriate.) This is probably because in diagnosed individuals, a rare genetic variant has already given them a high chance of developing a neurodevelopmental condition and therefore they are expected to require a lesser contribution from common variants. (In other words, it is in keeping with the "liability threshold model" explained below in question 23, and shown in **Supplementary Figure 19** above.)

We found that individuals with a genetic diagnosis whose parents were unaffected did not have significantly different polygenic scores from unrelated individuals without neurodevelopmental conditions ("controls") - in other words, in individuals with a very large-effect rare variant whose parents are unaffected, common variants are not contributing to their chance of developing a neurodevelopmental condition. However, those with a genetic diagnosis for whom one or both parents were affected had similar polygenic scores to affected individuals without a genetic diagnosis; thus, they have lower polygenic scores for years of education and cognitive performance than people without neurodevelopmental conditions (meaning that their common genetic variants predispose them to fewer years in education and lower performance on cognitive tests). Thus, we learnt that common genetic variants contribute to the chance of neurodevelopmental conditions in patients without a genetic diagnosis and in patients with a genetic diagnosis who have a clinically affected parent. **Supplementary Figure 20** below shows some hypothetical examples of families to illustrate these trends.

Figure S20

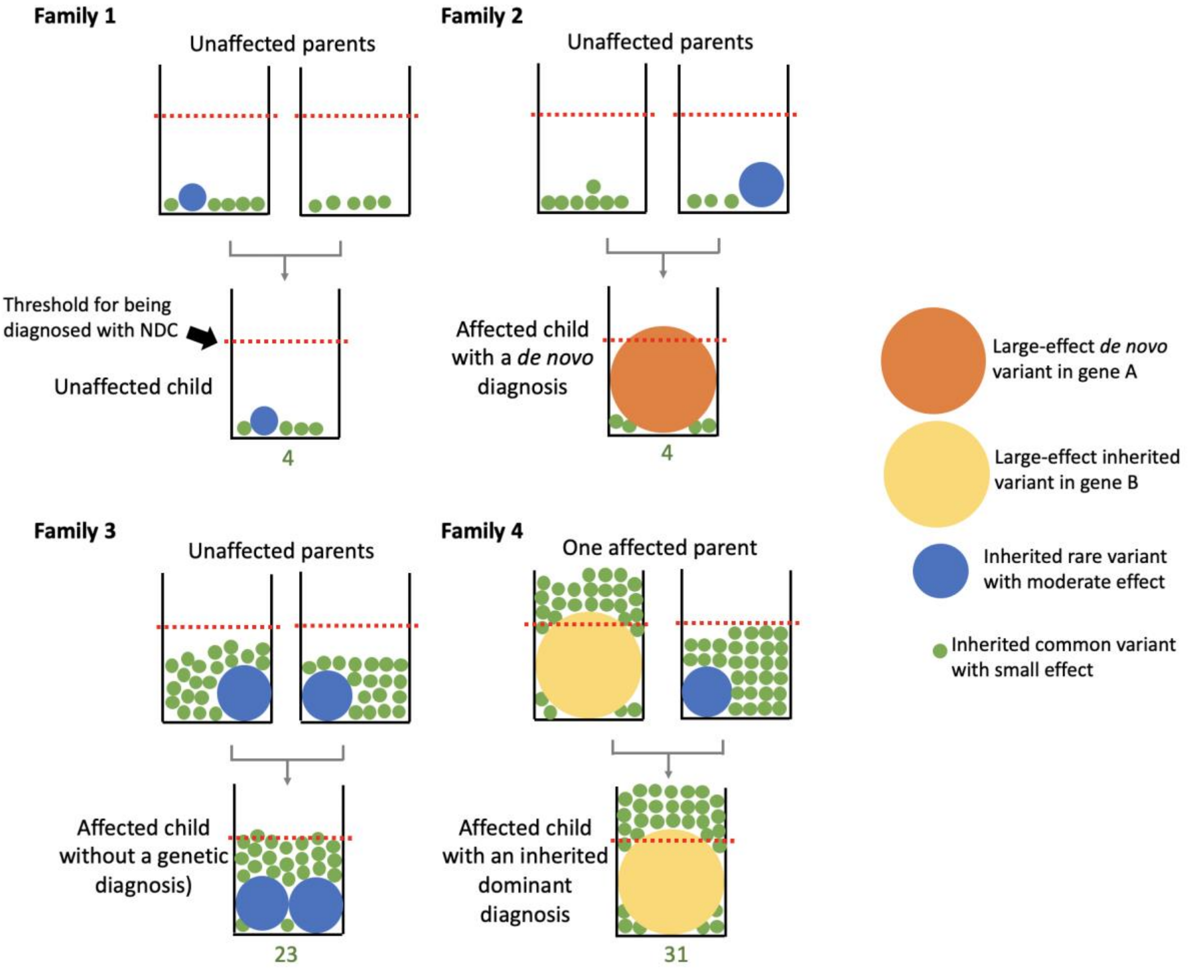

Supplementary Figure 20. Illustration of differences in polygenic risk in families with or without neurodevelopmental conditions. *In this picture, we show three hypothetical families with a child affected by a neurodevelopmental condition (families 2-4) and one hypothetical family with an unaffected child (family 1). The purpose of this figure is to exemplify the average differences in polygenic scores predisposing to neurodevelopmental conditions that we found between individuals with different characteristics (undiagnosed patients versus controls, patients diagnosed with a de novo mutation versus inherited dominant diagnosis). The number underneath each child indicates the count of green dots, which represents the degree of polygenic predisposition from common variants. In family 2, both parents are unaffected and their child has a de novo diagnosis. The polygenic score is similar to the unaffected child in family 1. In family 3, the child does not have a genetic diagnosis and has higher polygenic predisposition from common variants in addition to inherited rare variants of moderate effect. Together, these raise the child's predisposition above the threshold for developing an NDC. In family 4, one of the parents is affected as well, and the child has an inherited dominant diagnosis of a large-effect rare variant. Such children have on average the highest polygenic predisposition among all patients.*

12. What did you learn from comparing the polygenic scores of people with neurodevelopmental conditions, their unaffected parents, and controls?

Most parents of people with neurodevelopmental conditions that we studied are clinically unaffected. To our surprise, we found that, on average, unaffected parents of undiagnosed children with a neurodevelopmental condition have lower polygenic scores for years of education and cognitive performance than unrelated individuals without neurodevelopmental conditions ("controls"), similar to their affected children (i.e. their common genetic variants predispose them to spending fewer years in education and performing less well on cognitive tests). This result suggests that common genetic variants in the parents may contribute to their children's chance of developing a neurodevelopmental condition, given they have a higher predisposition than controls. However, these common variants are presumably not sufficient to cause the neurodevelopmental condition observed in their child, since the parents remain unaffected.

There could be several possible explanations for this observation. To explore one of these, we wanted to test whether the common genetic variants in the parents impacted their children's chance of developing neurodevelopmental conditions *over and above* the direct effects of the variants passed on to the children. To do this, we tested whether the parents' polygenic scores for neurodevelopmental conditions and related traits were correlated with whether or not their child had a neurodevelopmental condition, after adjusting for their child's polygenic scores. We found that for several traits related to neurodevelopmental conditions (including cognitive performance and years of education), the child's polygenic score was no longer correlated with having a neurodevelopmental condition after taking into account the parents' polygenic scores. This suggests that the common variants associated with cognitive performance and years of education *do not directly* affect a child's predisposition to neurodevelopmental conditions. However, the parents' polygenic scores were associated with whether or not their child had a neurodevelopmental condition even after taking into account the child's polygenic score. This implies that common variants that are present in the parents but not passed onto the child ("non-transmitted variants") *may* be influencing the chance of the child having a neurodevelopmental

condition - see question 13 for consideration of how this could happen. However, there is another possible explanation which we also discuss below in question 13.

For the polygenic score for neurodevelopmental conditions, we saw that there was still a significant effect of the child's polygenic score on whether or not they had a neurodevelopmental condition after adjusting for the parents' polygenic scores. This implies that the common genetic variants making up this polygenic score *do* have a direct effect on predisposition to neurodevelopmental conditions in the child.

13. Why might the non-transmitted common variants associated with cognitive performance and years of education in parents be correlated with their children's chance of having a rare neurodevelopmental condition?

This finding (described in question 12 above) could be due to two potential influences: *indirect genetic effects* and *parental similarity*.

Indirect genetic effects refer to the phenomenon in which an individual's predisposition for a trait or condition is influenced by aspects of the prenatal or familial environment that are related to their parents' or relatives' genetics. We hypothesized that one way this might happen in this context is if the common variants associated with neurodevelopmental conditions influence the mother's chance of having a premature baby. Premature birth increases the chance of the child having a neurodevelopmental condition, and we know that the mother's genetics affect her chance of having a premature birth. However, **we didn't find any evidence for indirect genetic effects acting through increased likelihood of premature birth.** In theory, other mechanisms could drive indirect genetic effects, such as deprivation, which may be correlated with the parents' polygenic scores for years of education. (People with fewer years of education tend to experience more deprivation.) However, we were unable to test this hypothesis with the data we had in this study.

Parental similarity (referred to in the paper as "parental assortment") refers to the phenomenon in which people tend to have children with partners who have similar traits to themselves. For example, people are known to choose partners who are similar to themselves in height and in level of education. If genetics contribute to the traits for which partners are similar, partners are more *genetically* similar to each other than expected by chance. This has many implications for genetic studies. One of these is that common genetic variants captured in polygenic scores for years in education are correlated with rare genetic variants that affect years in education and related traits (including the chance of neurodevelopmental conditions) (see **Supplementary Figure 21** for an explanation of how this happens). Some of these rare genetic variants may be inherited by the children. This means **we are unable to confidently determine whether the correlation between non-transmitted common variants in the parents and their child's chance of neurodevelopmental conditions is driven by indirect genetic effects or whether it is due to parental similarity. It may turn out to be a mixture of both**, and hopefully future studies will shed light on this.

Figure S21

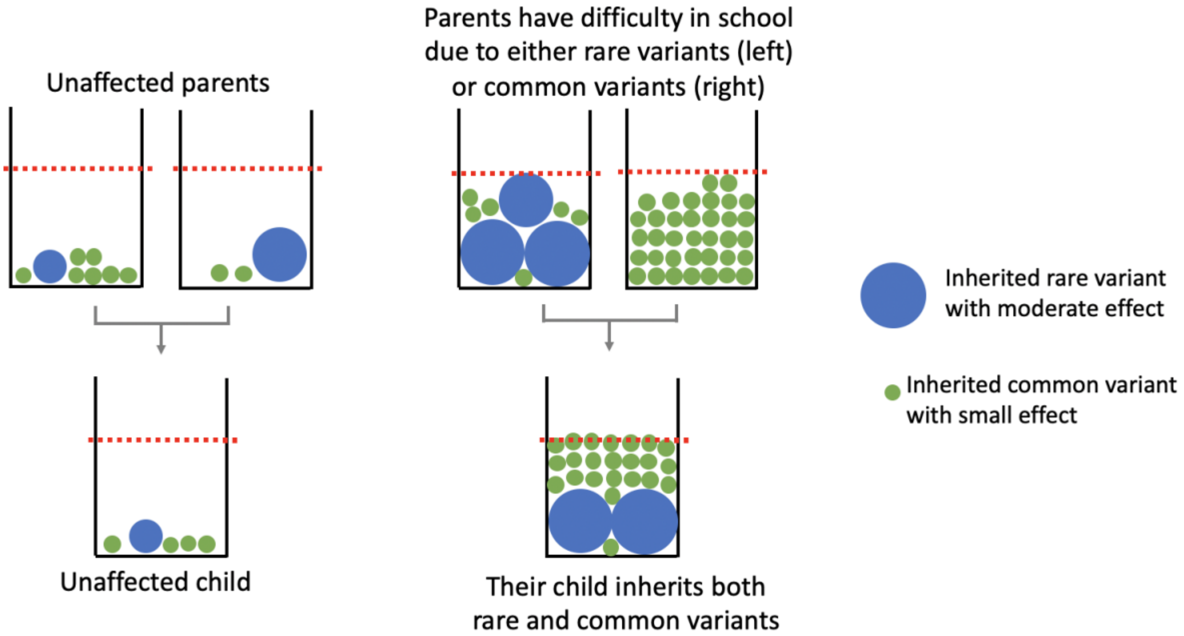

Supplementary Figure 21. Illustration of a genetic consequence of parental assortment. *Two hypothetical families in which the mother in each pair has a similar number of years in formal education to the father. Left: both parents have a low chance of developing neurodevelopmental conditions, as does their child. Right: both parents have difficulty in school due to either rare variants or common variants which contribute to their learning difficulties (reducing cognitive performance). Their child inherits some of the rare and common variants from parents. The child's risk together is then above the diagnostic threshold for a neurodevelopmental condition indicated by the red line. Imagine this with multiple couples across the population - it results in people who have more rare variants that contribute to learning difficulties also having more common variants contributing to learning difficulties.*

14. What are the limitations of your study?

Like all scientific studies, there are several limitations to this work:

- As noted above, the polygenic scores we used are only very weak predictors of neurodevelopmental conditions - each of them explains <1% of the variation in chance of developing a neurodevelopmental condition. This limits our ability to detect differences in the polygenic scores between different groups. Furthermore, the predictive power of polygenic scores depends partly on the number of samples in the original genetic study from which they were derived (i.e. genome-wide association studies, described below). The sizes of the underlying genetic studies differ between traits, ranging from several hundred thousand to over one million people. This makes comparisons between the relative effects of different polygenic scores difficult.
- We have brought together a range of rare neurodevelopmental conditions in this analysis (for example, individuals with varying levels of intellectual disability, individuals with seizures versus without seizures), which may have limited our ability to detect effects. It may be that the contribution of common genetic variants differs between different neurodevelopmental conditions.

- Parental similarity for years in education and/or cognitive ability could be influencing our results. As described in question 13, parental similarity could cause the correlation between non-transmitted common variants in the parents and whether or not their children have a neurodevelopmental condition. More generally, the correlation we observed between common and rare variants predisposing to neurodevelopmental conditions means that the findings in the paper about common genetic variants may be partly driven by the rare genetic variants which are correlated with them, which are the ones actually predisposing to neurodevelopmental conditions.

Section 4: Implications of the study

15. My child has a neurodevelopmental condition. What do the findings of this study mean for my child and our family?

The findings from this study are not informative about any individual family or person with a neurodevelopmental condition. This study describes a series of statistical trends relating common genetic variation to neurodevelopmental conditions at the population level. However, although statistically significant, the relationships we found between common genetic variants and neurodevelopmental conditions are weak, meaning we cannot make accurate statements about any particular individual and their chance of a neurodevelopmental condition based on their common variants, nor can we decide on treatment or clinical management based on their common variants. It is possible that in the future, after more research is done, doctors might start to use common variants to help uncover the cause/s of an individual's neurodevelopmental condition, to inform parents about how their child's condition is likely to change as they age, to tell us about possible prevention/management strategies, or to give parents advice about their chance of having another affected child. However, currently, this is not possible.

16. Can you now predict who will have a neurodevelopmental condition based on their polygenic scores?

No, we cannot predict who will have a neurodevelopmental condition based on their polygenic scores. First, as explained in question 1, many neurodevelopmental conditions are caused by a single genetic variant that is not commonly found in the population, and indeed may only be seen in a single individual. Additionally, as explained in question 2, some neurodevelopmental conditions are caused by environmental factors (e.g. fetal alcohol syndrome, or Zika virus). Polygenic scores can only explain a trait to the extent that the trait is influenced by common genetic variants. Our estimates suggest that about 10% of the predisposition to neurodevelopmental conditions is due to common genetic variants, and polygenic scores currently only capture a small part of this (see questions 9 and 10 above). Thus, we will never be able to predict neurodevelopmental conditions very well using common genetic variants alone. **It is unlikely that common genetic variants alone are the sole cause of the condition for any of the individuals included in this study.**

17. If the association between polygenic scores and predisposition for neurodevelopmental conditions is so small at the population level, why is this interesting?

There are many reasons why this is still interesting despite the small associations between polygenic scores and neurodevelopmental conditions:

i) While the contributions of common genetic variants may be small, it does not mean they are unimportant. All predisposing factors must be considered if we are to fully understand the causes of neurodevelopmental conditions. Despite huge advances in finding genetic causes for rare conditions, the majority of individuals with neurodevelopmental conditions remain undiagnosed. There is still a lot we do not know about potential causes of these undiagnosed conditions, and contributions from polygenic scores composed of common genetic variants may be a factor.

ii) These associations are small in part because we do not have enough data to accurately estimate the effects of common genetic variants on neurodevelopmental conditions and related traits. As the number of individuals included in genome-wide association studies increases, we will be able to produce more predictive polygenic scores. However, as noted in question 16, since common variants in total explain a small proportion (currently estimated about 10%) of the variation in chance of developing a neurodevelopmental condition, polygenic scores will never be very accurate predictors of who will develop a neurodevelopmental condition.

18. How could the results of this study be used to help patients with rare neurodevelopmental conditions and their families?

Clinicians and families often find that understanding the reasons an individual has a neurodevelopmental condition is beneficial, both psychologically and medically. These results bring us closer to understanding all the factors that contribute to neurodevelopmental conditions, particularly in those who are currently undiagnosed by modern genetic techniques. However, the findings from this study are unlikely to be incorporated in clinical settings in the near future. In the longer term, as our understanding of the role of polygenic score in neurodevelopmental conditions improves, these findings might help provide more families with an explanation for their child's neurodevelopmental condition and become part of precision testing strategies which look to incorporate the full picture of a person's common and rare genetic variants and environmental influences on their neurodevelopmental condition.

19. How might the results of this study or the information in this document be misinterpreted and why are these interpretations incorrect?

Misinterpretation 1: People develop neurodevelopmental conditions because their parents spent less time in education and don't provide a good enough environment for them.

This is incorrect. Neurodevelopmental conditions are most often caused by a rare genetic variant, and such variants are passed on randomly from parents to children, or arise for the first time in the child (*de novo*). Occasionally neurodevelopmental conditions are caused by viruses, exposure to drugs in pregnancy or by premature birth. When identifying the cause of an

individual child's neurodevelopmental condition, it is important to have a full medical assessment before any conclusions on the cause of that child's condition are made.

In this paper, we found evidence that common genetic variants that predispose to fewer years of education may also affect the chance of an individual having a neurodevelopmental condition. However, two points are important to bear in mind. Firstly, this only accounts for a very small proportion of the overall predisposition for these conditions. Secondly, parents have no control of which common genetic variants they carry or pass on to their children. Although we found that children's chances of having neurodevelopmental conditions is correlated with common genetic variants in their parents that are correlated with fewer years of education and that are not transmitted to the children, the explanation for this is unclear, as described in question 13.

Misinterpretation 2: No patient with a neurodevelopmental condition has a single cause for their condition i.e. a mutation in a single gene is insufficient to cause the condition - it will only do so in the context of a particular genetic background.

This is incorrect. As noted in question 1, many people with neurodevelopmental conditions have a single genetic cause for their condition, regardless of their genetic background. In this study, we mainly investigate neurodevelopmental conditions that cannot be explained by a single genetic cause.

Misinterpretation 3: Because my child has a neurodevelopmental condition, they or my other children are likely to develop ADHD or schizophrenia.

In this study, we found that common variants that are correlated with increased chance of neurodevelopmental conditions are also correlated with increased chance of ADHD and schizophrenia. **However, this is a result at the population level, and it does not have any meaningful implications for individuals about their or their families' chance of developing these conditions.** This is because common variants that are associated with ADHD and schizophrenia explain less than one quarter of an individual's chance of these conditions. Any increased chance from more common variants predisposing to ADHD or schizophrenia are unlikely to be meaningful for an individual as there are many other factors that make up a larger proportion of chance of these conditions.

Misinterpretation 4: My child developed a condition because they were born prematurely.

Premature birth, particularly before the age of 32 weeks, is known to be associated with increased chance of neurodevelopmental conditions. It may be in these cases that the complications associated with the premature birth themselves cause the condition. However, not all children born prematurely develop neurodevelopmental conditions and other factors may play a role.

Usually very premature birth is a random event that cannot be prevented and most often the cause of it is not identified. There is some evidence that certain genetic conditions may cause babies to be born prematurely, so in some children (we don't know what percentage), it may not

be the premature birth itself causing the condition, but rather a genetic difference may have caused both the premature birth and the condition.

The chance of premature birth causing neurodevelopmental conditions reduces with each additional week of pregnancy. Therefore, it is more likely that children born moderately premature (e.g. after 32 weeks) may have other environmental factors and/or genetic factors that interact to cause the condition.

Misinterpretation 5: My child developed a condition because of medications I took in pregnancy.

This is unlikely to be the case. Most medications are not known to cause neurodevelopmental conditions. A small number of medications, such as anti-epileptic drugs, are known to increase the chance of certain conditions in children, but as a consequence, doctors avoid prescribing them to pregnant women unless they are essential to the mother's health.

Even in cases where a child is exposed to a medication known to cause a neurodevelopmental condition, not all such children will develop a neurodevelopmental condition. It is important to have a thorough medical history, examination and assessment with a trained specialist before any link between an individual neurodevelopmental condition and a medication is made.

Misinterpretation 6: My child developed a condition because I smoked very occasionally in pregnancy/had a few glasses of wine.

This is unlikely to be the case. Heavy drinking in pregnancy can cause a condition called 'fetal alcohol syndrome' which involves neurodevelopmental problems. However, even if a mother does drink heavily while pregnant, her child will not always be born with this condition. It's unlikely that a small amount of alcohol or smoking would be a major factor contributing to your child's condition.

It is important to have a thorough medical history, examination and assessment with a trained specialist before any link between an individual neurodevelopmental condition and use of alcohol/tobacco during pregnancy is made.

The Royal College of Obstetricians and Gynaecologists has further information on alcohol and smoking for those who are currently pregnant .

Core concepts and terminology used above

20. What are rare neurodevelopmental conditions?

Rare neurodevelopmental conditions are a group of conditions that are first noticed during childhood and affect the growth and development of the brain. They can cause children to have intellectual disability, seizures and a small head size, and delays in achieving developmental milestones, such as walking and talking. Children with these conditions are likely to need educational, health and social support throughout their lives. The individual conditions that make

up this group are often very rare and may only affect a handful of individuals in the UK or even the world but cumulatively affect ~1% of newborns.

The patient organisation UNIQUE exists to provide support and information to those with rare chromosomal and genetic conditions that cause developmental delay and/or an intellectual disability. More information on many individual rare neurodevelopmental conditions can be found on their website: https://rarechromo.org/ or by emailing help@rarechromo.org.

21. What is a genetic "variant"?

Our DNA is essentially a long sequence of letters, and genetic variants are differences in those letters. All of us have millions of genetic variants in our DNA, and these are partly what makes us different from one another. These differences can be as small as one letter being different or missing, or can be larger differences or rearrangements of stretches of DNA, a little like a paragraph being removed, repeated or rearranged. Some genetic variants are commonly seen in the general population - "common variants" - while some are rarely seen in the general population - "rare variants". A variant that is seen in more than 1% of people is usually considered to be common.

22. What are Genome Wide Association Studies?

In a genome-wide association study, researchers look at whether people with a particular trait or condition tend to have certain common genetic differences. These studies focus on single-letter differences in the DNA, known as single-nucleotide polymorphisms, which are commonly seen in the population. Genome-wide association studies have been successful in identifying common genetic differences associated with a range of different conditions and traits, and they are very commonly used in genetic research.

23. What is the liability threshold model?

The liability threshold model is a theory to help understand a person's chance of developing a condition, such as a neurodevelopmental condition. In this model, an individual's genetics (both rare and common) and environmental experiences influence their brain development and add up to give their overall chance of developing a neurodevelopmental condition. If there are enough factors that affect the development of their brain, they will develop a condition. The theory has been explored in a range of different medical conditions, including schizophrenia and rare heart disease.

24. Why did you look at years of education as a trait?

Individuals who have neurodevelopmental conditions often have intellectual disability. Thus, it seemed plausible that some of the common variants that affect the chance of neurodevelopmental conditions may overlap with those that affect cognitive ability in the general population. Indeed, a previous study found that common variants that affect scores on cognitive tests in the general population also affect the chance of an individual developing a rare neurodevelopmental condition, as do the common variants that affect the number of years someone spends in education. In genetic and social science research, the number of years someone spends in education is often used as an imperfect proxy for cognitive ability, because it is correlated with it and it is much easier to collect data on years of education (by simply asking

individuals how many years they spent in education) than to administer time-consuming cognitive tests. This means that we have much larger sample sizes available for genome-wide association studies of years of education (currently around 3 million people) than cognitive ability (around 300, 000 people), which better enables us (gives us much more statistical power) to estimate the effect of genetic variants on this trait. We made use of these genetic studies to allow us to calculate polygenic scores for years of education, which we used in our study as predictors (albeit not very good ones) of people's genetic propensity towards education (as a proxy for their genetic propensity for cognitive ability).

25. What do you mean by "ancestry" in the paper, why is it important in genetic studies, and why did you focus on people with British ancestry?

"Ancestry" is a construct based on shared DNA inherited from one's ancestors. It is not the same as race or ethnicity, which are social constructs used to categorize people based on a shared history, appearance, geography, culture, language or other factors. Ancestry is not a clear-cut category. A group of individuals can be divided into numerous "ancestry groups" based on genetic similarities. In this paper, we refer to "genetic ancestry" based on genetic similarity between the individuals under study and a set of reference individuals who are known to originate from particular geographic regions.

Often, to understand a trait or condition, we look for how common particular genetic variants are between two groups, for example, between patients with neurodevelopmental conditions and individuals without neurodevelopmental conditions from the general population ("controls"). The goal is to find variants that are more commonly seen among people with the trait/condition and that are likely part of the reason that person has the trait/condition. If the two groups we are comparing have genetic differences *by chance* due to having different genetic ancestries, this will give us misleading results. Specifically, if the trait/condition is influenced by environmental factors that are correlated with ancestry, we will see many genetic differences that are also correlated with the trait/condition, but they do not causally affect it.

To prevent our results being confused in this way by genetic ancestries, standard practice in genetic studies (including genome-wide association studies, results from which are used to construct polygenic scores) has been to only analyze the largest genetically homogenous subset of the data, which in practice means only including individuals from the ancestry group most heavily represented in the sample. Most individuals in the Deciphering Developmental Disorders study and the 100,000 Genomes Project have genetic ancestries similar to those whose grandparents originate from the UK. Thus, we restricted our analyses to this subset of individuals, whom we identified based on their genetic similarity to people from an external reference dataset who were known to have British ancestries.

It is well recognised in genetic research that there is an underrepresentation of people from Non-European ancestries. There are a number of large scale efforts to try to correct this. Moreover, many efforts are currently underway to develop statistical methods to analyze multi-ancestry cohorts and individuals whose genetic ancestry is a mix of different populations. However, at present, for this study, we unfortunately do not have enough samples to perform a meaningful statistical analysis of the role of common variation in neurodevelopmental conditions for individuals other than those with British ancestries. We hope this will be possible in the future and highlight it as future work.

Supplementary Note 2: Phenotypic comparisons of the cohorts

We compared the sex, age and Human Phenotype Ontology (HPO) terms between DDD and GEL patients with neurodevelopmental conditions included in the study. There was no significant difference in the sex of probands in DDD compared to GEL (DDD 41.0% female versus GEL 39.3% female, Fisher's exact test p-value = 0.08). DDD probands, however, were significantly younger at assessment than probands in GEL (DDD mean 7.74, standard deviation 6.29; GEL mean 10.78, standard deviation 9.10; Welch's *t*-test two-sided p-value = 9.51×10^{-71}) (**Extended Data Figure 3A**).

DDD probands had fewer HPO terms on average than GEL probands (DDD mean 7.28, standard deviation 4.04; GEL mean 9.34, standard deviation 5.34) (**Extended Data Figure 3B**). This difference was significant after controlling for age at assessment and sex in a multiple Poisson regression (p-value = 6.31×10^{-251}). We then compared the prevalence of HPO terms in overarching HPO chapters (downloaded from https://hpo.jax.org/data/ontology in September 2023) and some selected phenotypes in DDD and GEL, controlling for age and sex. There were significant differences between the two cohorts for numerous HPO chapters and chosen terms (**Extended Data Figure 3C**). Three notable differences between the cohorts were the greater proportion of GEL probands recorded as having autistic behaviours (31% of GEL vs 16% DDD; p-value = 5.4×10^{-59}), speech and language impairment (66% GEL vs 25% DDD; p-value < 2.2×10^{-308}), and ID/DD cases of unspecified/unknown severity (80% GEL vs 33% DDD; p-value < 2.2×10^{-308}).

Differences in HPO term prevalence may reflect differing practices in how terms were recorded. The DDD recruitment form asked the recruiting clinicians (who were all clinical geneticists) to record HPO terms they thought were relevant to the child's condition. In contrast, patients were recruited into GEL by a range of clinical practitioners (clinical geneticists, other speciality doctors, clinical/research nurses, genetic counsellors), who were asked to select 'yes/no/unsure' against a set of HPO terms considered to be common for patients in the phenotypic category into which the patient was recruited (e.g. 'intellectual disability'/'epilepsy plus other features'/'malformations of cortical development'). Clinical geneticists may have been more likely to select terms focused holistically on signs and symptoms of the monogenic presentation rather than non-specific HPO terms or terms relating to only one organ system (as a single organ/system specialist doctor may select). Additionally, the clinical geneticists recruiting to DDD were likely more focused on the degree of severity of ID/DD than the healthcare professionals recruiting to GEL, since they were asked a specific question about it as it was relevant to the inclusion criteria for DDD. (Patients could be included if they had moderate/severe ID/DD or if they had mild ID/DD plus other abnormalities.) These differing coding practices are likely to have artificially created HPO term differences between the cohorts rather than reflecting true clinical differences. To test this, we compared the prevalence of HPO terms in GEL versus DDD for neurodevelopmental probands who were recruited to both cohorts (N=789), controlling for age at recruitment to that study and sex. We saw similar significant differences in the HPO terms recorded for this identical set of probands between the two programs, re-enforcing these differences were likely created by differences in recording practises rather than actual differences between the GEL and DDD cohorts (**Extended Data Figure 3D**). However, it is possible that part of the reason for the increased prevalence of autistic behaviour in GEL is that the GEL recruitment of probands began a few years later than

DDD (recruitment 2015-18 versus 2011-15), and the rates of diagnosed autism have been increasing over time[27].

Supplementary Note 3: Genome-wide significant hits from the GWAS meta-analysis of neurodevelopmental conditions

We found two genome-wide significant ($p<5x10^{-8}$) loci in the GWAS meta-analysis of neurodevelopmental conditions (**Extended Data Figure 4**), although no single nucleotide polymorphism (SNP) passed genome-wide significance in either DDD or GEL alone. The locus on chromosome 22 is in an intron of *SREBF2*. Variants in this gene have been reported to be significantly associated with brain-related traits including intelligence[28–30], mathematical ability[18], and schizophrenia[19] (GWAS Catalog https://www.ebi.ac.uk/gwas/, queried on 24 Oct 2023). The lead SNP associated with neurodevelopmental conditions (rs2284084, odds ratio of risk allele T = 1.16, $p = 1.71x10^{-8}$) is in weak LD with rs2267442 ($r^2 = 0.22$ estimated in 1000 Genomes GBR-ancestry subpopulation), which is associated with decreased intelligence[28,29].

The other genome-wide significant locus is located upstream of gene *PWRN4*. Lead SNP rs113446150 (odds ratio of risk allele A = 1.12, $p = 4.04x10^{-8}$) is in high LD with a SNP associated with height (rs4396492; $r^2 = 0.89$)[31] reported in the GWAS Catalog. The lead SNP is a splice quantitative trait locus (sQTL) for *PWRN4* specific to the pituitary gland in GTEx. Other variants in *PWRN4* are reported to be associated with age at menarche[32].

Formal colocalization would be required to determine whether the GWAS hits from our GWAS of neurodevelopmental conditions are the same as those reported for these other traits, but the power of these is likely to be limited by the small size of our GWAS. None of the other variants within 500 kb of and in moderate or high LD with our lead SNPs (LD $r^2 > 0.5$ in 1000 Genomes GBR-ancestry subpopulation) were reported to have a significant association in the GWAS Catalog.

Supplementary Note 4: Potential ascertainment biases in control cohorts and their effects

In this section, we first describe how the various control cohorts were recruited and discuss whether they are likely to be biased according to educational attainment. We then consider the extent to which this affects our comparisons with these groups, and conduct a sensitivity analysis using MCS.

Likely ascertainment biases in control cohorts

The four control cohorts used in this study were as follows:

- The UK Household Longitudinal Study (UKHLS), which was a continuation of the British Household Panel Survey[33]. The study aimed to capture a representative sample of people living in the UK and to collect longitudinal socioeconomic and other data on them. Individuals were selected to include in the study based on their postcodes, and incentivised with monetary reward. In waves two and three of the study (2010-2012),

participants aged 16 and over were invited to take part in a nurse visit, at which blood samples were taken if participants consented[34], and used to extract DNA. Those with genotype data were slightly enriched for having a university degree compared to the 2011 UK census (33.8% versus 27.2%).

- Adult cancer patients and relatives of other rare disease patients not affected by neurodevelopmental conditions or DDD-like developmental disorders from the Genomics England (GEL) 100,000 Genomes Project. GEL participants were recruited from the National Health Service, which is free at the point of care to all residents of the UK. Thus, in theory, we would not expect the control samples chosen from this cohort to be biased according to educational attainment, unless there are education/cognition-related risk factors for cancer or for rare conditions other than neurodevelopmental conditions, or unless patients who agreed to participate tended to come from a particular socioeconomic background.
- The Avon Longitudinal Study of Parents and Children (ALSPAC), a birth cohort which recruited 14,775 babies born in the Avon region of southwest England in 1991-1992. About three-quarters of those eligible agreed to participate[10]. ALSPAC mothers are known to have slightly higher average socioeconomic status (SES) than mothers in the whole of Avon and of Britain[35]. Most of the mothers' and children's DNA samples were obtained from blood samples taken at birth. Most of the fathers' DNA samples were obtained at clinics when their children were teenagers or in their early 20s.
- The Millennium Cohort Study (MCS), a birth cohort which recruited 18,827 children born 'between 1 September 2000 and 31 August 2001 (for England and Wales), and between 24 November 2000 and 11 January 2002 (for Scotland and Northern Ireland), alive and living in the UK at age 9 months, and eligible to receive child benefit at that age' [22]. Certain subgroups were intentionally over-sampled, namely children living in disadvantaged areas, children of ethnic minority backgrounds, and children growing up in the smaller nations of the UK[22]. DNA samples were taken when the probands were aged 14, at sweep 6. Only 11,872 (63%) of the original sample participated in this sweep, and they were biased towards families with higher SES compared to eligible families who did not participate[36]. Thus, this biased attrition might be expected to reduce the bias introduced by the initial over-sampling of low-SES families, but it is unclear to what extent.

Comparing average PGS_{EA} between these different control cohorts can give us a sense of the relative degrees of education-related ascertainment bias (**Extended Data Figure 6A**; **Supplementary Table 6**). On average, we see that differences in PGSs between the GEL controls and UKHLS controls are small, although significant (difference in mean PGS = -0.042 SD, two-sided *t*-test p = 0.002). Within the GEL controls, we found that the cancer patients had a slightly higher average PGS_{EA} than the relatives of other rare disease probands without neurodevelopmental conditions (difference in mean PGS = 0.05 SD, two-sided *t*-test p=0.013). ALSPAC children were very similar to UKHLS and GEL controls in their average PGS_{EA} (on average 0.027 SD higher than GEL and 0.015 SD lower than UKHLS; two-sided *t*-test p=0.06 and 0.34), but MCS children had lower average PGS_{EA} than all of the control groups (difference ranging from 0.090 to 0.132 SD, two-sided *t*-test $p < 7x10^{-9}$), likely reflecting the cohort's deliberate over-sampling of low-SES households.

In ALSPAC and MCS, as well as amongst the cases with neurodevelopmental conditions, trio probands had significantly higher PGS_{EA} than probands who did not have genetic data on both

parents (**Extended Data Figure 6B; Supplementary Table 6**) (average difference in PGS_{EA} between trio and non-trio probands > 0.20 SD, two-sided *t*-test $p<3x10^{-14}$). This likely reflects the fact that families with low SES backgrounds are more likely to be single-parent households[37]. Additionally, in ALSPAC, it may reflect the fact that most of the paternal DNA samples were taken when the children were teenagers or older, so the fathers who were still engaged in the study at this point might have been biased towards higher educational attainment.

Sensitivity analyses to assess the effect of ascertainment bias in controls

Unsurprisingly given the results above, the estimates of differences between probands with neurodevelopmental conditions and controls are sensitive to the selection of control samples. For example, the set of all probands with neurodevelopmental conditions had significantly lower PGS_{EA} than all control groups considered (two-sided *t*-test p-value < $2x10^{-7}$), but the difference in mean ranged from -0.09 SD with the MCS children (two-sided *t*-test $p=5.2x10^{-9}$) to -0.5 SD with the ALSPAC trio children ($p=6.3x10^{-60}$; **Extended Data Figure 6A**, **Supplementary Table 6**). Since it is impossible to know which (if any) of these control cohorts are really unbiased samples of the general population, we turned to a different approach using MCS.

MCS has calculated weights based on various sociodemographic variables (e.g. SES) which can be used to reweight the individuals in the study to make them representative of the general population, in order to calculate adjusted prevalences/mean estimates and robust standard errors[22]. These include the initial sampling weights, which are intended to reweight the initial sample gathered in the first sweep, and non-response weights for each sweep. We constructed new weights for the set of MCS children who had genetic data (or specifically, the unrelated GBR-ancestry sample shown in **Extended Data Figure 6A**) and for the set who had genetic data on themselves and both parents (i.e. trio children used in **Figure 4** and **Extended Data Figure 6A**) (see **Supplementary Methods**). Applying these to recalculate the PGS_{EA} for all MCS children adjusting for sampling and non-response bias, the mean PGS_{EA} did not significantly change, with a mean PGS_{EA} of -0.1069 (standard error, se=0.0129) prior to weighting versus -0.073 (0.0132) after (p=0.065, Wald test). When conducting the same analysis for MCS trio children, the mean PGS_{EA} significantly decreased, with a mean of 0.012 (0.020) prior to weighting versus -0.055 (0.020) after (p=0.0178; **Extended Data Figure 6C**). Prior to adding weights, the difference in mean PGS_{EA} between all children versus only those in trios was highly significant ($p<10^{-5}$), but was fully attenuated after weighting (p=0.45). (**Extended Data Figure 6C**). Many of the subsets of probands with neurodevelopmental conditions considered showed significantly lower PGS_{EA} than the weighted MCS mean, including the undiagnosed probands (Δ = -0.17, $p=3.3x10^{-21}$) and diagnosed probands with affected parents (Δ = -0.258, p=0.0022); however, the diagnosed probands with unaffected parents or with *de novo* diagnoses did not significantly differ from the weighted MCS sample (**Extended Data Figure 5A**; **Supplementary Table 7**).

Supplementary Note 5: Examining sex differences in polygenic risk

There are 1.5-times more male (N=6,879) than female (N=4,694) probands with neurodevelopmental conditions in DDD and GEL combined, which is consistent with the "female

protective effect" whereby females either have lower mean liability for neurodevelopmental conditions, or require higher liability than males to get a diagnosis[38–47]. Indeed, female patients in DDD are more likely to get a monogenic diagnosis[21] and have a higher burden of damaging *de novo* mutations than males[48]. We did not detect any significant differences in PGS between male and female undiagnosed probands with neurodevelopmental conditions for any PGS (**Extended Data Figure 8A**). This is not inconsistent with recent work in autism: Wigdor *et al*. found that autistic females have higher autism PGS than males after accounting for co-occurring ID[44], but saw no significant difference otherwise. Similarly, Warrier *et al*. showed that amongst autistic individuals without ID, females over-inherited more polygenic risk for autism than males[49]., but did not detect a difference when including individuals with ID. In our data, although $PGS_{NDC,DDD}$ was significantly over-transmitted in females (pTDT deviation = 0.10, p-value = 0.0078, N=589 trios in DDD and GEL combined) but not males (pTDT deviation = 0.036, p-value = 0.27, N=978 trios), there was no significant difference in the pTDT deviation between them (two-sided z-test p=0.19) (**Extended Data Figure 8C**). Notably, in contrast to our findings, Antaki et al. showed that autistic females have higher values for a combined PGS for autism + educational attainment + schizophrenia than autistic males[50]. Our findings from these sex comparisons emphasise that ID, which is present in the majority of DDD and GEL NDC probands, has a distinct genetic architecture from autism more broadly.

In families of autistic children in whom neither parent has a known autism diagnosis, mothers were found to have a higher PGS for autism than fathers, possibly reflecting the fact that women can 'tolerate' a higher burden of risk alleles before manifesting the phenotype due to the so-called "female protective effect"[44]. Thus, we also compared the five NDC-related PGSs between mothers and fathers, focusing on unaffected parents of undiagnosed probands (**Extended Data Figure 8B**). We found no significant differences.

Supplementary Note 6: Results for polygenic scores based on the within-family GWAS of educational attainment

Effects of genetic variants estimated using a GWAS in a large sample of unrelated individuals capture direct effects of inherited variants, as well as effects of population stratification[51], parental assortment (or assortative mating)[52], and indirect genetic effects from family members, especially from parents[53]. Polygenic scores derived from population-based GWASs thus show inflated associations with the phenotype[54]. Within-family GWASs should capture only the direct genetic effects. However, the sample sizes of within-family GWASs are usually smaller than population-based GWASs, so they produce less well-powered polygenic scores. We repeated some of the analyses using summary statistics from a within-sibship GWAS of educational attainment[20]. This GWAS should be immune to confounders other than indirect effects from siblings, which are estimated to be much smaller compared to indirect genetic effects from parents[55].

The GWAS summary statistics for educational attainment from within-sibship study showed a significant genetic correlation with neurodevelopmental conditions (r_g = -0.702, p = 0.0056), which was similar to that using population-based GWAS (r_g = -0.654, p=4.9x10^{-12}). A polygenic score derived from the within-sibship GWAS (PGS_{EAsib}) was significantly associated with NDC case-control status within GEL samples (p = 0.0015, variance explained R^2 = 0.05%), although

it was not significant when comparing DDD patients with UKHLS control individuals (p=0.2). This reflects the decreased power of this within-sibship study which was conducted in ~55,000 individuals compared to the population-based GWAS for educational attainment conducted in ~766,000 individuals[56] that we used in the main Results. The less predictive PGS is consistent with the attenuated estimate of SNP heritability reported in the within-sibship GWAS compared to the population-based GWAS (3.6%, 95% CI: 2.0%–5.2% versus 11.2%, 95% CI: 10.6%–11.8%). PGS_{EAsib} did not show a significant difference (p=0.16) between NDC patients with versus without a monogenic diagnosis, in contrast to PGS_{EA} derived from the population-based GWAS (**Figure 2A**). Similar to PGS_{EA} (**Figure 3A**), probands' PGS_{EAsib} did not show a significant deviation (p=0.75) from parental average PGS_{EAsib}.

Next, we reran the "trio model" using PGS_{EAsib} to assess the effects of probands' PGS while controlling for parents' PGS, and to examine the association between parental non-transmitted alleles and NDC risk in probands (**Figure 4**). Similar to PGS_{EA}, we observed a significant non-transmitted coefficient in mothers using PGS_{EAsib} (p=3.8x10^{-5}, beta=-0.122) (although not in fathers (p=0.14, beta=-0.043)), and we still observed no significant direct genetic effect (p=0.77, beta= 0.0099).

In analyses to assess the consequences of parental assortment, PGS_{EA} showed significant correlations with rare variant burden in constrained genes within/between different sets of individuals (**Figure 5**). Using PGS_{EAsib}, we observed a nominally significant correlation (p=0.03, r = -0.024) with rare variant burden within unaffected parents in the expected direction, but did not observe this within NDC probands or between parents. As expected, we did not observe significant correlations using rare synonymous variants as the negative control.

In summary, the power of the within-sibship GWAS of EA is limited and PGS_{EAsib} derived from this GWAS is less predictive of NDC risk than PGS_{EA} derived from the population-based GWAS. Nonetheless, analyses with summary statistics from the within-sibship GWAS still recapitulate some of the same conclusions reached using the population-based GWAS. For example, importantly, the genetic correlation with NDCs was not attenuated. The fact that we were able to recapitulate the significant effect of non-transmitted alleles at least in the mother indicates that our original result with PGS_{EA} in the trio model was not due, for example, to uncontrolled stratification in the original population-based EA GWAS which happens to be correlated with stratification within our own samples.

Supplementary Note 7: Exploring the role of prenatal risk factors in mediating common variant risk

Genetic correlations

We hypothesized that the correlation between non-transmitted parental alleles and risk of neurodevelopmental conditions (**Figure 4**) may be partly mediated by the prenatal environment, aspects of which are associated with risk of these conditions. For example, being born prematurely is a risk factor for neurodevelopmental conditions[57–59], and we see a negative genetic correlation between preterm delivery[60] and educational attainment (r_g=-0.30; p=2x10^{-10}). This correlation may be partly explained by the fact that women with lower educational levels

are more likely to have risk factors for premature birth, such as exposure to tobacco smoke during pregnancy[61,62]. Within DDD, lower PGS_{EA} and $PGS_{NonCogEA}$ in mothers was significantly associated with the proband having been born prematurely (**Extended Data Figure 9D**).

To explore the potential contribution of prenatal risk factors to polygenic risk for neurodevelopmental conditions, we calculated genetic correlations between our GWAS meta-analysis and risk factors for which GWASs were available: preterm delivery[57–60], smoking[59,63], alcohol use[59,63], gestational hypertension[59,64] and sleep apnoea[65–67] (**Extended Data Figure 9A**). We observed significant genetic correlations between neurodevelopmental conditions and preterm delivery (r_g=0.58; p=0.004) and smoking initiation (r_g=0.27; p=2x10^{-5}). After conditioning on the educational attainment GWAS with GenomicSEM, the genetic correlation with smoking initiation was no longer significant (r_g=0.04; p=0.62), while that with preterm delivery was still nominally significant (r_g=0.48; p=0.04) (**Supplementary Table 11**). The latent EA factor explained the majority (90%) of the original genetic correlation with smoking initiation but only 35% of the genetic correlation with premature delivery (**Extended Data Figure 9B**, **Supplementary Methods**). Conversely, after conditioning on the preterm delivery GWAS, the genetic correlation between educational attainment and neurodevelopmental conditions was still highly significant (r_g=-0.60, p=8x10^{-7}).

Testing whether prematurity mediates the effect of non-transmitted common alleles

Based on these results, we hypothesized that prematurity may partly mediate the correlation between non-transmitted common variants associated with educational attainment and risk of neurodevelopmental conditions. To test this, we reran the "trio model" from **Figure 4** using PGS_{EA} in several different ways, restricting to undiagnosed DDD cases and MCS controls for which information on gestational age at birth was available (**Supplementary Figure 5**). After excluding probands born prematurely from both cases (16%) and controls (6%), the effect of non-transmitted parental alleles was slightly attenuated compared to that seen when using all trio probands, particularly in mothers (β_{mother} = -0.14 [-0.23 – -0.06] and p=6x10^{-4} using all samples, versus β_{mother} = -0.11 [-0.20 – -0.02] and p=0.02 after excluding probands born prematurely). A similar result was obtained when using all probands but controlling for whether they were born prematurely. We would expect such attenuation if there were an indirect genetic effect of PGS_{EA} mediated partly via premature delivery. However, the effect sizes were not significantly altered for any PGS in either analysis compared to the original analysis of all trio probands (z-test p>0.05). Thus, there is no significant evidence from these data that indirect genetic effects mediated through prematurity contribute to the association between non-transmitted common variants of PGS_{EA} and risk of neurodevelopmental conditions.

Supplementary Note 8: Role of PGS in modifying the penetrance of rare variants

Various lines of evidence from both neurodevelopmental conditions[68] and autism[38,69] cohorts and from population-based studies[70] suggest that incompletely penetrant inherited rare coding variants contribute to risk of rare neurodevelopmental conditions. A recent study found that undiagnosed rare disease patients with variants of unknown significance (VUS) had on average

more polygenic risk than their unaffected carrier parent, suggesting PGS might modify penetrance of VUS[71]. Rare protein-truncating variants (PTVs) and predicted damaging missense variants in constrained (loss-of-function-intolerant) genes[72] have been shown to act additively with PGSs on fluid intelligence and educational attainment within UK Biobank [73,74]. This implies that PGSs are likely to modify penetrance of these rare variants within families with neurodevelopmental conditions.

To test this, we used the whole-genome sequence data from GEL and exome sequence data from DDD to extract rare damaging coding variants from undiagnosed probands and their parents; specifically, we extracted heterozygous PTVs and predicted damaging missense variants with minor allele frequency < $1x10^{-4}$ within each cohort and < $1x10^{-5}$ in each gnomAD super-population in either dominant DD-associated genes (DDG2P)[75] with a loss-of-function mechanism, or in constrained genes (see **Methods**). Such variants are enriched in undiagnosed DDD cases compared to controls, and over-transmitted from unaffected parents to affected offspring[76]. We tested whether unaffected parents transmitting a damaging rare variant have significantly more protective PGSs than their affected children without a monogenic diagnosis. We observed nominally significant differences in $PGS_{NDC,DDD}$ between parents who transmitted a damaging rare variant in a dominant DD-associated gene and their children in a combined analysis of DDD and GEL (one-sided p=0.009, mean difference = -0.16 SD, N=186 pairs; **Supplementary Figure 8**). However, none of the differences passed multiple testing correction (p>0.05/[5 PGSs * 2 gene sets * 2 consequence classes]) (**Supplementary Figure 8**).

The interpretation of these results is potentially complicated by several factors, including the correlation between rare and common variants that is likely generated by parental assortment (**Figure 5**). Power may be reduced by our limited sample size and aggregation of rare variants with heterogeneous effects; it may be that many of the rare variants considered are not damaging, but due to their rarity, we aggregated across variants and genes to try to boost power. Additionally, if some parents actually show sub-clinical phenotypes as a result of these rare variants or polygenic burden, the inclusion of these parents in the analyses could be confounding these results.

Supplementary Note 9: Genes and pathways affected by common and rare variants associated with NDCs

We carried out several analyses to explore potential mechanistic overlap between the rare and common variants contributing to NDC risk. We note that recent work has demonstrated that common SNPs associated with educational attainment are enriched around autosomal dominant DDG2P genes[77], implying that the rare and common components converge on similar pathways. We started by taking a slightly different approach, taking 1,722 genes that had been prioritised with the DEPICT tool (at FDR < 5%) in the educational attainment GWAS we were using[56] ("EA genes"), and asking whether these showed a greater-than-expected overlap with 788 DDG2P genes responsible for at least one monogenic diagnosis amongst individuals with an NDC in DDD ("diagnostic DDG2P genes"; **Supplementary Methods**). We chose to focus on EA genes since the EA GWAS is strongly (negatively) genetically correlated with the NDC GWAS (**Figure 1**) but is so much more powerful than it; we do not have sufficient power to produce a plausible list of genes in which common variants predispose to NDCs from our own

GWAS, given we have only two genome-wide significant loci. We found that the EA genes were significantly enriched amongst diagnostic DDG2P genes compared to all autosomal protein-coding genes (odds ratio = 3.41; p-value = 1.2×10^{-36}; Fisher's exact test).

We next examined a collection of gene sets defined based on expression in prenatal or postnatal brain[24] and genes with cell type-specific expression in certain prenatal brain cell types[24] (24 gene sets). We tested whether each of these gene sets was enriched for EA genes or for diagnostic DDG2P genes using Fisher's exact test (**Supplementary Figure 18AB**). Compared to all remaining genes, both EA genes and diagnostic DDG2P genes showed significant enrichment amongst 7,373 genes preferentially expressed in prenatal brain ($p=3.5 \times 10^{-47}$ and $p=2.9 \times 10^{-31}$ respectively) but only EA genes showed significant enrichment amongst the 6,567 genes preferentially expressed in postnatal brain ($p=1.0 \times 10^{-8}$), which was significantly stronger than the enrichment of diagnostic DDG2P genes (z-score test $p = 8.9 \times 10^{-4}$; **Supplementary Figure 18A**). EA genes and/or diagnostic DDG2P genes were also significantly enriched amongst genes showing preferential expression in several of the particular cell types in the prenatal brain (**Supplementary Figure 18B**), but there were some differences. For example, diagnostic DDG2P genes but not EA genes were significantly enriched in several types of neural progenitor and stem cells (NEPRGC2, NEPRGC4), and this difference was significant (z-score test $p < 2.7 \times 10^{-4}$). Conversely, EA genes showed a significantly stronger degree of enrichment in several subtypes of excitatory neurons (ExN1, ExN2, ExN3) than diagnostic DDG2P genes (z-score test $p < 1.3 \times 10^{-6}$).

As an orthogonal approach to explore mechanistic overlap between the common and rare variant components, we applied stratified LD score regression[26] to the GWAS summary statistics for both NDCs and EA, to test whether the common variant signals were enriched around particular gene sets. For the EA GWAS, SNPs in or near diagnostic DDG2P genes (8.3% of SNPs) explain 13.0% (95% CI: 11.3–14.8%) of the total common SNP heritability, which is a significant enrichment (1.6-fold, $p=9 \times 10^{-8}$), in line with findings from Kingdom *et al.*[77]. For the NDC GWAS, the SNPs near diagnostic DDG2P genes explain 18.6% (95% CI: 3.4–33.8%) of the SNP heritability, but this is not a significant enrichment (2.3-fold, p=0.14), likely due to the low power of this GWAS. We also examined enrichment of SNP heritability in the aforementioned gene sets enriched in particular cell types in the prenatal brain. We focused on the EA GWAS since the NDC GWAS proved underpowered. The pattern of enrichment of SNP heritability across these cell types mimicked what we observed for enrichment of the genes defined by the EA GWAS, as expected, with significant enrichment in excitatory neurons and glial cells relative to other prenatal brain cell types but significant depletion in nascent neurons (NasN) relative to other prenatal brain cell types (**Supplementary Figure 18CD**).

Taken together, these results suggest that although rare and common variants predisposing to NDCs converge on similar sets of genes and are affecting many of the same cell types, the rare variants (affecting the diagnostic DDG2P genes) may be preferentially affecting very early brain development, whereas common variants may be preferentially affecting brain function slightly later in prenatal development and postnatally. In future, better-powered GWASs of NDCs will allow us to repeat these analyses using genes/SNPs implicated specifically in NDC risk rather than those ascertained for their associations with EA, as well as genes implicated through less penetrant rare variants, which may reveal different patterns.

Descriptions of Supplementary Tables

Supplementary Table 1. Number of samples used in each analysis. All individuals have genetically inferred European ancestry. Note that we did not calculate the PGS for neurodevelopmental conditions in UKHLS samples and DDD probands genotyped using CoreExome chip or Global Screening Array, since they were in the original GWAS (Niemi *et al*., Nature, 2018), but rather only tested it in GEL probands and in DDD probands who were genotyped using the OmniChip as well as in parents and UK birth cohorts. Scottish participants and those who are related to GEL participants were excluded from DDD when they are analysed together with GEL samples in a combined analysis.

Supplementary Table 2. Variance explained by polygenic scores (PGSs) for educational attainment (EA), cognitive performance (CP), the non-cognitive component of educational attainment (NonCogEA), schizophrenia (SCZ), rare neurodevelopmental conditions (NDC,DDD), as well as composite PGSs combining individual scores on the liability scale, either comparing DDD with UKHLS or GEL cases with GEL controls.

Supplementary Table 3. SNP heritability estimates using different methods. All estimates are on the liability scale assuming a population prevalence of 1%. LD score regression (LDSC) was run on summary statistics from the GEL-derived GWAS, DDD-derived GWAS, and the meta-analysed GWAS. LD- and MAF-stratified GREML (GREML-LDMS)[78] and phenotype-correlation genotype-correlation (PCGC)[79] regression were run in DDD and GEL GWAS samples separately, then the SNP heritability estimates were meta-analyzed. We observed higher SNP heritability estimates using GREML-LDMS and PCGC, possibly due to the fact that LDSC uses only the subset of SNPs in HapMap (**Methods**) and because the heritability estimate tends to be downward-biased at sample sizes of this order[80].

Supplementary Table 4. Genetic correlations (r_g) between neurodevelopmental conditions and other brain-related traits and disorders. We calculated r_g between brain-related traits and either GWAS of neurodevelopmental conditions derived from DDD or the GWAS meta-analysis of DDD and GEL using Linkage Disequilibrium Score Regression (LDSC). We also calculated r_g between the GWAS meta-analysis of neurodevelopmental conditions and those brain-related traits after conditioning on educational attainment and cognitive performance using GenomicSEM. We performed two-sided z-score tests ("z_pvalue") to compare r_g estimates before and after conditioning on the two traits. Confidence intervals (CI) were calculated using the standard error (se) of the r_g estimate. We corrected for 13 traits using the Bonferroni approach. For each external trait, we also report SNP heritability on the observed scale by LDSC. See **Supplementary Table 17** for information on external GWASs.

Supplementary Table 5. Two-sided *t*-tests comparing average polygenic scores (PGSs) between subsets of probands with neurodevelopmental conditions and control individuals shown in **Figure 2A**. The table contains comparisons (1) between subsets of probands with neurodevelopmental conditions (from DDD+GEL combined) and (2) between probands with neurodevelopmental conditions and control individuals from a combined set of unrelated individuals from GEL and UKHLS. The PGS for neurodevelopmental conditions derived from the GWAS of DDD and UKHLS samples was tested in only GEL samples and a held-out set in DDD. Nominally significant *t*-test results are indicated by one asterisk and tests that pass the

Bonferroni correction for five PGSs are indicated by two asterisks. The last column highlights comparisons that were mentioned in the main text.

Supplementary Table 6. Two-sided *t*-tests comparing polygenic scores (PGSs) between different control cohorts and subsets thereof, subsets of probands with neurodevelopmental conditions, and their parents in **Extended Data Figure 6**. The table contains comparisons (1) between MCS children before and after reweighting to adjust for sampling bias and attrition, (2) between probands with neurodevelopmental conditions or parents (from DDD+GEL combined) with reweighted MCS children, (3) between control subsets, (4) between probands with neurodevelopmental conditions or parents (from DDD+GEL combined) with each control subset, and (5) between trio and non-trio probands with neurodevelopmental conditions and those from birth cohorts. The PGS for neurodevelopmental conditions was derived from the GWAS of DDD and UKHLS samples so it was not tested in the GWAS sample. Nominally significant *t*-test results are indicated by one asterisk and tests that pass the Bonferroni correction for five PGSs are indicated by two asterisks.

Supplementary Table 7. Two-sided *t*-tests comparing average PGSs standardised using weighted MCS controls between parents and their affected offspring from different subsets of trios, and between probands with neurodevelopmental conditions or parents and control individuals, shown in **Figure 3B** and **Extended Data Figure 5**. PGSs were standardised so that MCS children reweighted to adjust for sampling bias and non-response bias had mean = 0 and standard deviation = 1 (**Supplementary Note 4**). More specifically, the table contains comparisons (1) between undiagnosed probands with unaffected parents and their parents (in DDD+GEL combined; **Figure 3B**), (2) between these two groups and control individuals (reweighted MCS children), (3) between diagnosed trios with affected parents and diagnosed trios with unaffected parents (**Extended Data Figure 5**), (4) between parents and probands in other subsets of trios (**Extended Data Figure 5**), and (5) between other subsets of probands with neurodevelopmental conditions or their parents and control individuals (reweighted MCS children; **Extended Data Figure 5**). The PGS for neurodevelopmental conditions derived from the GWAS of DDD and UKHLS samples was not tested in the GWAS samples. Nominally significant differences are indicated by one asterisk and tests that pass the Bonferroni correction for five PGSs are indicated by two asterisks.

Supplementary Table 8. Association between the polygenic score (PGS) for educational attainment (PGS_{EA}), factors affecting the chance of getting a monogenic diagnosis, and diagnostic status in DDD probands with white British ancestry affected by neurodevelopmental conditions (as shown in **Figure 2BC and Extended Data Figure 7B**). PGS_{EA} was regressed on the indicated variable in a linear regression when assessing its association with factors affecting the chance of getting a diagnosis. The probands' PGS_{EA} was tested in a maximum of 7,549 probands with neurodevelopmental conditions (without excluding Scottish samples). The fathers' or mothers' PGS_{EA} was tested in a maximum of 2497 trios. Nominally significant differences are indicated by one asterisk and tests that pass the Bonferroni correction of seven factors are indicated by two asterisks. We also estimated the effect size (in odds ratio) of each factor on the chance of getting a monogenic diagnosis in this subset of 7,549 probands with neurodevelopmental conditions, with and without controlling for proband's PGS_{EA}. In addition, we reported the effect size (in odds ratio) of PGS_{EA} on the chance of getting a monogenic diagnosis in the same probands, with and without controlling for these factors.

Supplementary Table 9. Polygenic transmission disequilibrium test (pTDT) in undiagnosed probands with unaffected parents, with or without excluding autistic probands. Mean pTDT deviation is calculated as the difference between the child's polygenic score and the mean parental polygenic score, in units of the SD of the latter. We tested if this is significantly different from 0 using two-sided one-sample *t*-tests.

Supplementary Table 10. Statistical results of the proband-only model and the trio model. All trios were used in the main analysis shown in **Figure 4** ("all"). In sensitivity analyses shown in **Supplementary Figure 4**, models were fitted to subgroups of cohorts: in GEL trios only ("GEL only"), in all cases with neurodevelopmental conditions versus GEL control trios ("NDC vs GEL control"), in all cases versus MCS control trios ("NDC vs MCS"), and in all cases versus ALSPAC control trios ("NDC vs ALSPAC"). To explore whether prematurity mediates the association between non-transmitted alleles of PGS_{EA} and risk of neurodevelopmental conditions, three models were run (**Supplementary Figure 5**): in DDD and MCS probands with available gestation age data ("DDD vs MCS with gestational age"), after controlling for whether or not the proband was born prematurely ("DDD vs MCS adj prematurity"), and in DDD and MCS probands excluding those who were born prematurely ("DDD vs MCS excluding premature probands"). Finally, extended proband only and trio models to control for variant burden scores (RVBS) were fitted to GEL trios ("proband only + RVBS" and "trio model + RVBS", respectively; **Extended Data Figure 10B**). CI indicates 95% confidence intervals. Nominally significant results are indicated by one asterisk, and associations that pass the Bonferroni correction for five polygenic scores are indicated by two asterisks.

Supplementary Table 11. Genetic correlations (r_g) between neurodevelopmental conditions and prenatal risk factors shown in **Extended Data Figure 9A**. We calculated r_g for the GWAS meta-analysis of DDD and GEL using Linkage Disequilibrium Score Regression (LDSC). We also calculated r_g between the meta-GWAS and those risk factors after conditioning on educational attainment and cognitive performance using GenomicSEM. Confidence intervals (CI) were calculated using the standard error (se) of the r_g estimate. We corrected for five traits using the Bonferroni approach. For each external trait, we also report SNP heritability on the observed scale estimated by LDSC.

Supplementary Table 12. Pearson correlation coefficients between PGSs and the number of inherited rare coding variants in different subsets of trios with neurodevelopmental conditions and MCS trios. We conducted the analysis in trios with neurodevelopmental conditions in which both parents were unaffected ("NDC trios with unaffected parents"), in undiagnosed trios with unaffected parents ("undiagnosed NDC trios with unaffected parents"), and in trios with *de novo* diagnoses and unaffected parents ("De novo diagnoses; parents unaffected") (**Figure 5**; **Supplementary Figure 6**). In MCS trios, we calculated the unweighted ("MCS trios; unweighted") and weighted correlations after adjusting for sampling bias and non-response bias ("MCS trios; weighted") (**Supplementary Figure 7**). Rare variant burden scores (RVBSs) were calculated using PTVs, PTV and missense variants combined, or synonymous variants in dominant DD genes with a loss-of-function mechanism or in constrained genes. We calculated correlations within probands (i.e. the child's RVBS, with their own PGS; "child" in the "person type" column), and those within their parents ("same parent"). We also calculated the cross-parental correlation (i.e. one parent's RVBS with the other parent's PGS; "partner"). CI indicates 95% confidence intervals. Significant correlations that pass Bonferroni correction for 30 tests

(P-value < 0.0017; five PGSs, three variant types, and two gene sets) are indicated by two asterisks, and nominally significant correlations (P-value < 0.05) are indicated by one asterisk.

Supplementary Table 13: Number of samples and variants remaining after each step of quality control for two batches of Global Screening Array data from DDD samples, prior to merging.

Supplementary Table 14. Number of samples and variants remaining after each step of quality control for Global Screening Array data from DDD samples, after merging the two initial batches.

Supplementary Table 15. Variant quality control filters applied to whole genome sequence data from the Genomics England 100,000 Genomes variant callset known as "aggV2". We used variants that passed all these filters ("PASS" variants).

Supplementary Table 16. Variant quality control filters applied to whole-exome sequence data from DDD, for the analyses of polygenic scores modifying penetrance of rare coding variants and correlations between polygenic scores and rare variant burden.

Supplementary Table 17. Previously-published GWASs used to calculate genetic correlations and/or polygenic scores, including the number of SNPs in the polygenic score.

Supplementary Table 18. Pearson correlations between the five polygenic scores (PGS) used throughout this paper. Correlations were estimated in the following three subgroups: probands with neurodevelopmental conditions regardless of trio status (N=3,618 from GEL and N=6,883 from DDD; N=597 in DDD excluding GWAS samples), parents of probands from 2,174 DDD trios and 2,390 GEL trios, and controls individuals from GEL (N=13,667) and UKHLS (N=9,270). P-values lower than the minimum value that R can display are indicated as "< 2.2E-308". Significant correlations that pass Bonferroni correction for five PGSs are indicated by two asterisks, and nominally significant correlations (P-value < 0.05) are indicated by one asterisk.

Supplementary Table 19. Investigation of PGSs modifying penetrance of rare damaging coding variants within families. We performed one-sided, paired *t*-tests to compare PGSs between unaffected parents transmitting damaging rare variants and their undiagnosed children. Sample size shows the number of parent-proband pairs. A positive difference indicates that the unaffected parents have higher PGS than the children. One asterisk indicates a nominally significant difference; none of the differences passed Bonferroni correction for 20 tests (five PGSs, two variant types, and two gene sets). The upper bound of the 95% confidence interval (CI) was calculated for PGS_{SCZ} and $PGS_{NDC,DDD}$, for which an upper-tailed test was used and the lower bound is -infinity. The lower bound of CI was calculated for PGS_{EA}, PGS_{CP}, and $PGS_{NonCogEA}$, for which a lower-tailed test was used, and the upper bound is +infinity.

Supplementary Table 20. Enrichment of NDC risk genes implicated via common versus rare variants in brain cell types. We considered 1,722 EA GWAS genes prioritised by Lee *et al.* 2018 and 788 DDG2P genes in which a diagnosis has been found for any DDD proband with a neurodevelopmental condition. We tested the enrichment of these two gene sets in genes that show particularly high expression in the indicated cell types relative to other cell types in the prenatal brain (**Supplementary Figure 18B**). Fisher's exact tests were used to calculate the enrichment (odds ratios) and p-values ("p_fishers"). Two-sided z-score tests were used to compare enrichment estimates of DDG2P genes and EA GWAS genes ("p_z_score"). IPC:

intermediate progenitor cells; NEPRGC: neural epithelial progenitor/radial glial lineage; ExN: excitatory neurons; InN: interneurons; NasN: nascent neurons; Astro: astroglial lineage; Oligo: oligodendrocytes; OPC: oligodendrocyte progenitor cells; Endo: endothelial cells.

Supplementary Table 21. Enrichment of heritability of educational attainment attributable to SNPs in or near cell type-enriched genes. We used genes that show particularly high expression in the indicated cell types relative to other cell types in the prenatal brain**.** We applied stratified LD score regression to GWAS summary statistics of educational attainment (**Supplementary Figure 19CD**). Enrichment was estimated as the proportion of heritability explained by SNPs in or near prenatal brain cell type-enriched genes divided by the proportion of SNPs mapping to these regions. SE: standard error; IPC: intermediate progenitor cells; NEPRGC: neural epithelial progenitor/radial glial lineage; ExN: excitatory neurons; InN: interneurons; NasN: nascent neurons; Astro: astroglial lineage; Oligo: oligodendrocytes; OPC: oligodendrocyte progenitor cells; Endo: endothelial cells.

Descriptions of Supplementary Data

Supplementary Data 1. Summary statistics from the GWAS of neurodevelopmental conditions comparing cases to controls within the Genomics England (GEL) 100,000 Genomes Project.

Supplementary Data 2. Summary statistics from the GWAS of neurodevelopmental conditions comparing DDD cases to UKHLS controls, excluding the Scottish samples from DDD.

Supplementary Data 3. Summary statistics from the GWAS meta-analysis of neurodevelopmental conditions combining the DDD and GEL GWASs.

Supplementary References

1. Niemi, M. E. K. *et al.* Common genetic variants contribute to risk of rare severe neurodevelopmental disorders. *Nature* **562**, 268–271 (2018).
2. Deciphering Developmental Disorders Study. Large-scale discovery of novel genetic causes of developmental disorders. *Nature* **519**, 223–228 (2015).
3. 1000 Genomes Project Consortium *et al.* A global reference for human genetic variation. *Nature* **526**, 68–74 (2015).
4. Patterson, N., Price, A. L. & Reich, D. Population structure and eigenanalysis. *PLoS Genet.* **2**, e190 (2006).
5. Price, A. L. *et al.* Long-range LD can confound genome scans in admixed populations. *Am. J. Hum. Genet.* **83**, 132–5; author reply 135–9 (2008).
6. McInnes, L., Healy, J. & Melville, J. UMAP: Uniform Manifold Approximation and Projection for Dimension Reduction. *arXiv [stat.ML]* (2018).
7. Kousathanas, A. *et al.* Whole-genome sequencing reveals host factors underlying critical COVID-19. *Nature* **607**, 97–103 (2022).

8. Purcell, S. *et al.* PLINK: a tool set for whole-genome association and population-based linkage analyses. *Am. J. Hum. Genet.* **81**, 559–575 (2007).
9. Yang, J., Lee, S. H., Goddard, M. E. & Visscher, P. M. GCTA: a tool for genome-wide complex trait analysis. *Am. J. Hum. Genet.* **88**, 76–82 (2011).
10. Boyd, A. *et al.* Cohort Profile: the 'children of the 90s'--the index offspring of the Avon Longitudinal Study of Parents and Children. *Int. J. Epidemiol.* **42**, 111–127 (2013).
11. Manichaikul, A. *et al.* Robust relationship inference in genome-wide association studies. *Bioinformatics* **26**, 2867–2873 (2010).
12. Fitzsimons, E. *et al.* Collection of genetic data at scale for a nationally representative population: the UK Millennium Cohort Study. *Longit. Life Course Stud.* **13**, 169–187 (2021).
13. Mills, R. E. *et al.* An initial map of insertion and deletion (INDEL) variation in the human genome. *Genome Res.* **16**, 1182–1190 (2006).
14. Demange, P. A. *et al.* Investigating the genetic architecture of noncognitive skills using GWAS-by-subtraction. *Nat. Genet.* **53**, 35–44 (2021).
15. Vilhjálmsson, B. J. *et al.* Modeling Linkage Disequilibrium Increases Accuracy of Polygenic Risk Scores. *Am. J. Hum. Genet.* **97**, 576–592 (2015).
16. International HapMap 3 Consortium *et al.* Integrating common and rare genetic variation in diverse human populations. *Nature* **467**, 52–58 (2010).
17. Bycroft, C. *et al.* The UK Biobank resource with deep phenotyping and genomic data. *Nature* **562**, 203–209 (2018).
18. Lee, J. J. *et al.* Gene discovery and polygenic prediction from a genome-wide association study of educational attainment in 1.1 million individuals. *Nat. Genet.* **50**, 1112–1121 (2018).
19. Trubetskoy, V. *et al.* Mapping genomic loci implicates genes and synaptic biology in schizophrenia. *Nature* **604**, 502–508 (2022).
20. Howe, L. J. *et al.* Within-sibship genome-wide association analyses decrease bias in estimates of direct genetic effects. *Nat. Genet.* **54**, 581–592 (2022).
21. Wright, C. F. *et al.* Genomic Diagnosis of Rare Pediatric Disease in the United Kingdom and Ireland. *N. Engl. J. Med.* **388**, 1559–1571 (2023).
22. Plewis, I. The Millennium Cohort Study: Technical Report on Sampling (4th Edition). http://doc.ukdataservice.ac.uk/doc/4683/mrdoc/pdf/mcs_technical_report_on_sampling_4th_edition.pdf (2007).
23. Plewis, I. Non-Response in a Birth Cohort Study: The Case of the Millennium Cohort Study. *Int. J. Soc. Res. Methodol.* **10**, 325–334 (2007).
24. Li, M. *et al.* Integrative functional genomic analysis of human brain development and neuropsychiatric risks. *Science* **362**, (2018).
25. Warrier, V. *et al.* Genetic insights into human cortical organization and development through genome-wide analyses of 2,347 neuroimaging phenotypes. *Nat. Genet.* **55**, 1483–1493 (2023).
26. Finucane, H. K. *et al.* Partitioning heritability by functional annotation using genome-wide association summary statistics. *Nat. Genet.* **47**, 1228–1235 (2015).
27. Russell, G. *et al.* Time trends in autism diagnosis over 20 years: a UK population-based cohort study. *J. Child Psychol. Psychiatry* **63**, 674–682 (2022).
28. Davies, G. *et al.* Study of 300,486 individuals identifies 148 independent genetic loci influencing general cognitive function. *Nat. Commun.* **9**, 2098 (2018).
29. Savage, J. E. *et al.* Genome-wide association meta-analysis in 269,867 individuals identifies new genetic and functional links to intelligence. *Nat. Genet.* **50**, 912–919 (2018).
30. Williams, C. M., Labouret, G., Wolfram, T., Peyre, H. & Ramus, F. A General Cognitive Ability Factor for the UK Biobank. *Behav. Genet.* **53**, 85–100 (2023).
31. Yengo, L. *et al.* A saturated map of common genetic variants associated with human height. *Nature* **610**, 704–712 (2022).
32. Perry, J. R. *et al.* Parent-of-origin-specific allelic associations among 106 genomic loci for age at menarche. *Nature* **514**, 92–97 (2014).

33. Knies, G. Understanding society: waves 1–7, 2009–2016 and harmonised BHPS: waves 1–18, 1991–2009, user guide. *Colchester: University of Essex*.

34. of Essex, U. Institute for Social and Economic Research and National Centre for Social Research, Understanding Society: Waves 2 and 3 Nurse Health Assessment …. *UK Data Service*.

35. Fraser, A. *et al.* Cohort Profile: the Avon Longitudinal Study of Parents and Children: ALSPAC mothers cohort. *Int. J. Epidemiol.* **42**, 97–110 (2013).

36. Mostafa, T. & Ploubidis, G. Millennium Cohort Study. https://discovery.ucl.ac.uk/id/eprint/10060140/1/mcs6_report_on_response.pdf.

37. Morelli, S., Nolan, B., Palomino, J. C. & Van Kerm, P. The Wealth (Disadvantage) of Single-Parent Households. *Ann. Am. Acad. Pol. Soc. Sci.* **702**, 188–204 (2022).

38. Fu, J. M. *et al.* Rare coding variation provides insight into the genetic architecture and phenotypic context of autism. *Nat. Genet.* **54**, 1320–1331 (2022).

39. Satterstrom, F. K. *et al.* Large-Scale Exome Sequencing Study Implicates Both Developmental and Functional Changes in the Neurobiology of Autism. *Cell* **180**, 568–584.e23 (2020).

40. Sanders, S. J. *et al.* Insights into Autism Spectrum Disorder Genomic Architecture and Biology from 71 Risk Loci. *Neuron* **87**, 1215–1233 (2015).

41. Sanders, S. J. *et al.* De novo mutations revealed by whole-exome sequencing are strongly associated with autism. *Nature* **485**, 237–241 (2012).

42. Ronemus, M., Iossifov, I., Levy, D. & Wigler, M. The role of de novo mutations in the genetics of autism spectrum disorders. *Nat. Rev. Genet.* **15**, 133–141 (2014).

43. Iossifov, I. *et al.* The contribution of de novo coding mutations to autism spectrum disorder. *Nature* **515**, 216–221 (2014).

44. Wigdor, E. M. *et al.* The female protective effect against autism spectrum disorder. *Cell Genomics* **2**, 100134 (2022).

45. Robinson, E. B., Lichtenstein, P., Anckarsäter, H., Happé, F. & Ronald, A. Examining and interpreting the female protective effect against autistic behavior. *Proc. Natl. Acad. Sci. U. S. A.* **110**, 5258–5262 (2013).

46. Baron-Cohen, S. *et al.* Prevalence of autism-spectrum conditions: UK school-based population study. *Br. J. Psychiatry* **194**, 500–509 (2009).

47. Fombonne, E. Epidemiology of pervasive developmental disorders. *Pediatr. Res.* **65**, 591–598 (2009).

48. Kaplanis, J. *et al.* Evidence for 28 genetic disorders discovered by combining healthcare and research data. *Nature* **586**, 757–762 (2020).

49. Warrier, V. *et al.* Genetic correlates of phenotypic heterogeneity in autism. *Nat. Genet.* **54**, 1293–1304 (2022).

50. Antaki, D. *et al.* A phenotypic spectrum of autism is attributable to the combined effects of rare variants, polygenic risk and sex. *Nat. Genet.* 1–9 (2022).

51. Haworth, S. *et al.* Apparent latent structure within the UK Biobank sample has implications for epidemiological analysis. *Nat. Commun.* **10**, 333 (2019).

52. Yengo, L. *et al.* Imprint of assortative mating on the human genome. *Nat Hum Behav* **2**, 948–954 (2018).

53. Kong, A. *et al.* The nature of nurture: Effects of parental genotypes. *Science* **359**, 424–428 (2018).

54. Kerminen, S. *et al.* Geographic Variation and Bias in the Polygenic Scores of Complex Diseases and Traits in Finland. *Am. J. Hum. Genet.* **104**, 1169–1181 (2019).

55. Young, A. I. *et al.* Mendelian imputation of parental genotypes improves estimates of direct genetic effects. *Nat. Genet.* **54**, 897–905 (2022).

56. Lee, J. J. *et al.* Gene discovery and polygenic prediction from a 1.1-million-person GWAS of educational attainment. *Nat. Genet.* **50**, 1112 (2018).

57. Joseph, R. M. *et al.* Neurocognitive and Academic Outcomes at Age 10 Years of Extremely Preterm Newborns. *Pediatrics* **137**, (2016).

58. Aarnoudse-Moens, C. S. H., Weisglas-Kuperus, N., van Goudoever, J. B. & Oosterlaan, J. Meta-

analysis of neurobehavioral outcomes in very preterm and/or very low birth weight children. *Pediatrics* **124**, 717–728 (2009).

59. Huang, J., Zhu, T., Qu, Y. & Mu, D. Prenatal, Perinatal and Neonatal Risk Factors for Intellectual Disability: A Systemic Review and Meta-Analysis. *PLoS One* **11**, e0153655 (2016).
60. Solé-Navais, P. *et al.* Genetic effects on the timing of parturition and links to fetal birth weight. *Nat. Genet.* **55**, 559–567 (2023).
61. Kandel, D. B., Griesler, P. C. & Schaffran, C. Educational attainment and smoking among women: risk factors and consequences for offspring. *Drug Alcohol Depend.* **104 Suppl 1**, S24–33 (2009).
62. Goldenberg, R. L., Culhane, J. F., Iams, J. D. & Romero, R. Epidemiology and causes of preterm birth. *Lancet* **371**, 75–84 (2008).
63. Saunders, G. R. B. *et al.* Genetic diversity fuels gene discovery for tobacco and alcohol use. *Nature* **612**, 720–724 (2022).
64. Honigberg, M. C. *et al.* Polygenic prediction of preeclampsia and gestational hypertension. *Nat. Med.* **29**, 1540–1549 (2023).
65. Xu, T., Feng, Y., Peng, H., Guo, D. & Li, T. Obstructive sleep apnea and the risk of perinatal outcomes: a meta-analysis of cohort studies. *Sci. Rep.* **4**, 6982 (2014).
66. Campos, A. I. *et al.* Discovery of genomic loci associated with sleep apnea risk through multi-trait GWAS analysis with snoring. *Sleep* **46**, (2023).
67. Pamidi, S. *et al.* Maternal sleep-disordered breathing and adverse pregnancy outcomes: a systematic review and metaanalysis. *Am. J. Obstet. Gynecol.* **210**, 52.e1–52.e14 (2014).
68. Wright, C. F. *et al.* Evaluating variants classified as pathogenic in ClinVar in the DDD Study. *Genet. Med.* **23**, 571–575 (2021).
69. Wilfert, A. B. *et al.* Recent ultra-rare inherited variants implicate new autism candidate risk genes. *Nat. Genet.* **53**, 1125–1134 (2021).
70. Kingdom, R. *et al.* Rare genetic variants in genes and loci linked to dominant monogenic developmental disorders cause milder related phenotypes in the general population. *The American Journal of Human Genetics* Preprint at https://doi.org/10.1016/j.ajhg.2022.05.011 (2022).
71. Smail, C. *et al.* Complex trait associations in rare diseases and impacts on Mendelian variant interpretation. *medRxiv* 2024.01.10.24301111 (2024) doi:10.1101/2024.01.10.24301111.
72. Lek, M. *et al.* Analysis of protein-coding genetic variation in 60,706 humans. *Nature* **536**, 285–291 (2016).
73. Chen, C.-Y. *et al.* The impact of rare protein coding genetic variation on adult cognitive function. *Nat. Genet.* **55**, 927–938 (2023).
74. Kingdom, R., Beaumont, R. N., Wood, A. R., Weedon, M. N. & Wright, C. F. Genetic modifiers of rare variants in monogenic developmental disorder loci. *medRxiv* (2022) doi:10.1101/2022.12.15.22283523.
75. DECIPHER: Database of Chromosomal Imbalance and Phenotype in Humans Using Ensembl Resources. *Am. J. Hum. Genet.* **84**, 524–533 (2009).
76. Samocha, K. E. *et al.* Substantial role of rare inherited variation in individuals with developmental disorders. *medRxiv* 2024.08.28.24312746 (2024) doi:10.1101/2024.08.28.24312746.
77. Kingdom, R., Beaumont, R. N., Wood, A. R., Weedon, M. N. & Wright, C. F. Genetic modifiers of rare variants in monogenic developmental disorder loci. *Nat. Genet.* **56**, 861–868 (2024).
78. Yang, J. *et al.* Genetic variance estimation with imputed variants finds negligible missing heritability for human height and body mass index. *Nat. Genet.* **47**, 1114–1120 (2015).
79. Golan, D., Lander, E. S. & Rosset, S. Measuring missing heritability: inferring the contribution of common variants. *Proc. Natl. Acad. Sci. U. S. A.* **111**, E5272–81 (2014).
80. Churchhouse, C. Insights from estimates of SNP-heritability for >2,000 traits and disorders in UK Biobank. *Neale lab* http://www.nealelab.is/blog/2017/9/20/insights-from-estimates-of-snp-heritability-for-2000-traits-and-disorders-in-uk-biobank (2017).
