# Supplementary material for: Examining the role of common variants in rare neurodevelopmental conditions
Source: Nature. 2024 Nov 20;636(8042):404–11. doi: 10.1038/s41586-024-08217-y (PMC11634775; doi:10.1038/s41586-024-08217-y)
Supplement: Supplementary file 2 — Reporting Summary [file 41586_2024_8217_MOESM2_ESM.pdf]

Corresponding author(s): Hilary Martin

Last updated by author(s): Sep 16, 2024

Reporting Summary

Nature Portfolio wishes to improve the reproducibility of the work that we publish. This form provides structure for consistency and transparency in reporting. For further information on Nature Portfolio policies, see our Editorial Policies and the Editorial Policy Checklist.

Statistics

For all statistical analyses, confirm that the following items are present in the figure legend, table legend, main text, or Methods section.

n/a	Confirmed	
☐	☒	The exact sample size (*n*) for each experimental group/condition, given as a discrete number and unit of measurement
☒	☐	A statement on whether measurements were taken from distinct samples or whether the same sample was measured repeatedly
☐	☒	The statistical test(s) used AND whether they are one- or two-sided<br>*Only common tests should be described solely by name; describe more complex techniques in the Methods section.*
☐	☒	A description of all covariates tested
☐	☒	A description of any assumptions or corrections, such as tests of normality and adjustment for multiple comparisons
☐	☒	A full description of the statistical parameters including central tendency (e.g. means) or other basic estimates (e.g. regression coefficient) AND variation (e.g. standard deviation) or associated estimates of uncertainty (e.g. confidence intervals)
☐	☒	For null hypothesis testing, the test statistic (e.g. *F*, *t*, *r*) with confidence intervals, effect sizes, degrees of freedom and *P* value noted<br>*Give P values as exact values whenever suitable.*
☒	☐	For Bayesian analysis, information on the choice of priors and Markov chain Monte Carlo settings
☒	☐	For hierarchical and complex designs, identification of the appropriate level for tests and full reporting of outcomes
☐	☒	Estimates of effect sizes (e.g. Cohen's *d*, Pearson's *r*), indicating how they were calculated

Our web collection on statistics for biologists contains articles on many of the points above.

Software and code

Policy information about availability of computer code

Data collection	No software was used for Data Collection.
Data analysis	Plink (v1.9) were used to process genotype array data and perform PCA and GWAS. GCTA (v1.94.1) was used to perform projection PCA. KING (v2.2.4) was used to estimate kinship relationships in genotype array samples. The "umap" R package (v0.2.4.1) was used to assign individuals to genetically inferred ancestry groups. Bcftools (v1.16) were used to process sequence data. Plink (v2.0) was used by GEL team to estimate pairwise kinship relationships using the KING robust algorithm. Python (v3.7.0) was used to extract rare variants from sequence data. Hail v0.2.105 was used to perform QC of exome sequence data in birth cohorts. Metal (the version released on 2011-03-25) was used to perform the GWAS meta-analysis. LDpred (v1.0.11) was used to generate SNP weights in PGS. SNP heritability was estimated using LDSC (v1.0.1), GCTA (v1.94.1), and PCGC regression implemented in LDAK (v5.2). LDSC (v1.0.1) and GenomicSEM (0.0.5c) were used to estimate genetic correlations. Remaining analyses were performed in R (4.0.2).

For manuscripts utilizing custom algorithms or software that are central to the research but not yet described in published literature, software must be made available to editors and reviewers. We strongly encourage code deposition in a community repository (e.g. GitHub). See the Nature Portfolio guidelines for submitting code & software for further information.

Data

Policy information about availability of data

All manuscripts must include a data availability statement. This statement should provide the following information, where applicable:

- Accession codes, unique identifiers, or web links for publicly available datasets
- A description of any restrictions on data availability
- For clinical datasets or third party data, please ensure that the statement adheres to our policy

The raw and post-quality control genotype array data and exome sequence data from DDD are available through European Genome-phenome Archive, under EGAS00001000775. Whole-genome sequence data and phenotypic data from the 100,000 Genomes project can be accessed by application to Genomics England (https://www.genomicsengland.co.uk/research/academic/join-gecip). GWAS summary statistics of neurodevelopmental conditions generated in this study are available in Supplementary Data. Researchers can apply to access genotype array data from UKHLS (https://www.understandingsociety.ac.uk/documentation/access-data/), ALSPAC (https://www.bristol.ac.uk/alspac/researchers/access/), and MCS (https://cls.ucl.ac.uk/data-access-training/data-access/). Publicly available GWAS summary statistics can be accessed at various resources: http://www.thessgac.org/data, https://pgc.unc.edu/for-researchers/download-results/, and https://egg-consortium.org/Gestational-duration-2023.html. DDG2P genes can be downloaded at https://www.deciphergenomics.org/ddd/ddgenes.

Research involving human participants, their data, or biological material

Policy information about studies with human participants or human data. See also policy information about sex, gender (identity/presentation), and sexual orientation and race, ethnicity and racism.

Reporting on sex and gender	We used biological sex reported by clinicians, participants, or parents of patients. We removed participants when their sex inferred by genetic data is not consistent with the reported sex.<br>Majority of the analyses were performed in both sex combined, and sex was corrected as a covariate when appropriate (e.g. GWAS). We also compared polygenic scores between sexes and performed the pTDT analysis in a sex-specific manner. Sample sizes can be found in the Methods section.
Reporting on race, ethnicity, or other socially relevant groupings	We focused on individuals of white British ancestry, which was defined by genetic similarity to British individuals from the 1,000 Genomes Project. Self-reported ethnicity was also available in the MCS cohort, and we further restricted to individuals who self-reported as being of White ethnicity. We use 20 genetic principal components to adjust for remaining fine-scale population structure.
Population characteristics	We restricted our analyses to participants with genome-wide genotype or whole-genome sequence data available. Patients have been diagnosed with neurodevelopmental conditions, and about 40% of them had a monogenic diagnosis. Age of onset is <16 years old. Genetic data of both parents of 35% of the DDD patients and 60% of GEL patients were available. Unaffected parent-offspring trios were from two UK birth cohorts: ALSPAC where the children were born between 1991 and 1992, and MCS where the children were born between 2000 and 2001.<br>A more detailed description of each cohort can be found in the "Cohort Descriptions and phenotypes" section in the paper.
Recruitment	DDD patients affected by developmental conditions and parents were recruited by clinical geneticists across the UK, between 2011 and 2015. The 100,000 Genomes project recruited rare disease families and cancer patients through NHS. The UKHLS cohort aimed to capture a representative sample of people living in the UK and to collect longitudinal socioeconomic and other data on them. The ALSPAC cohort recruited families in the Avon region of southwest England. The MCS cohort recruited families all over the UK, and children living in disadvantaged areas were intentionally over-sampled.<br>A detailed description of recruitment of cohorts and potential biases inherent in these can be found in Supplementary Note 4. In summary, the estimation of the effect size of polygenic scores (particularly for educational attainment) is sensitive to the choice of controls. For example, the differences in PGS between patients and unaffected controls would be larger if the control cohort is biased towards individuals with higher socio-economic status (SES). Volunteer-based cohorts such as ALSPAC and UKHLS show on average higher SES than the general UK population, while control individuals from GEL, recruited through the National Health Service, have lower SES than ALSPAC and UKHLS. In MCS, individuals from disadvantaged areas were over-sampled. However, the weights we developed to correct for recruitment bias and non-response bias should help to mitigate the biases.
Ethics oversight	The DDD study has UK Research Ethics Committee approval (10/H0305/83, granted by the Cambridge South Research Ethics Committee and GEN/284/12, granted by the Republic of Ireland Research Ethics Committee). The 100,000 Genomes project was approved by the East of England—Cambridge Central Research Ethics Committee (REF 20/EE/0035). Ethical approval for ALSPAC was obtained from the ALSPAC Ethics and Law Committee and the Local Research Ethics Committees. Ethical approval for each sweep of MCS was obtained from NHS Research Ethics Committees (MREC). Ethical approval for the sixth MCS sweep - which included the collection of saliva samples from children and biological resident parents - was obtained from London-Central REC (MREC; 13/LO/1786).

Note that full information on the approval of the study protocol must also be provided in the manuscript.

Field-specific reporting

Please select the one below that is the best fit for your research. If you are not sure, read the appropriate sections before making your selection.

☒ Life sciences ☐ Behavioural & social sciences ☐ Ecological, evolutionary & environmental sciences

For a reference copy of the document with all sections, see nature.com/documents/nr-reporting-summary-flat.pdf

Life sciences study design

All studies must disclose on these points even when the disclosure is negative.

Sample size	The sample size was determined by the maximum subset of unrelated individuals who had both post-quality-control genotype and phenotype data in each cohort. We did not perform a sample size calculation; instead, we used as many samples as were available in the existing rare disease and control cohorts. The sample sizes in our GWAS exceed the minimum sample size recommended by authors of software that we used to estimate SNP heritability: 5000 for LD score regression, 3160 for GCTA-LDMS, and 7000 for PCGC. For some analyses, we are uncertain if our sample size is sufficient to detect smaller effects, as we do not have relevant estimates from previous literature.
Data exclusions	We excluded participants who were not identified as having white British ancestry using genetic data. To get unbiased estimates, we excluded one individual from each pair of related individuals (up to third-degree relatives).
Replication	We replicated the findings of polygenic signals (more specifically, the association between neurodevelopmental conditions and polygenic score for relevant traits) observed in the DDD cohort in the 100,000 Genomes project. For downstream analysis, we thus combined the two cohorts, or performed a meta-analysis. We did not seek replication in a third rare disease cohort, due to lack of similar cohorts in the UK. However, we did use different control cohorts, such as UK birth cohorts in addition to UKHLS and GEL. Patients consistently show significantly lower polygenic scores for educational attainment compared to all control cohorts (Extended Data Figure 6). Additionally, the parental non-transmitted coefficients for the EA PGS in trio models are significant regardless of the control cohorts used (Supplementary Figure 4), indicating the robust role of common variants in rare neurodevelopmental conditions.
Randomization	No randomisation of participants was performed in this study. In association analyses, we controlled for genetic principal components and sex as covariates.
Blinding	Blinding was not possible because analysts needed to use the phenotype data in the analysis, or perform analysis in a subset of the participants with a certain characteristic (e.g patients with or without a monogenic diagnosis).

Reporting for specific materials, systems and methods

We require information from authors about some types of materials, experimental systems and methods used in many studies. Here, indicate whether each material, system or method listed is relevant to your study. If you are not sure if a list item applies to your research, read the appropriate section before selecting a response.

Materials & experimental systems

n/a	Involved in the study
☒	☐ Antibodies
☒	☐ Eukaryotic cell lines
☒	☐ Palaeontology and archaeology
☒	☐ Animals and other organisms
☒	☐ Clinical data
☒	☐ Dual use research of concern
☒	☐ Plants

Methods

n/a	Involved in the study
☒	☐ ChIP-seq
☒	☐ Flow cytometry
☒	☐ MRI-based neuroimaging

Plants

Seed stocks	*Report on the source of all seed stocks or other plant material used. If applicable, state the seed stock centre and catalogue number. If plant specimens were collected from the field, describe the collection location, date and sampling procedures.*
Novel plant genotypes	*Describe the methods by which all novel plant genotypes were produced. This includes those generated by transgenic approaches, gene editing, chemical/radiation-based mutagenesis and hybridization. For transgenic lines, describe the transformation method, the number of independent lines analyzed and the generation upon which experiments were performed. For gene-edited lines, describe the editor used, the endogenous sequence targeted for editing, the targeting guide RNA sequence (if applicable) and how the editor was applied.*
Authentication	*Describe any authentication procedures for each seed stock used or novel genotype generated. Describe any experiments used to assess the effect of a mutation and, where applicable, how potential secondary effects (e.g. second site T-DNA insertions, mosiacism, off-target gene editing) were examined.*
